# Immune correlates analysis of the Imbokodo (HVTN 705/HPX2008) efficacy trial of a mosaic HIV-1 vaccine regimen evaluated in Southern African people assigned female sex at birth: a two-phase case-control study

**DOI:** 10.1016/j.ebiom.2024.105320

**Published:** 2024-09-04

**Authors:** Avi Kenny, Janine van Duijn, One Dintwe, Jack Heptinstall, Randy Burnham, Sheetal Sawant, Lu Zhang, Dieter Mielke, Sharon Khuzwayo, Faatima Laher Omar, Sherry Stanfield-Oakley, Taylor Keyes, Brooke Dunn, Derrick Goodman, Youyi Fong, David Benkeser, Rodger Zou, John Hural, Ollivier Hyrien, Michal Juraska, Alex Luedtke, Lars van der Laan, Elena E. Giorgi, Craig Magaret, Lindsay N. Carpp, Laura Pattacini, Tom van de Kerkhof, Bette Korber, Wouter Willems, Leigh H. Fisher, Hanneke Schuitemaker, Edith Swann, James G. Kublin, Maria G. Pau, Susan Buchbinder, Frank Tomaka, Steven Nijs, Ludo Lavreys, Huub C. Gelderblom, Lawrence Corey, Kathryn Mngadi, Glenda E. Gray, Erica Borducchi, Jenny Hendriks, Kelly E. Seaton, Susan Zolla-Pazner, Dan H. Barouch, Guido Ferrari, Stephen C. De Rosa, M Juliana McElrath, Erica Andersen-Nissen, Daniel J. Stieh, Georgia D. Tomaras, Peter B. Gilbert, Imbokodo Study and Correlates Group, Jon Allagappen, Jessica Andriesen, Alison Ayres, Saman Baral, Linda-Gail Bekker, Asiphe Besethi, Caroline Borremans, Esmee Braams, Caroline Brackett, William Brumskine, Roma Chilengi, Rachel Choi, Thozama Dubula, Jaiden Seongmi Dumas, Brooke Dunn, Radhika Etikala, Zelda Euler, Sarah Everett, Nigel Garrett, Huub Gelderblom, Katherine Gill, Kevin Gillespie, Dimitri Goedhart, Erik Goosmann, Shannon Grant, Ellie Hands, Barton Haynes, Bronwill Herringer, Zaheer Hoosain, Mina Hosseinipour, Portia Hunidzarira, Julia Hutter, Mubiana Inambao, Craig Innes, Taylor Keyes, William Kilembe, Philippus Kotze, Sheena Kotze, Fatima Laher, Imre Laszlo, Erica Lazarus, Hua-Xin Liao, Yong Lin, Helen Lu, Judith Lucas, Mookho Malahleha, Tara McNair, Peter Meerts, Zinhle Mgaga, Mahlodi Montlha, Boitumelo Mosito, Andrew Moultrie, Sarah Mudrak, Valérie Oriol-Mathieu, Marcella Sarzotti-Kelsoe, Matson Tso Mathebula, Mitch Matoga, Rachael McClennen, Pamela Mda, Peter Meerts, Vimla Naicker, Logashvari Naidoo, Cindy-Ann Okkers, Saleha Omarjee, Hella Pasmans, Tricia Philip, Abraham Pinter, Annah Pitsi, Ornelia Ramos, April Randhawa, Sanne Roels, Shamiska Rohith, Lucy Rutten, Jerald Sadoff, Gabriela Salinas, Yvonne Salzgeber, Lorenz Scheppler, Katharine Schwedhelm, Nicolette Schuller, Angelina Sharak, Sherry Stanfield-Oakley, Carrie Sopher, Terence Tafatatha, Simbarashe G. Takuva, Chan Tang, An Vandebosch, Edna Viegas, Valentin Voillet, Frank Wegmann, Mo Weijtens, Stephany Wilcox, Anthony Williams, Chenchen Yu, Pei-Chun Yu, Olive Yuan, Xuehan Zhang

**Affiliations:** a Department of Biostatistics, University of Washington, Seattle, WA, USA; b Janssen Vaccines & Prevention BV, Leiden, the Netherlands; c Vaccine and Infectious Disease Division, Fred Hutchinson Cancer Center, Seattle, WA, USA; d Cape Town HVTN Immunology Laboratory, Hutchinson Centre Research Institute of South Africa, Cape Town, South Africa; e Center for Human Systems Immunology, Department of Surgery, Duke University, Durham, NC, USA; f Duke Human Vaccine Institute, Durham, NC, USA; g Public Health Sciences Division, Fred Hutchinson Cancer Center, Seattle, WA, USA; h Department of Biostatistics and Bioinformatics, Rollins School of Public Health, Emory University, Atlanta, GA, USA; i Department of Statistics, University of Washington, Seattle, WA, USA; j Los Alamos National Laboratory, Los Alamos, NM, USA; k New Mexico Consortium, Los Alamos, NM, USA; l Janssen Research & Development BE, Beerse, Belgium; m Division of AIDS, National Institute of Allergy and Infectious Diseases, Bethesda, MD, USA; n Janssen Infectious Diseases BV, Beerse, Belgium; o San Francisco Department of Public Health, San Francisco, CA, USA; p Janssen Research & Development, LLC, Titusville, NJ, USA; q Department of Laboratory Medicine and Pathology, University of Washington, Seattle, WA, USA; r Department of Medicine, University of Washington, Seattle, WA, 98195, USA; s The Aurum Institute, Johannesburg, South Africa; t South African Medical Research Council, Cape Town, South Africa; u Center for Virology & Vaccine Research, Beth Israel Deaconess Medical Center, Boston, MA, USA; v Icahn School of Medicine at Mount Sinai, New York, NY, USA; w Ragon Institute of Massachusetts General Hospital, Massachusetts Institute of Technology, and Harvard, Cambridge, MA, USA; x Department of Integrative Immunobiology, Duke University, Durham, NC, USA

**Keywords:** Ad26.Mos4.HIV vaccine regimen, Binding antibodies, Correlates of risk, Correlates of protection, IgG3 V1V2 antibodies, Maximal signal diversity-weighted average

## Abstract

**Background:**

The HVTN 705 Imbokodo trial of 2636 people without HIV and assigned female sex at birth, conducted in southern Africa, evaluated a heterologous HIV-1 vaccine regimen: mosaic adenovirus 26-based vaccine (Ad26.Mos4.HIV) at Months 0, 3, 6, 12 and alum-adjuvanted clade C gp140 at Months 6, 12. Per-protocol vaccine efficacy (VE) against HIV-1 diagnosis from seven to 24 months was 14.1% (95% CI: −22.0% to 39.5%). Immune correlates analysis was performed for markers selected based on prior evidence in efficacy trials and/or nonhuman primate models.

**Methods:**

Humoral and cellular immune response markers at Month 7 were evaluated as immune correlates of risk and of protection in a breakthrough case–control cohort (n = 52 cases, 246 non-cases). Primary markers were IgG binding to vaccine-strain gp140, IgG3 binding to diverse Env antigens (IgG3 Env breadth), IgG3 binding to diverse V1V2 antigens (IgG3 V1V2 breadth), antibody-dependent phagocytosis against the vaccine-strain gp140, Env-specific CD4+ and CD8+ T-cell responses, and multi-epitope functions.

**Findings:**

No immune markers were statistically significant correlates of risk. IgG3 V1V2 breadth trended toward an inverse association: hazard ratio 0.70 (95% CI: 0.36 to 1.35; p = 0.29) per 10-fold increase and 0.51 (95% CI: 0.21 to 1.24; p = 0.14) in a Cox model with all primary markers. The VE estimate was 11.8% (95% CI: −17.9% to 34.0%) at all IgG3 V1V2 breadth values below 667 weighted geometric mean net MFI; just above this value, the VE estimate sharply increased to 62.6% (95% CI: −17.9% to 89.6%), and further increased to 80.9% (95% CI: −17.9% to 99.5%) at 1471 MFI, the 95th percentile of the marker distribution. Mediation analysis yielded a VE of 35.7% (95% CI: 15.0% to 51.3%) attributable to the vaccine's impact on this marker.

**Interpretation:**

The trend in association of greater IgG3 V1V2 antibody breadth with lower likelihood of HIV acquisition is consistent with the identification of antibodies against V1V2 as immune correlates in three other HIV vaccine efficacy trials and suggests that a greater emphasis should be placed on studying this region in the HIV-1 envelope as a vaccine immunogen.

**Funding:**

10.13039/100000060National Institute of Allergy and Infectious Diseases and Janssen Vaccines & Prevention BV.

**Research in context**

**Evidence before this study**

We searched PubMed on July 27, 2023 for clinical studies on immune correlates of human immunodeficiency virus (HIV)-1 vaccines published from database inception through to the date of search, with no language restrictions. Using the query [“vaccine-induced” and “correlates” and “HIV-1”] retrieved 76 articles, [“HIV-1” and “vaccine efficacy trial” and “immune correlates”] retrieved seven articles, [“correlates of protection” and “HIV vaccine efficacy trial”] retrieved four articles, and [“vaccine efficacy trial” and “correlate” and “HIV-1”] retrieved eight articles. The search yielded 90 unique articles, 13 of which included an immune correlates analysis of an HIV-1 vaccine efficacy trial. Among these were analyses of the RV144 vaccine efficacy trial that identified several inverse correlates of risk of HIV-1 acquisition, including the levels of IgG antibody binding to Env variable regions V1 and V2 (V1V2), Env-specific CD4+ T cell polyfunctionality, and IgG3 binding antibody response and magnitude to Env V1V2. Additionally, each of the following immune parameters inversely correlated with risk (but only when together with low anti-Env IgA antibody binding): IgG avidity, antibody-dependent cellular cytotoxicity, and CD4+ T-cell responses. IgA antibody binding to Env was also identified as a direct correlate of risk of HIV-1 acquisition.

We further searched PubMed on the same date with the same parameters, using the query [“mosaic HIV-1 vaccine” and “protective efficacy”] and retrieved three articles. One of these studies reported on the immunogenicity of multiple vaccine regimens, all of which incorporated the use of bioinformatically optimized mosaic antigens, in preclinical and clinical trials. For one of these regimens, adenovirus serotype 26 (Ad26)-vectored vaccine expressing mosaic HIV-1 Env, Gag, and Pol antigens combined with an aluminum-phosphate adjuvanted Env gp140 protein boost, time-to-acquisition in nonhuman primates (NHPs) after repeated heterologous simian-human immunodeficiency virus (SHIV) challenges was predicted by a model that included binding antibody responses to clade C Env as assessed by ELISA and Env-specific cellular responses to global potential T cell epitope (PTE_g_) peptide pool stimulation and ELISpot, in addition to several other humoral and cellular immune parameters. Binding antibodies to the V1V2 loop also significantly predicted time to acquisition.

To identify additional relevant preclinical literature, we searched PubMed on November 7, 2023 with the same parameters, using the queries [“SIV” and “vaccine” and “challenge” and “correlate”] (255 articles), [“SIV” and “vaccine” and “risk” and “acquisition”] (33 articles), and [“simian immunodeficiency virus” and “vaccine” and “protected” and “challenge"] (173 articles). Among the findings were numerous NHP vaccine studies that incorporated simian immunodeficiency virus (SIV) or SHIV challenge and investigation of immune correlates. We counted seven of these studies that reported a correlation in vaccinated animals between levels/titres of antibodies against one or more V2 antigens (V2 peptides, V1V2-gp70, V1V2 mini protein) and reduced risk of acquisition. Inspection of a review identified in the same search yielded three more such studies. Depending on the regimen tested, additional cellular and/or humoral responses were also identified that correlated with reduced acquisition risk.

**Added value of this study**

This is an immune correlates analysis of a phase 2b efficacy trial (HVTN 705) that tested the efficacy of a heterologous vaccine regimen of Ad26.Mos4.HIV and aluminium-phosphate adjuvanted clade C gp140 protein versus placebo to prevent acquisition of HIV-1 in at-risk women in southern Africa. It provided the opportunity to assess whether the correlates of protection of mosaic Ad26-based HIV-1 vaccines observed in an NHP challenge model directly translated to humans in a real-world exposure context, and to assess whether immune correlates of protection identified in the RV144 pox-protein prime boost regimen were applicable across vaccine platforms and populations. Extensive development and validation of immunoassays were performed, including pilot immunogenicity experiments to identify the most relevant immunogenicity endpoints to move forward to case–control analysis for assessing immune markers as correlates of risk and protection against HIV-1 acquisition.

This study found that the immune correlate markers of T-cell responses and IgG binding to gp140, predicted from the preclinical NHP studies of the Ad26 vaccine, were not significant correlates of HIV-1 risk in HVTN 705. However, this study prespecified testing IgG3 binding antibody breadth to Env V1V2 antigens, with rationale based on prior human and preclinical studies. IgG3 V1V2, which showed low correlations with other immunogenicity readouts, consistently trended with decreased risk, and controlled vaccine efficacy and mediation analyses suggested this immune measure may be a correlate of protection. The correlates results indicated threshold associations wherein attaining high IgG3 V1V2 breadth upwards of the 90th percentile value [above 667 weighted geometric net mean fluorescence intensity (MFI)] marked a precipitous drop in HIV-1 risk and a sharp increase in estimated vaccine efficacy above 50%.

**Implications of all the available evidence**

The lack of evidence for translation of some of the immune correlates of protection in the NHP challenge model to immune correlates in the HVTN 705 trial could potentially be explained by differences between the challenge model versus real-world exposure [e.g., breadth/diversity of challenge vs. circulating viruses, transmission route, concomitant sexually transmitted infections, amount of virus in the challenge dose vs. in a real-world exposure, time and frequency of exposure after vaccination]. The trend of IgG3 binding antibody breadth to V1V2 antigens as an inverse correlate of risk and potential correlate of protection in HVTN 705 is intriguing, and is consistent with V1V2 antibody correlates identified in the RV144, HVTN 505, and HVTN 702 efficacy trials of different vaccine regimens conducted years apart in different study populations. The exploratory analysis of the preclinical study of this vaccine immunogen was consistent in also yielding evidence to support IgG V1V2 as a correlate of protection. It should be noted that there was antigenic matching between the vaccine V1V2 sequences and the NHP challenge virus, something that was considerably less frequent with circulating sequences from the region of the world in which our human study was conducted. The data from HVTN 705 suggest that IgG3 V1V2 binding antibody breadth scores above 667 weighted geometric mean net mean fluorescence intensity (MFI) are required for vaccine efficacy of at least 50%; only a small subgroup of vaccine recipients (9.4%) had breadth scores above this threshold, which may explain the lack of overall efficacy in the trial. Exploration of a way to elicit IgG3 antibodies to V1V2 of substantially improved magnitude, durability, and breadth should be considered.

## Introduction

With the aim of eliciting broad, cross-clade cellular and humoral responses against diverse HIV-1 variants, mosaic HIV-1 vaccines design their inserts to encompass complementary envelope (env), group antigen (gag), and polymerase (pol) sequences from multiple clades that have been computationally predicted to have optimal immune response breadth.^1^ A multivalent human immunodeficiency virus (HIV)-1 vaccine regimen based on a replication-incompetent adenovirus 26 (Ad26) vector for the genetic delivery of mosaic immunogens was developed and shown to elicit such responses.^2^, ^3^, ^4^ Ad vectors expressing mosaic Env, Gag, and Pol antigens boosted with modified vaccinia Ankara (MVA) or Ad vectors expressing the same immunogens provided 90% and 87% reduction, respectively, in per-exposure acquisition risk against six heterologous, neutralization-resistant rectal simian-HIV (SHIV; SHIV-SF162P3) challenges in rhesus monkeys.^5^ Immune responses correlating with the number of challenges before each animal acquired SHIV included binding antibodies to the vaccine-matched Env immunogen, neutralizing antibodies against SF162 (neutralization-sensitive, related to SHIV-SF162P3), and antibody-dependent cellular phagocytosis (ADCP) score (Barouch et al.^5^).

In a subsequent study, the addition of aluminium-phosphate adjuvanted recombinant clade C glycoprotein (gp) 140 to an Ad26-based mosaic vaccine expressing Env, Gag, and Pol antigens (Ad26.Mos.HIV) provided a 94% reduction in per-exposure acquisition risk and 66% complete protection to rhesus monkeys against six SHIV-SF162P3 challenges.^6^ An immune correlates model including immunoglobulin G (IgG) antibodies binding to the vaccine-matched gp140 antigen and potential T-cell epitope (PTE) Env interferon (IFN)-γ enzyme-linked immunosorbent spot (ELISpot) responses best predicted time-to-infection (p = 0.003 and p = 0.001, respectively), with binding antibodies to variable regions V1 and V2 (A244 gp70-scaffolded V1V2 ELISA), the scaffold with high correlation in RV144, also predictive (p = 0.007) (Supplementary Table S1 in Ref.^6^). Similar immune responses were induced in clinical trial participants,^6^ with high levels of anti-gp140 binding and ADCP-mediating antibodies and T-cell responses, and relatively low levels of V1V2 binding antibodies. The geometric mean titre (GMT) to A244 gp70-scaffolded V1V2 antibodies in humans exceeded those of the NHP, albeit the breadth of these responses to other prototype V1V2 antigens was low, reflecting the wide variability of this area of the HIV-1 genome. The multivalent^3^ vaccine regimen was tested versus placebo in the Imbokodo phase 2b efficacy trial (HVTN 705/HPX2008; ClinicalTrials.gov Identifier: NCT03060629), which showed a lack of statistically significant vaccine efficacy.^7^

This study was designed to address secondary objective 6 of the Imbokodo trial, “To evaluate immunogenicity and immune response biomarkers among vaccine recipients after the third vaccination as correlates of risk of subsequent HIV acquisition and correlates of vaccine efficacy, if deemed applicable.” We analysed a set of primary immune markers measured at Month 7 (4 weeks post-third vaccination) as immune correlates of risk and protection against HIV-1 acquisition through Month 24. A total of 20 immune markers were assessed (7 primary, 13 exploratory), including total IgG and IgG3 binding antibody levels to gp120, gp140 and V1V2 antigen panels; ADCP to vaccine-matched gp140s; antibody-dependent cellular cytotoxicity (ADCC) against Env CAP8, CH58, and WITO strains; and Env-specific CD4+ and CD8+ T-cells expressing IFN-g and/or interleukin (IL)-2.

## Methods

### Trial design and participants

In sub-Saharan Africa, women and girls (of all ages) bear the largest burden of new HIV acquisitions globally^8^ and are thus one of the populations most in need of effective HIV prevention interventions. The Imbokodo trial (NCT03060629) was a phase 2b, multicentre, randomised, double-blind, placebo-controlled study conducted in Malawi, Mozambique, South Africa, Zambia, and Zimbabwe. Inclusion criteria included having been assigned female sex at birth, being sexually active (defined as having had sexual intercourse with a male partner at least twice in the past 30 days prior to screening), being at increased risk of acquiring HIV-1, and being both HIV-1-negative and HIV-2-negative. Full inclusion and exclusion criteria, as well as further details on study design and oversight, are provided with Gray et al.^7^

### Ethics

The Imbokodo trial adhered to the principles of the Declaration of Helsinki and Good Clinical Practice guidelines. The protocol, protocol amendments, and other relevant documents were approved by institutional review boards, ethics committees, and the applicable regulatory entities. All participants provided written informed consent. An independent data and safety monitoring board periodically reviewed the safety and efficacy data. The Imbokodo trial is registered with ClinicalTrials.gov, NCT03060629.

For the Imbokodo trial, Janssen and the HVTN observed Good Participatory Practice (GPP) Guidelines, which were developed by AVAC and UNAIDS. Clinical Research Sites have established long term relationships with community stakeholders. These relationships facilitate community stakeholder engagement throughout the trial from participation in protocol development though results dissemination. The trial adhered to principles of equity and inclusivity in its conduct by providing a comprehensive HIV-1 prevention package to participants, educating community members, and by recruiting participants from a population with greater likelihood of HIV acquisition (young women in southern Africa).

### Study vaccinations and follow-up

Participants were centrally randomised (1:1) to receive vaccine or placebo in stratified permuted blocks via an interactive web response system. Participants in the treatment arm received Ad26.Mos4.HIV at Months 0, 3, 6, and 12, as well as aluminium-phosphate adjuvanted clade C gp140 (C97ZA) at Months 6 and 12. Placebo recipients received saline at Months 0, 3, 6, and 12. All syringes were covered with an overlay to preserve blinding. Participants were assessed for HIV-1/2 acquisition at Months 0, 3, 6, 7, 9, and every three months thereafter, for 2 or more years after enrolment. The last participant's final visit occurred on February 2, 2022. See Gray et al.^7^ for details on the vaccine regimen and the HIV diagnostic procedures.

### Study cohort, sample design, and outcome

Assays were selected for immune correlates analysis based on a pilot immunogenicity study (see Appendix p 12, Table S12, and Figures S3–S13). Immune correlates analyses were conducted in the per-protocol (PP) cohort, defined as the set of randomised participants who were living without HIV four weeks post-third vaccination visit (negative HIV-1 test result at Month 7), received all planned vaccinations at the first three vaccination visits within the protocol-specified windows, and had no other major protocol deviations that could impact VE. The PP cohort is the same as assessed in the primary report,^7^ with a data cut-off date of May 28, 2021 for correlates analyses.

A two-phase case–control sampling design was used, in which the immune markers were measured at Month 7 (also at Month 0 for most markers) in all PP vaccine recipient cases and in a stratified random sample of PP vaccine recipient non-cases, where non-cases must reach the Month 27 visit with a negative HIV-1 test result. Within each demographic stratum defined by randomisation arm cross-classified with the six substrata as defined by body mass index [BMI; BMI<25, 25 ≤ BMI<30, BMI≥30 kg/m^2^] × (South Africa, outside of South Africa), a without-replacement sample of non-cases was randomly sampled, with sample size for each vaccine arm substrata equal to five times the number of vaccine recipient cases in the demographic stratum. The case–control sampling plan is in the Appendix (p 2).

Immune correlates were assessed for the primary efficacy endpoint defined as a confirmed HIV-1 diagnosis between the Month 7 and Month 24 visits. In-study HIV testing was performed according to the HVTN HIV diagnostic testing algorithms, provided in section 10.3 of the HVTN 705/HPX2008 study protocol (located in the Appendix of Gray et al.^7^).

### Immune response marker Selection and validation

The first six primary markers were IgG gp140 C97ZA, IgG3 V1V2 breadth, IgG3 gp120+gp140 breadth, ADCP gp140 C97ZA, Env-specific IFN-g and/or IL-2 expressed by CD4+ T cells, and Env-specific IFN-g and/or IL-2 expressed by CD8+ T cells. The two breadth scores were maximal signal diversity-weighted (MDW)^9^ averages of antigen-specific readouts for eight V1V2 antigens (Table S2) and for 13 Env antigens (five gp120, eight gp140) (Table S3). The Binding Antibody Multiplex Assay (BAMA) antigens were selected for this study through an analysis of the vaccine immunogen sequence and available sequence reagents to maximally capture breadth in Southern Africa (Appendix p 3, Figure S1). The two T-cell markers are percent of CD4+ (or CD8+) T cells expressing IFN-g and/or IL-2 upon stimulation with any Env peptide pool. CD4+ and CD8+ T-cell response magnitudes to any Env were defined as the maximum over all Env peptide pools tested. The seventh primary marker–“multi-epitope functions”–is a maximum diversity-weighted average of the first six primary markers and of five additional markers: ADCP gp140 Mos1; IgG V1V2 breadth (same antigens as for the IgG3 primary marker); and ADCC partial area under the curve (AUC) against each strain CAP8, CH58, WITO. Assay details are in the Appendix (pp 5–10, Tables S4–S11).

The first four primary markers were prespecified in the Version 1.0 Statistical Analysis Plan for Correlates (hereafter, “SAP”), which also specified potential T-cell epitope (PTE) Env IFN-γ ELISpot as a primary marker. ELISpot response rates and magnitudes for case–control vaccine recipients were both substantially lower than observed previously with this vaccine regimen^3^^,^^4^^,^^6^ and in the HVTN 705 pilot study (59.6% vs. 82.4% positive response frequency in vaccine non-cases, respectively; median readout 74 vs. 308, Figure S15), differences that were not observed for the other immune markers. This observation suggested assay quality problems with the ELISpot marker in the case–control dataset, and therefore after careful consideration it was removed from the correlates analyses. However, it was retained for the descriptive analysis in comparison with the pilot data to document the significantly lower responses (Figure S15, Table S13). The v1.0 SAP specified CD4+ and CD8+ T cells expressing IFN-γ and/IL-2 as measured by ICS as elements of the multi-epitope function primary marker. Given the importance of including T cell markers in multivariable/joint marker correlates analyses, these two markers were added post hoc to the multivariable correlates model of primary markers.

The role of the conformation of the V1V2 region in antibody recognition, specifically the β-sheet conformation (V2i-binding) and the α-helical conformation (V2p-binding),^10^, ^11^, ^12^ was also examined post-hoc. The exploratory IgG3 V2i and V2p breadth score markers are detailed in the Supplementary Methods. All other exploratory markers are described in the SAP.

### Statistics

All correlates analyses were prespecified in the SAP, with the exception of the previously noted updates regarding the T-cell markers. The SAP provides complete details of version history. All analyses were adjusted for the following baseline variables: geographical region, BMI, age at enrolment, and baseline behavioural risk score (built by machine learning analysis of the placebo arm, see SAP). Correlate of risk analyses were conducted in PP vaccine recipients. These analyses study associations between Month 7 markers and HIV-1 diagnosis and do not assess causation, though by adjusting for baseline factors they attempt to isolate the most meaningful association. In contrast, the correlate of protection analyses allow for a specific causal interpretation, include the baseline variables as potential confounders of the marker-outcome relationship, and require additional strong assumptions (most notably no unmeasured confounders) to conclude causality.

Each Month 7 marker was assessed individually as a correlate of risk, using inverse probability sampling (IPS)-weighted Cox regression fit using the ‘survey’ R package^13^ to provide point and 95% confidence interval (CI) estimation of the covariate-adjusted hazard ratio (HR) of HIV-1 diagnosis across marker tertiles, or per 10-fold (or standard deviation-) increase in the marker level. Wald-based p values were used to test for an association of each antibody marker with HIV-1 diagnosis. These Cox models were also used to estimate marker-conditional cumulative incidence of HIV-1 diagnosis with bootstrap 95% CIs. Nonparametric dose–response regression^14^ was also used to estimate marker-conditional cumulative incidence of HIV-1 diagnosis, with influence function-based 95% CIs. Point and 95% CI estimates of marker threshold-conditional cumulative incidence of HIV-1 diagnosis were calculated using nonparametric targeted minimum loss-based threshold regression.^15^

A multivariable Cox model was fit including the seven primary markers. Point and 95% CI estimates of the seven HRs are reported, along with a generalised Wald test of the complete null hypothesis that none of the markers correlate with HIV-1 diagnosis.

Point and 95% CI estimates of controlled VE by each Month 7 immune marker were obtained by a causal inference approach using Cox proportional hazards estimation and nonparametric monotone dose–response estimation,^16^ as implemented in the ‘vaccine’ R package,^17^ where the latter approach has advantage of allowing an arbitrary nondecreasing relationship between the marker and VE.

Each Month 7 immune marker was assessed as a mediator of VE using the nonparametric method described by Benkeser et al.^18^ Based on the prespecified requirement of only including markers with at least 10% of vaccine recipients having marker value equal to the value in placebo recipients (negative/below assay lower limit), three primary markers qualified: IgG3 V1V2 breadth and the two T-cell markers. The mediation and controlled VE methods have been applied in a similar way to COVID-19 vaccine efficacy trials.^19^, ^20^, ^21^, ^22^, ^23^

In all correlates analyses, the time to HIV-1 diagnosis is right-censored at the minimum of the time of loss-to-follow-up and the right edge of the longest Month 24 visit window (25.91 months). All analyses were implemented in R version 4.0.3. All p-values are two-sided. For the correlates of risk and controlled VE analyses separately, multiplicity adjustment is performed on the set of p-values for the hypothesis tests of individual primary markers being correlated with HIV-1 diagnosis (Cox models) and modifying controlled VE (nonparametric method), respectively. Westfall-Young^24^ family-wise error rate (FWER)-adjusted p-values are computed. Multiplicity adjustment is restricted to the seven primary markers.

### Role of funders

This study was co-funded through a public-private partnership that included the National Institute of Allergy and Infectious Diseases (NIAID) and its Division of AIDS (DAIDS), both of the National Institutes of Health (NIH); the HIV Vaccine Trials Network (HVTN) (Fred Hutchinson Cancer Center); the Bill & Melinda Gates Foundation; Janssen Vaccines and Prevention BV (Janssen); US Army Medical Materiel Development Activity; and the Ragone Institute of the Massachusetts Institute of Technology, Massachusetts General Hospital, and Harvard University; and the Department of Medicine, Icahn School of Medicine at Mount Sinai. The trial was co-designed by the NIAID-supported HVTN and conducted through the HVTN. All co-funding parties with the exception of the Department of Medicine, Icahn School of Medicine at Mount Sinai participated in trial design. All co-funding parties with the exception of the Bill & Melinda Gates Foundation participated in the collection, analysis, and interpretation of data; writing the paper; and decision to submit for publication.

For the Imbokodo (HVTN 705/HPX2008) efficacy trial (Gray et al.^7^), the HIV Vaccine Trials Network and Janssen, in collaboration with the Bill & Melinda Gates Foundation and Ragone Institute, had roles in the study design. The HIV Vaccine Trials Network, Janssen, and the Ragone Institute had roles in the collection, analysis, and interpretation of data; the writing of the paper; and the decision to submit for publication.

## Results

### Study population and case-control set selected for immune marker Measurements

From November 3, 2017 to June 30, 2019, 2636 participants were enrolled at 23 sites in five southern African countries. A flowchart of sampling of PP recipients into the case–control sampling design is shown in Figure S2. Fifty-two PP vaccine recipients who acquired HIV-1 on study (cases) and 246 PP vaccine recipients who remained without HIV throughout the 24 month follow-up (non-cases) had Month 7 marker values and were included in correlates analyses; a small number of exploratory marker values were missing (<1% of total values) and were imputed using predictive mean matching, enabling inclusion of the same 52 cases and 246 non-cases in all correlates analyses (see SAP). The low number of missing values implies this approach does not meaningfully influence results.

### Pilot immunogenicity study and antigen down-Selection

Pilot immunogenicity experiments were performed to identify the most relevant immunological readouts and time points to move forward to case–control immunogenicity and immune correlates analysis. Results from the pilot assays performed on Month 0, 7, 13, and 24 samples are provided in Figures S3–S12. Particular antigens were down-selected for the case–control analysis. For ICS, six of the 12 peptide pools were selected based on pools with sufficient response rates/magnitudes and low response correlations with each other (Figure S13); for ADCC, one heterologous strain (SUMA) was not tested due to insufficient response frequency. Antigen panels for BAMA^25^ and for ADCC^26^ were previously optimised for maximising coverage in mapping antibody responses while also minimising redundancy.

### Intercorrelations and distributions of immune markers

The intercorrelations of the seven primary markers among vaccinees in the case–control subset are shown in Figure S14. We observed high correlation of the IgG gp140 C97ZA and ADCP gp140 C97ZA markers (rho = 0.92), moderate correlations of the multi-epitope functions marker with the IgG gp140 C97ZA, ADCP gp140 C97ZA, and CD4+ IFN-g and/or IL-2 Env markers (rho = 0.72, 0.71, and 0.64, respectively); moderate correlation of the IgG gp140 C97ZA and CD4+ IFN-g and/or IL-2 Env markers (rho = 0.61); and weak to moderate correlations between all other marker pairs. IgG3 V1V2 breadth showed the weakest correlation with other immune measures (rho = 0.098 to 0.45, Figure S14), indicating that this marker represented the most unique immune space.

**Fig. 1:**
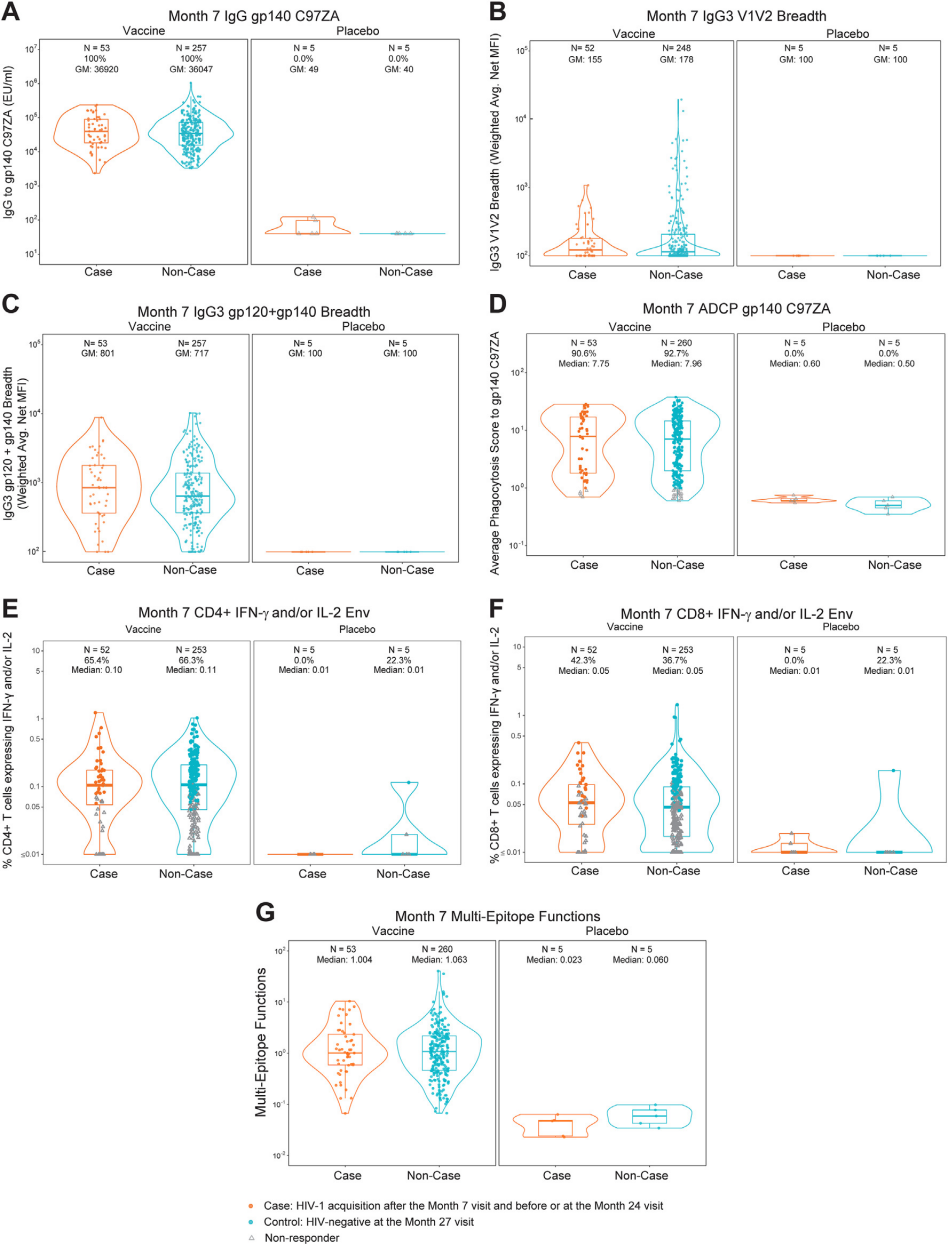
Distribution of the seven primary immune markers (measured at Month 7) in per-protocol participants, stratified by randomisation arm (vaccine vs. placebo) and case/non-case outcome status. Markers include (A) IgG gp140 C97ZA, (B) IgG3 V1V2 breadth, (C) IgG3 gp120+gp140 breadth, (D) ADCP gp140 C97ZA, (E) CD4+ IFN-g and/or IL-2 Env, (F) CD8+ IFN-g and/or IL-2 Env, and (G) Multi-epitope functions: the maximum diversity-weighted average of the first six primary markers and of ADCP gp140 Mos1, IgG V1V2 breadth, and ADCC partial area-under-the-curve against each strain CAP8, CH58, WITO. Each violin plot contains a boxplot showing the estimated 25th, 50th, and 75th percentiles of the marker distribution, as well as a (rotated) kernel density estimate of the marker probability density function. Boxplots are based on observed response magnitudes among responders (filled coloured circles) and nonresponders (empty grey triangles). Panels show the actual numbers of participants with available assay data above the response rates (if relevant) and median or geometric mean (GM) values. The response rates and median or GM values accounted for inverse-probability-of-sampling weights and thus are for the population of eligible participants from which the case/control study cohort was randomly sampled. ADCC, antibody-dependent cellular cytotoxicity; ADCP, antibody-dependent cellular phagocytosis; Env, envelope; GM, geometric mean; gp, glycoprotein; HIV, human immunodeficiency virus; IFN, interferon; IgG, immunoglobulin G; IL, interleukin; MFI, mean fluorescence intensity; V1V2, variable regions V1 and V2.

**Fig. 2:**
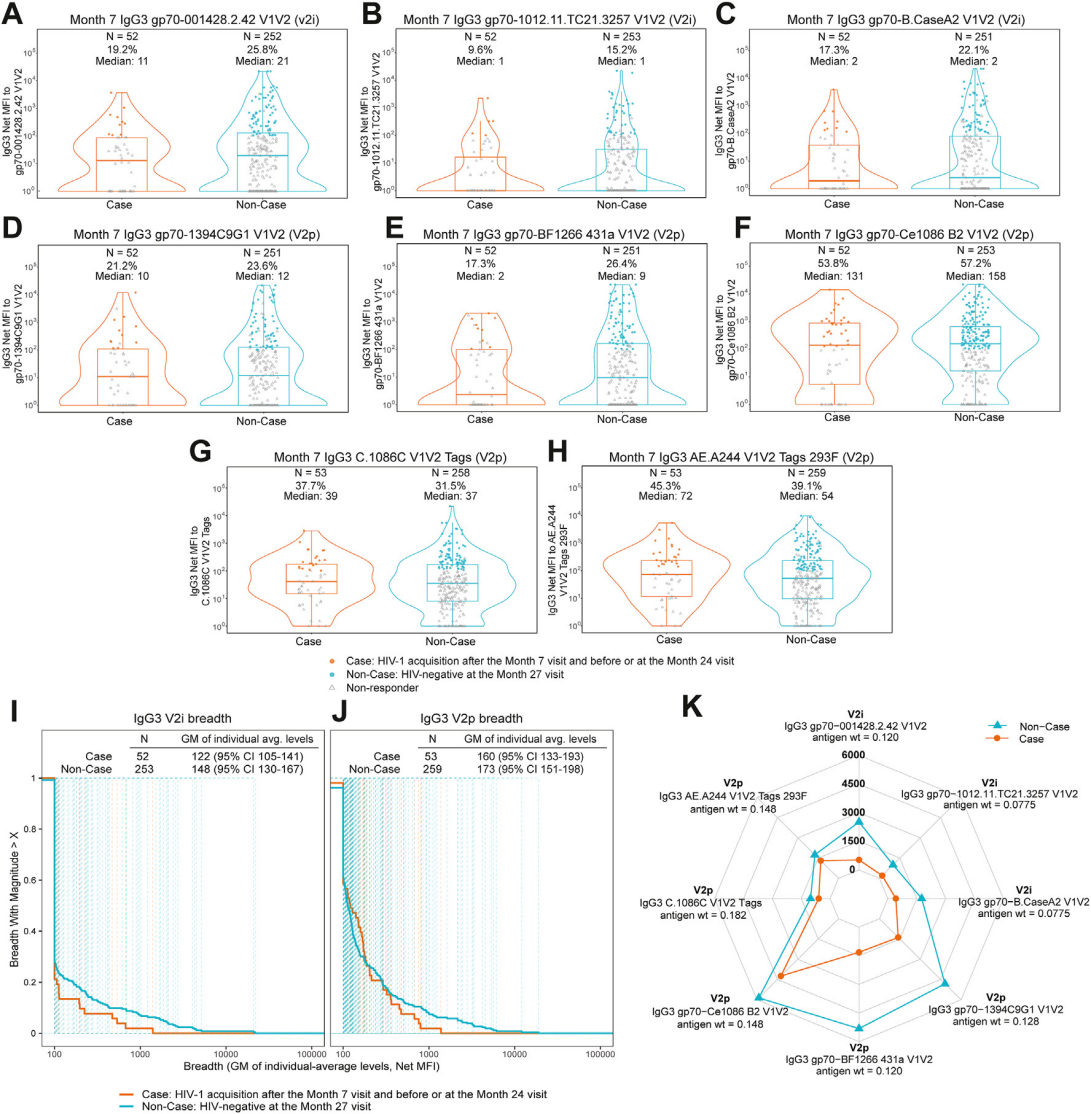
(A–H) Reactivity in per-protocol vaccine recipients of IgG3 antibodies to the eight exploratory individual V1V2 antigens (measured at Month 7) that comprised the IgG3 V1V2 breadth primary marker, stratified by case/non-case outcome status; (I, J) magnitude-breadth curves of Month 7 responses, stratified by case/non-case outcome status, for breadth of antibodies reactive with antigens with preferential reactivity to V2i (I) or V2p (J) antigens; and (K) IgG3 95th percentile levels (net MFI) against each V1V2 antigen. Markers include (A) IgG3 gp70–001428.2.42 V1V2, (B) IgG3 gp70–1012.11.TC21.3257 V1V2 (C), IgG3 gp70-B.CaseA2 V1V2, (D) IgG3 gp70-1394C9G1 V1V2, (E) IgG3 gp70-BF1266 431a V1V2, (F) IgG3 gp70-Ce1086 B2 V1V2, (G) IgG3 C.1086C V1V2 Tags, and (H) IgG3AE.A244 V1V2 Tags 293F and are ordered from highest to lowest mean AUC difference V2p versus V2i mAbs (Figure S20). Each violin plot contains a boxplot showing the estimated 25th, 50th, and 75th percentiles of the marker distribution, as well as a (rotated) kernel density estimate of the marker probability density function. Boxplots are based on observed response magnitudes among responders (filled coloured circles) and nonresponders (empty grey triangles). Panels show the actual numbers of participants with available assay data above the response rates (if relevant) and median or geometric mean (GM) values. The response rates and median or GM values accounted for inverse-probability-of-sampling weights and thus are for the population of eligible participants from which the case/control study cohort was randomly sampled. LLoQ: lower limit of quantitation. Magnitude-breadth curves are shown for (I) the antigens with greater reactivity against V2i monoclonal antibodies [(A), (B), (C)] and (J) the antigens with a relatively lower reactivity for V2i monoclonal antibodies and higher reactivity with V2p monoclonal antibodies [(D)–(H)].^12^ (K) includes the maximal-signal diversity weights assigned to the 8 antigens in the IgG3 V1V2 breadth primary marker. AUC, area under the curve; CI, confidence interval; GM, geometric mean; gp, glycoprotein; HIV, human immunodeficiency virus; IgG, immunoglobulin G; MFI, mean fluorescence intensity; V1V2, variable regions V1 and V2; V2i, β-sheet conformation; V2p, α-helical conformation.

Fig. 1 shows the distributions of the primary markers at Month 7. Table 1 reports sample sizes, geometric mean values, and response rates (when available) in vaccine recipients by case/non-case outcome status, as well as case/non-case comparisons. Almost all immune response markers for placebo recipients indicated negative responses, verifying appropriate specificities of the assays. Positive response rates for vaccine recipient non-cases were 100% for ELISA, 92.7% for ADCP, 66.3% for CD4+ IFN-g and/or IL-2 Env, and 36.7% for CD8+ IFN-g and/or IL-2 Env. Response rates and geometric means for vaccine recipient cases versus non-cases were similar; V1V2 IgG3 responses were less frequently elicited compared to the other immune responses (Fig. 2A–H).

**Table 1:** Sample sizes, positive response rates, and geometric mean values in per-protocol vaccine recipients for each of the seven primary markers at Month 7, stratified by case/non-case outcome status.

Month 7 marker	Cases (N = 52 with marker data)		Non-Cases (N = 246 with marker Data)		Comparison	
	% Positive Response^a^ (95% CI)	Geometric mean (GM)^a^ (95% CI)	% Positive response^a^ (95% CI)	Geometric mean^a^ (95% CI)	Response rate difference [non-cases—cases (95% CI)]	Ratio of GM [non-cases/cases (95% CI)]
IgG gp140 C97ZA	100.0% (100.0%, 100.0%)	36,515 (27,411, 48,643)	100.0% (100.0%, 100.0%)	36,202 (31,613, 41,457)	0% (0%, 0%)	0.99 (0.72, 1.36)
IgG3 V1V2 breadth	N/A	155 (132, 182)	N/A	179 (158, 203)	N/A	1.15 (0.94, 1.41)
IgG3 gp120+gp140 breadth	N/A	779 (585, 1039)	N/A	708 (616, 812)	N/A	0.91 (0.66, 1.25)
ADCP gp140 C97ZA	90.6% (78.9%, 96.1%)	5.50 (3.99, 7.60)	92.7% (88.9%, 95.3%)	6.04 (5.23, 6.99)	2% (−5%, 14%)	1.10 (0.77, 1.56)
CD4+ IFN-g and/or IL-2 Env	65.4% (51.5%, 77.1%)	0.08 (0.06, 0.12)	66.3% (59.9%, 72.1%)	0.09 (0.08, 0.10)	1% (−12%, 16%)	1.05 (0.73, 1.50)
CD8+ IFN-g and/or IL-2 Env	42.3% (29.4%, 56.4%)	0.05 (0.04, 0.06)	36.7% (30.5%, 43.3%)	0.04 (0.04, 0.05)	−6% (−21%, 9%)	0.91 (0.67, 1.23)
Multi-epitope functions	N/A	1.07 (0.79, 1.45)	N/A	1.03 (0.89, 1.19)	N/A	0.96 (0.69, 1.35)

ADCP, antibody-dependent cellular phagocytosis; CI, confidence interval; Env, envelope; GM, geometric mean; gp, glycoprotein; IFN, interferon; IgG, immunoglobulin G; IL, interleukin; N/A, not applicable; V1V2, variable regions V1 and V2.

a Inverse probability of sampling weights was applied such that estimates are for the population of eligible participants from which the case/control study cohort was selected.

**Fig. 3:**
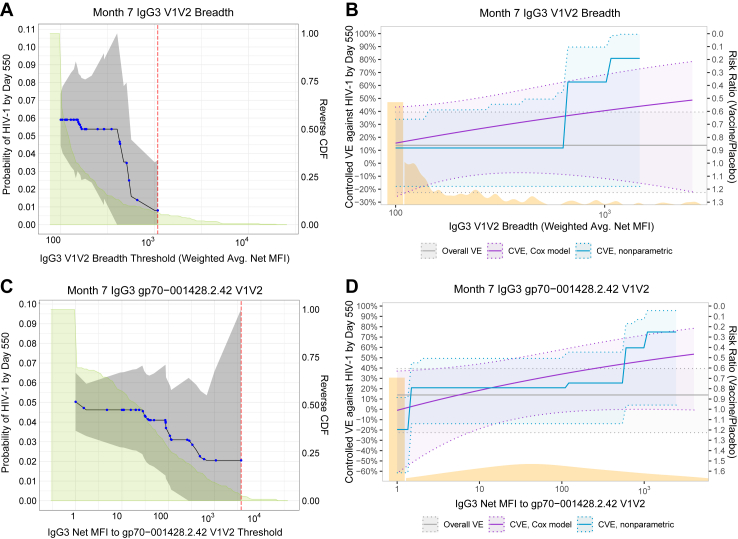
Analyses of Month 7 IgG3 V1V2 breadth score and Month 7 IgG3 gp70-001428.2.42 V1V2 as correlates of risk and correlates of protection. (A and C) Covariate-adjusted cumulative incidence of HIV-1 by Day 550 (post Month 7) for subgroups of per-protocol vaccine recipients with (A) an IgG3 V1V2 breadth score or (C) reactivity of IgG3 antibodies to the gp70-001428.2.42 V1V2 antigen above a given threshold value, estimated using nonparametric threshold regression. The blue dots represent point estimates at each new HIV-1 acquisition endpoint, and the black lines represent a linear interpolation of these points. The grey shaded area represents pointwise 95% confidence bands, and the upper boundary of the green shaded area represents the estimated reverse cumulative distribution function (CDF) of the marker. The estimates and CIs assume a nonincreasing threshold-response function. The red vertical dashed line is the marker threshold corresponding to the largest observed HIV-1 event time (within the time frame of interest). (B) Controlled vaccine efficacy (CVE) as a function of IgG3 V1V2 breadth score, controlling for covariates. Estimates are computed separately using a Cox proportional hazards model (solid purple line) and a nonparametric method that assumes that the CVE curve is nondecreasing (solid blue line). Corresponding 95% pointwise confidence bands are shown in shaded blue/purple, with dotted lines representing the corresponding lower and upper limits. The distribution of the marker, estimated using KDE, is plotted in orange with the addition of a rectangle at the lower limit representing a point mass at the marker minimum value. The solid grey line represents estimated overall VE, and the dotted grey lines represent the corresponding 95% CI limits. The Cox model estimates are cut off at the 97.5th quantile of the marker distribution and the nonparametric estimates are cut off at the 95th quantile. (C) and (D) are analogous to (A) and (B), respectively, but for the Month 7 IgG3 gp70–001428.2.42 V1V2 marker. CI, confidence interval; CVE, controlled vaccine efficacy; gp, glycoprotein; HIV, human immunodeficiency virus; IgG, immunoglobulin G; KDE, kernel density estimation; LLOQ, lower limit of quantitation; MFI, mean fluorescence intensity; V1V2, variable regions V1 and V2; VE, vaccine efficacy. Baseline covariates adjusted for: region (RSA versus Non-RSA), age, body mass index, baseline risk score.

To further understand the specificity of the IgG3 V1V2 response, we next evaluated the reactivity of the IgG3 antibodies induced by the vaccine with the eight exploratory V1V2 antigens comprising the IgG3 V1V2 breadth primary marker. Fig. 2 shows the distribution of these eight exploratory markers by case/non-case outcome status, and Figure S20 shows the reactivity of these 8 antigens with V2i and V2p monoclonal antibodies. The positive response rates of non-cases were 25.8%, 15.2%, 22.1% against the V2i-preferred antigens (Fig. 2A–C), and somewhat higher, ranging between 23.6% to 57.2%, against the V2p-preferred antigens (average 35.6%) (Fig. 2D–H). Based on magnitude-breadth curves for IgG3 V2i and V2p responses (Fig. 2I and J), the geometric mean of individual-average levels to the antigen panel was slightly smaller in cases than in non-cases [for IgG3 V2i breadth: 122 (95% CI 105 to 141) versus 148 (130–167), respectively; for IgG3 V2p breadth: 160 (133–193) versus 173 (151–198), respectively]. Fig. 2K shows 95th percentiles of IgG3 V1V2 responses to each of the eight antigens, indicating considerably larger levels for non-cases vs. cases, aiding the interpretation of the correlate of protection trend finding presented in Fig. 3 below. The distributions of the additional 5 exploratory markers that contributed to the multi-epitope functions primary marker are in Figures S18 and S19. Of note, a V1V2 SF162 antigen, similar to the challenge virus strain used in the preclinical development of this vaccine candidate^6^ bound both V2p and V2i monoclonal antibodies (unpublished), consistent with a potential role for these antibodies in the efficacy observed in this model.

### Correlates of risk

When assessed in univariate models, none of the primary markers significantly associated with acquisition (Table 2A). IgG3 V1V2 breadth trended toward being an inverse correlate of risk (HR: 0.70 per 10-fold marker increase, 95% CI 0.36 to 1.35; p = 0.29). When assessed in a multivariable marker model that included the seven primary markers as quantitative markers, IgG3 V1V2 breadth also trended toward being an inverse correlate of risk (HR = 0.51 per 10-fold marker increase; 95% CI: 0.21 to 1.24; p = 0.14) (Table 2B). In exploratory investigations of the individual antigens comprising the V1V2 panel, all gp70-scaffolded V1V2 antigens had HRs below 1, whereas the V1V2 tags-construct antigens had HRs above 1; moreover, the three V1V2 antigens with the smallest HRs were also the three antigens with greater recognition by V2i-monoclonal antibodies than by V2p-monoclonal antibodies (Table S14).

**Table 2:** A) For each of the seven primary markers at Month 7, hazard ratio of HIV-1 acquisition from 7 to 24 months in the per-protocol vaccine group per 10-fold marker increase, estimated using univariate Cox models.

A). Univariate models				
Marker	HR per 10-fold increase	p-value (2-sided)	Q-value^a^	FWER-adj p-value
	Pt. Est. (95% CI)			
IgG gp140 C97ZA	1.27 (0.65, 2.46)	0.48	0.80	0.99
IgG3 V1V2 breadth	0.70 (0.36, 1.35)	0.29	0.80	0.90
IgG3 gp120+gp140 breadth	1.21 (0.62, 2.36)	0.57	0.80	0.99
ADCP gp140 C97ZA	1.02 (0.54, 1.92)	0.96	0.99	1.0
CD4+ IFN-g and/or IL-2 Env	1.00 (0.56, 1.76)	0.99	0.99	1.0
CD8+ IFN-g and/or IL-2 Env	1.18 (0.64, 2.19)	0.59	0.80	0.99
Multi-epitope functions	1.16 (0.65, 2.06)	0.61	0.80	0.99

B). Multivariate model				
Marker	HR per 10-fold increase	p-value		
	Pt. Est. (95% CI)			
IgG gp140 C97ZA	2.63 (0.64, 10.77)	0.18		
IgG3 V1V2 breadth	0.51 (0.21, 1.24)	0.14		
IgG3 gp120+gp140 breadth	1.28 (0.49, 3.34)	0.62		
ADCP gp140 C97ZA	0.39 (0.11, 1.37)	0.14		
CD4+ IFN-g and/or IL-2 Env	0.79 (0.38, 1.62)	0.51		
CD8+ IFN-g and/or IL-2 Env	1.06 (0.47, 2.41)	0.89		
Multi-epitope functions	1.49 (0.35, 6.30)	0.58		
Generalised Wald Test		0.50		

B) Hazard ratio of HIV-1 diagnosis in the per-protocol vaccine group per 10-fold marker magnitude increase, estimated using a multivariate Cox model.

Baseline covariates adjusted for: region (RSA versus Non-RSA), age, body mass index, baseline risk score.

ADCP, antibody-dependent cellular phagocytosis; CI, confidence interval; Env, envelope; FWER, family-wise error rate; gp, glycoprotein; HIV, human immunodeficiency virus; HR, hazard ratio; IFN, interferon; IgG, immunoglobulin G; IL, interleukin; Pt est, point estimate; RSA, Republic of South Africa; V1V2, variable regions V1 and V2.

a q-value and FWER (family-wide error rate) are computed over the set of p values both for quantitative markers and categorical markers using the Westfall and Young permutation method (10,000 replicates).

For each Month 7 immune marker, nonparametric threshold regression analyses estimated the cumulative incidence of HIV-1 diagnosis by Month 24 for subgroups of PP vaccine recipients with marker level above a given threshold value. Fig. 3A shows the results for IgG3 V1V2 breadth. The cumulative incidence estimates were 0.059 (95% CI: 0.044 to 0.074) for the whole cohort, and 0.035 (95% CI: 0.0 to 0.080) and 0.008 (95% CI: 0.0 to 0.035) at IgG3 V1V2 breadth thresholds of 500 and 1000 weighted geometric mean net mean fluorescence intensity (MFI), respectively. Fig. 3C shows results of the same analysis for IgG3 against the V1V2 antigen in the breadth panel with the greatest dynamic range of vaccine elicited antibodies, gp70–001428.2.42 (hereafter, “1428-V1V2”). Antigenicity characterisation of the V1V2 reagents revealed a preference for binding by V2i antibodies over V2p antibodies for 1428-V1V2 (Figure S20). The cumulative incidence estimates were 0.041 (95% CI: 0.017 to 0.065) and 0.022 (95% CI: 0.0 to 0.056) at IgG3 1428-V1V2 thresholds of 49 and 500 net MFI, respectively.

Results of the same analyses for the other six primary markers and for the other individual antigens comprising the V1V2 panel are in Figures S21 and S22.

### Correlates of protection: controlled VE

VE did not significantly increase with Month 7 immune response marker value for any of the primary markers. There was a trend for VE to increase with Month 7 IgG3 V1V2 breadth (p = 0.24) (Fig. 3B). We interpret the results of the nonparametric analysis (blue curves) given the evidence of better model fit: the VE estimate was 11.8% (95% CI: −17.9% to 34.0%) at all marker values below 667 weighted geometric mean net MFI; just above this value, the VE estimate sharply increased to 62.6% (95% CI: −17.9% to 89.6%), and the VE estimate further increased to 80.9% (95% CI: −17.9% to 99.5%) at 1471 MFI, the 95th percentile of the marker distribution. The magnitude of these increases, as well as the marker values at which the increases occur, should be interpreted with caution given the wide confidence intervals. A sensitivity analysis of four different Month 7 exploratory IgG3 V1V2 breadth markers (Appendix p 4) showed similar results (Figure S23).

Fig. 3D shows the same analysis for the IgG3 1428-V1V2 marker, which, based on the nonparametric analysis, shows increasing VE with an estimate of −22.0% (95% CI: −71.7% to 27.7%) at marker values below 1 and an estimate of 74.8% (95% CI: 1.6% to 97.2%) at the 95th percentile marker value 2438 MFI. This MFI corresponds to a median concentration of 3.68 μg/ml CH58 IgG3 equivalent concentration. Results of the same analysis for the other six primary markers and for the other individual antigens comprising the V1V2 panel are in Figures S24 and S25.

Baseline seropositivity of the Ad26 viral vector is not expected to have impacted this trend to a correlate, as Month 7 IgG3 V1V2 breadth responses were similar between baseline Ad26 seronegative versus seropositive per-protocol vaccine recipients (p = 0.67) (Table S15).

### Correlates of protection: mediation

The Benkeser et al. mediation method was applied to assess all qualifying markers as a mediator of VE. This method decomposes the overall VE into two components, estimating VE mediated through the marker (“Marker-Mediated VE”) and through all means other than through the marker (“Non-Marker Mediated VE”). For IgG3 V1V2 breadth, the Non-Marker Mediated VE estimate was −35.0% (95% CI: −102% to 9.9%) and the Marker-Mediated VE estimate was 35.7% (95% CI: 15.0% to 51.3%); this latter result can be interpreted as 35.7% vaccine efficacy through IgG3 V1V2 breadth where the lower 95% confidence bound exceeding 0 provides statistical support for this interpretation. Table 3 shows mediation results for all immune markers that qualified for analysis. Marker-Mediated VE was 28–44% for the six gp70-scaffolded IgG3 V1V2 antigens and <10% for the two IgG3 V1V2 tags-construct antigens.

**Table 3:** Table of mediation effect estimates for all qualifying Month 7 quantitative markers with 95% confidence intervals.

Assay	Non-marker mediated VE^a^	Marker-mediated VE^b^
IgG3 V1V2 breadth	−35.0% (−102%, 9.9%)	35.7% (15.0%, 51.3%)
IgG V1V2 breadth	−43.0% (−147%, 17.1%)	39.3% (0.4%, 63.0%)
IgG3 AE.A244 V1V2 Tags 293F	4.3% (−39.2%, 34.1%)	9.3% (−27.9%, 35.6%)
IgG3 C.1086C V1V2 Tags	20.8% (−12.8%, 44.4%)	−9.7% (−50.0%, 19.8%)
IgG3 gp70–001428.2.42 V1V2	−45.7% (−109%, −1.3%)	40.4% (24.1%, 53.1%)
IgG3 gp70–1012.11.TC21.3257 V1V2	−31.3% (−83.1%, 5.8%)	33.9% (21.1%, 44.6%)
IgG3 gp70-1394C9G1 V1V2	−20.5% (−67.1%, 13.1%)	27.9% (10.4%, 42.1%)
IgG3 gp70-BF1266 431a V1V2	−44.9% (−107%, −1.6%)	40.0% (24.2%, 52.6%)
IgG3 gp70-Ce1086 B2 V1V2	−55.6% (−120%, −10.0%)	44.2% (26.6%, 57.5%)
IgG3 gp70-B.CaseA2 V1V2	−21.3% (−71.7%, 14.3%)	28.4% (11.6%, 42.0%)
ADCC AUC CAP8	0.2% (−41.5%, 29.6%)	13.0% (−12.1%, 32.5%)
ADCC AUC CH58	−11.1% (−79.1%, 31.1%)	21.8% (−13.4%, 46.1%)
ADCC AUC WITO	−5.5% (−95.4%, 43.0%)	17.7% (−41.9%, 52.2%)
CD4+ IFN-g and/or IL-2 Env	−15.3% (−53.1%, 13.2%)	24.6% (3.1%, 41.4%)
CD8+ IFN-g and/or IL-2 Env	−14.5% (−62.4%, 19.3%)	24.1% (−1.3%, 43.2%)

ADCP, antibody-dependent cellular phagocytosis; AUC, area under the curve; Env, envelope; gp, glycoprotein; IFN, interferon; IgG, immunoglobulin G; IL, interleukin; V1V2, variable regions V1 and V2; VE, vaccine efficacy.

a Non-Marker Mediated VE = VE comparing vaccine vs. placebo with marker set to distribution in placebo (VE through other pathways than the Month 7 marker).

b Marker-Mediated VE = VE in vaccinated comparing observed marker vs. hypothetical marker under placebo (VE mediated through the Month 7 marker). Can be interpreted as the proportion of vaccine efficacy mediated through the marker.

## Discussion

In the proof-of-concept Imbokodo efficacy trial, the evaluated heterologous vaccine regimen of Ad26.Mos4.HIV and aluminium-phosphate adjuvanted clade C gp140 protein did not demonstrate efficacy in preventing HIV-1 acquisition in southern African people assigned female sex at birth and with an increased likelihood of HIV acquisition. The lack of significant VE may have been attributed to limited induction of sufficiently high-magnitude and coordinated protective immune responses such as ADCP, ADCC, CD4+ and CD8+ T-cell responses, and IgG3 antibodies to the V1V2 regions of the Env protein; alternatively, the responses induced may not have sufficiently recognised the diversity of clade C viruses that were circulating and to which trial participants were exposed.

This study sought to evaluate whether the immune correlates previously identified in an NHP model [(1) Env-specific IgG antibody, (2) ADCP, (3) T-cell responses measured by ELISpot, and (4) V1V2-specific IgG antibody] translated to also being immune correlates in Imbokodo. The analysis showed that NHP correlates (1) and (2) were not correlates in Imbokodo, whereas (3) (ELISpot T cells) could not be tested as a correlate. Because ELISpot data could not be included in the analysis, instead Env-specific IFN-g and/or IL-2 expression by CD4+ and CD8+ T-cells generated by the ICS assay were evaluated as markers, and neither showed evidence as a correlate. Potential reasons for the lack of correlate translation include differences between the NHP–SHIV challenge model and the real-world exposure context of Imbokodo: for example, the SHIV challenge does not reflect the breadth or diversity of exposing viruses that occurred in Imbokodo, which were much more diverse and distinct from the mosaic vaccine sequences. Moreover, the transmission route differed between intrarectal in NHPs vs. presumably mostly intravaginal in Imbokodo, and the NHP challenges occurred weekly over 6 months post final vaccination whereas the exposures in Imbokodo occurred at unknown times between 1 and 18 months post third vaccination. Moreover, the NHPs had no genital infections whereas 32% of Imbokodo participants had a sexually transmitted infection detected at screening.

However, the NHP correlate (4) (V1V2 antibodies) did show evidence of translating to a correlate in Imbokodo. Indeed, given the point estimate of overall VE of 14.1%, one potential scenario is that while most vaccine recipients were not protected, a small subgroup with specific immune responses were protected. The analyses reported here support the hypothesis that high IgG3 V1V2 levels marked this small, protected subgroup. Specifically, point estimates by multiple methods supported IgG3 breadth to V1V2 antigens as an inverse correlate of risk, with estimated risk of HIV-1 diagnosis 7-fold lower for per-protocol vaccine recipients with a breadth threshold at the tail-end of the population (above the 95th percentile) compared to that for the entire group of per-protocol vaccine recipients. Correlates of protection analyses provided support in the same direction, with controlled VE demonstrating a jump-like increase from 12% to 81% over the span of IgG3 V1V2 breadth values from placebo-like responses up to the 95th percentile, although the associated confidence intervals were quite wide. Mediation analysis also supported this trend, yielding an estimate of 35.7% vaccine efficacy mediated through IgG3 V1V2 breadth. Only 9.4% of vaccine recipients achieved IgG3 V1V2 breadth above 667 MFI that was associated with at least 50% vaccine efficacy. Exploratory analyses of how the individual antigens in the IgG3 V1V2 breadth panel influenced the correlate, suggested that gp70-scaffolded V1V2 antigens provided greater discrimination of cases from non-cases than tags-construct antigens. Moreover, the IgG3 binding to the individual V1V2 antigens demonstrated the smallest HRs for those antigens that preferentially bind V2i-like antibodies, supporting the hypothesis that binding to conformation-dependent structures within V1V2 is relevant.^10^ We stress that the lack of statistical significance implies these results should not be interpreted as demonstration of a V1V2 antibody correlate, but rather as valid hypothesis-generating results. They are valid because the nature of the estimated correlate—where only an ∼10% subgroup had VE—is a scenario for which Imbokodo had low statistical power to demonstrate a correlate.

Intriguingly, the only HIV VE trial to demonstrate partial VE, RV144, with an estimated 36% VE from 0 to 24 months,^27^ also supported IgG3 V1V2 antibodies as a correlate of risk and protection. In RV144, both positive IgG3 response and magnitude of IgG3 against the four of the V1V2 antigens studied here were also studied in Imbokodo were inverse correlates of risk of HIV-1 acquisition.^28^ However, response magnitudes against all four antigens were all much lower in Imbokodo than in RV144, and the fact that VE was also lower in Imbokodo is consistent with an interpretation that IgG3 V1V2 antibodies were a correlate of protection in both trials.

If IgG3 V1V2 antibodies do provide some protection against HIV-1 acquisition, it is likely not via neutralization. We have previously assessed the ability of the Imbokodo vaccine regimen to induce neutralizing antibody responses, and found that virtually none of these antibodies are induced, apart from some antibodies to easily-neutralised tier 1 HIV-1 strains.^3^ For this reason, no neutralizing antibodies were assessed in this efficacy trial. There are, however, numerous other ways in which IgG3 antibodies can offer protection against HIV-1 acquisition, such as through activating phagocytes or engagement of Fc receptors.^29^ Multiple studies have shown that IgG3 isotype antibodies can show not only improved HIV-1 neutralization, but also improved Fc effector functions, compared to their non-IgG3 isotype counterparts.^30^, ^31^, ^32^ Moreover, given that results suggestive of IgG3 V1V2 antibodies as correlates were obtained in two different trials as discussed above, host genetics may influence whether or not vaccine recipients are able to generate IgG3 V1V2 antibodies and the nature of the response to these antibodies. Genetic comparisons of both the Ig and FcR loci plus additional genes that are involved in antibody class-switching, using selected samples (case/non-case, with and without IgG3 V1V2) across multiple HIV-1 vaccine efficacy trials would be needed to investigate these questions.

Limitations of this study include that the antigens used in the immunogenicity assays were not perfectly matched to the sequences of HIV-1 circulating when this trial was conducted; the fact that the ELISpot data could not be included in the analysis, as discussed above; the relatively small number of breakthrough cases, which limited precision and statistical power to characterize immune correlates, especially given the skewed distributions of the IgG3 V1V2 markers; and the fact that the CoP analyses make standard causal inference assumptions of no unmeasured confounding, positivity, and consistency. Strengths of the study include the use of qualified or validated assays, ensuring that only high-quality immune response data were used for the markers selected for correlates analysis; the use of data from a randomized, placebo-controlled efficacy trial, considered “gold standard data”; and the application of multiple distinct statistical frameworks to assess the selected markers as correlates.

The generalisability of the results of this correlates analysis is limited by the same factors as those for the primary efficacy and safety results.^7^ Specifically, the Imbokodo trial enrolled only participants assigned female sex at birth, and it is unknown whether the same results would be seen in participants assigned male sex at birth. Moreover, the study was conducted in five countries in southern Africa, with most participants enrolled in South Africa where clade C predominates. Thus, our results may not be generalisable to other regions of the world, or to the context of a different dominant clade.

In conclusion, the trends supporting IgG3 V1V2 antibody levels as a potential correlate of protection, combined with the previous RV144 results on IgG3 V1V2 antibodies as an immune correlate, suggest that increased focus should be placed on the V1V2 region in HIV-1 vaccine immunogen design with the goal of inducing a higher V1V2 antibody response rate and higher levels of broader, conformationally relevant V1V2 antibodies among vaccine recipients. This could potentially be achieved by incorporating Env into vaccine constructs that present the V1V2 region in a configuration optimal for inducing conformational V1V2 antibodies (e.g., Env from clade E.A244 used in RV144) and/or including boosting antigens that enhance production of V1V2-targeting antibodies.^10^^,^^12^^,^^33^^,^^34^

## Contributors

Conceptualization: AK, JvD, OD, LC, DHB, GF, SCDR, MJM, EAN, DJS, GDT, WW, SN, MGP, HS, JHur, JHen, KES, PBG.

Data curation: JvD, OD, RB, CM, YF, EEG, PBG, EB, LP, TvdK, JHep, SS, LZ, DG, GDT, WW, DJS.

Formal analysis: AK, JvD, OD, RB, CM, SK, DCB, OH, MJ, AL, YF, EEG, LHF, SN, WW, PBG.

Funding acquisition: LC, GEG, DHB, MJM, GDT, PBG, MGP, FT, DJS.

Investigation: JHep, SS, LZ, DG, DM, SK, FLO, SS-O, TK, BD, RZ, LP, TvdK, ES, SB, EB, FT, LL, SB.

Methodology: AK, RB, CM, DCB, YF, JHep, KES, LvdL, EEG, BK, PBG, Project administration: JGK, LC, HG, DHB, GF, SCDR, MJM, EAN, DJS, GDT, KES, PBG, JvD, MGP, LP, EB.

Resources: JHur, JHen, JGK, LC, KM, GEG, SZP.

Software: AK, RB, CM, DCB, YF, LvdL, EEG, BK, PBG.

Supervision: DHB, GF, SCDR, MJM, EAN, DJS, KES, GDT, PBG, MGP, HS, JHur, JHen, FT.

Validation: AK, JvD, OD, RB, SCDR, EAN, DJS, KES, GDT, PBG, LP, TvdK.

Visualization: AK, JvD, OD, JHep, RB, SS, LZ, CM, YF, LNC, EAN, DJS, GDT, PBG.

Writing—original draft: AK, JvD, LNC, GDT, PBG.

Writing—review and editing: All coauthors.

∗Members of the Imbokodo Study and Correlates Group who are not co-authors of this work are Collaborators on this work, as defined in Ref.^35^

EAN, DJS, GDT, and PBG accessed and verified the underlying data reported in the manuscript. PBG was responsible for the decision to submit the manuscript. All authors read and approved the final version of the manuscript.

## Data sharing statement

The complete de-identified patient data set will be made available at the public-facing HVTN website (https://atlas.scharp.org/cpas/project/HVTN%20Public%20Data/begin.view?) when the trial is completed. Statistical analyses were performed using publicly available code (https://github.com/Larsvanderlaan/npthreshold, https://github.com/CoVPN/correlates_reporting2/commit/4fcd35f7d33bf8a5a0c25b399f511ef4aced6903, and Refs.^13^^,^^17^).

## Declaration of interests

TvdK has a patent application with Johnson & Johnson and has stocks in Johnson & Johnson. BK received internal support for the present manuscript from her employer (Los Alamos National Laboratory). In the past 36 months, she received support for attending meetings and/or travel from NIH NIAID and from the Gates foundation. Her institution (LANL) had a patent on this work, although she did not receive any personal funds through this patent and was not involved with the licensing of the design to Johnson & Johnson. DHB has a patent on the mosaic HIV vaccine, but no royalties. FT was an employee of Janssen/Johnson & Johnson at the time the work was conducted and owns stock in Johnson & Johnson. LL received support from Janssen Infectious Diseases BV, Beerse, Belgium for travel expenses to attend HIV conferences and has stock or stock options in Johnson & Johnson. JvD, MGP, WW, TvdK and JHen are employees of Janssen/Johnson & Johnson and hold stock or stock options in Johnson & Johnson. WW has a patent planned, issued, or pending with Johnson & Johnson. SCDR had contracts in the past 36 months to perform immunogenicity testing for Janssen, Sanofi, and Moderna. HS and DJS were employees of Janssen Vaccines & Prevention BV and had stock and/or stock options in Johnson & Johnson at the time the work was conducted. SN was an employee of Janssen Infectious Diseases BV and had stock and/or stock options in Johnson & Johnson at the time the work was conducted. LP was an employee of Janssen Vaccines & Prevention BV at the time the work was conducted. GDT has received consulting fees for a scientific consulting session. All other authors have no potential competing interests to disclose.

Funding for the Imbokodo Study and Correlates Group is the same as listed in “Acknowledgments” for the current work.

## Appendix A Supplementary data

Supplementary Tables and Figures

REDACTED_SAP-FD-VAC89220HPX2008-1251709
