# Supplementary material for: Immune correlates analysis of the Imbokodo (HVTN 705/HPX2008) efficacy trial of a mosaic HIV-1 vaccine regimen evaluated in Southern African people assigned female sex at birth: a two-phase case-control study
Source: eBioMedicine. 2024 Sep 4;108:105320. doi: 10.1016/j.ebiom.2024.105320 (PMC11404224; doi:10.1016/j.ebiom.2024.105320)
Supplement: Supplementary Tables and Figures [file mmc1.docx]

**Supplementary Appendix**

# Imbokodo Study and Correlates Group*

Jon Allagappen, Jessica Andriesen, Alison Ayres, Saman Baral, Linda-Gail Bekker, Asiphe Besethi, Caroline Borremans, Esmee Braams, Caroline Brackett, William Brumskine, Roma Chilengi, Rachel Choi, Thozama Dubula, Jaiden Seongmi Dumas, Brooke Dunn, Radhika Etikala, Zelda Euler, Sarah Everett, Nigel Garrett, Huub Gelderblom, Katherine Gill, Kevin Gillespie, Dimitri Goedhart, Erik Goosmann, Shannon Grant, Ellie Hands, Barton Haynes, Bronwill Herringer, Zaheer Hoosain, Mina Hosseinipour, Portia Hunidzarira, Julia Hutter, Mubiana Inambao, Craig Innes, Taylor Keyes, William Kilembe, Philippus Kotze, Sheena Kotze, Fatima Laher, Imre Laszlo, Erica Lazarus, Hua-Xin Liao, Yong Lin, Helen Lu, Judith Lucas, Mookho Malahleha, Tara McNair, Peter Meerts, Zinhle Mgaga, Mahlodi Montlha, Boitumelo Mosito, Andrew Moultrie, Sarah Mudrak, Valérie Oriol-Mathieu, Marcella Sarzotti-Kelsoe, Matson Tso Mathebula, Mitch Matoga, Rachael McClennen, Pamela Mda, Peter Meerts, Vimla Naicker, Logashvari Naidoo, Cindy-Ann Okkers, Saleha Omarjee, Hella Pasmans, Tricia Philip, Abraham Pinter, Annah Pitsi, Ornelia Ramos, April Randhawa, Sanne Roels, Shamiska Rohith, Lucy Rutten, Jerald Sadoff, Gabriela Salinas, Yvonne Salzgeber, Lorenz Scheppler, Katharine Schwedhelm, Nicolette Schuller, Angelina Sharak, Sherry Stanfield-Oakley, Carrie Sopher, Terence Tafatatha, Simbarashe G. Takuva, Chan Tang, An Vandebosch, Edna Viegas, Valentin Voillet, Frank Wegmann, Mo Weijtens, Stephany Wilcox, Anthony Williams, Chenchen Yu, Pei-Chun Yu, Olive Yuan, and Xuehan Zhang.

*Members of this group are Collaborators on this work.

## Case-Control Sampling Plan

The participant eligibility criteria for sampling into the case-control study were as follows:

1. The participant belongs to the per-protocol cohort.
2. The participant is defined as a case if they received vaccine or placebo and the primary endpoint of HIV-1 infection was diagnosed after the Month 7 visit and before or at the Month 24 visit, with no evidence of HIV infection at any visit before or at the Month 7 visit.
3. The participant is defined as a control if the participant received vaccine or placebo and reached the Month 27 visit HIV-negative. If the participant reached the Month 27 visit HIV-negative but was diagnosed with the primary endpoint at a later time point, then it is required that retrospective testing has been completed and supports the HIV-negative status at the Month 27 visit or a later visit if the Month 27 visit was missed. An eligible control must also meet all of the following criteria:
   1. The qualifying Month 27 or post-Month 27 visit was completed before study unblinding.
   2. The participant has an available BMI value.

Following the case and control definitions above, all cases in the vaccine group and 6 randomly sampled cases in the placebo group, with representation over time, were included in the case-control study. Controls were randomly sampled in a 5:1 control:case ratio in the vaccine group and in a 1:1 control:case ratio in the placebo group from the pools of eligible controls. Controls were frequency-matched to cases on treatment (vaccine vs. placebo), region (Republic of South Africa [RSA] vs. non-RSA), and BMI (<25, ≥25 to <30, ≥30 kg/m^2^). Table S1 summarises the stratum-specific sample sizes:

Table S1. Numbers of cases and controls to be included in the case-control study, per treatment group (vaccine versus placebo), region (RSA versus other [non-RSA]), and BMI category. x_1_, y_1_, x_2_, y_2_, x_3_, and y_3_ denote the numbers of eligible vaccinated cases in the study in their respective category (defined by region and BMI category).

| **Treatment** | **Group** | **RSA** | | | **Non-RSA** | | |
| --- | --- | --- | --- | --- | --- | --- | --- |
|  |  | **BMI < 25** | **25 ≤ BMI < 30** | **BMI ≥30** | **BMI < 25** | **25 ≤ BMI < 30** | **BMI ≥30** |
| Vaccine | Cases | x_1_ | x_2_ | x_3_ | y_1_ | y_2_ | y_3_ |
|  | Controls | 5x_1_ | 5x_2_ | 5x_3_ | 5y_1_ | 5y_2_ | 5y_3_ |
| Placebo | Cases | 1 | 1 | 1 | 1 | 1 | 1 |
|  | Controls | 1 | 1 | 1 | 1 | 1 | 1 |

Cases were selected regardless of how many biospecimens they provided. We note that the definition of controls does not impose any restriction on the availability of participants’ samples. Thus, controls were also selected regardless of how many biospecimens they provided. This choice is made to avoid introducing sampling biases in controls relative to cases through restricting sampling to those participants who meet criteria a and b of the definition of a control, and whose specimens are neither missing nor have low volume.

Sampling eligibility disregards pre-exposure prophylaxis (PrEP) use: Participants are eligible regardless of whether they have accessed the PrEP referral programme, self-reported PrEP, or had a positive dried blood spot test result. This choice is made based on evidence for low PrEP use in this trial. For each sampled participant (including cases and controls), serum or peripheral blood mononuclear cell (PBMC) samples from the following time points were tested for ELISA, ELISpot, and the assays that, based on the pilot study, were selected for inclusion in the case-control analysis (ADCC, ADCP, BAMA): Month 0 and Month 7. For ICS, only Month 7 samples were analyzed.

Additionally, the Month 0 serum samples of all sampled participants were tested in the Ad26 virus neutralisation assay (VNA) assay. The evaluation of pre-existing neutralising antibodies (Nabs) against the Ad26 vector was included in the pilot immunogenicity study as a potential Baseline Immunogenicity Predictor (BIP). To that extent, Month 0 samples of the selected cases and controls were analysed for Ad26 Nabs.

Lack of available biospecimens in some participants would preclude running some or all of the assays at some of the selected time points. Once the list of participants was generated, available vials/biospecimens were prioritised together with the labs, considering the importance of each assay and their specific requirements (e.g., for some assays, positivity calls performed post-vaccination/baseline may require an available Month 0 measurement).

Participants selected for inclusion in the cohort of the pilot study were excluded from the sampling frame used to generate the case-control cohort. The exclusion of participants already in the pilot cohort reduced the probability of sampling participants into the case-control cohort who had contributed samples to the pilot study and might no longer be able to contribute vials/biospecimens in sufficient numbers to run all assays at selected time points.

**Supplementary Methods**

*BAMA Antigen Selection*

HIV-1 envelope and V1V2 antigens were selected by aligned vaccine envs (Mos 1, C97) with breadth panel^1^ to select existing reagents that best represent diversity of Env in southern Africa. Six envs with matching gp140, gp120 and V1V2 (subtypes C, A) were selected to examine the case controls (Figure S1).

**Figure S1. A) V2 linear epitope alignment and B, C) phylogenetic trees including the Imbokodo vaccine envs and the 6 envs selected for BAMA antigens.** A) V2 linear epitope (HXB2 positions 160-180) amino acid alignment of the three vaccine inserts, consensus C, and 6 BAMA antigens (in the green box) selected from the larger panel of antigens.^1^ In blue are highlighted the residues found in at least 3% of envs in the Los Alamos National Laboratory (LANL) database. Dashes represent amino acids matching the Ad26.Mos1 vaccine shown in full at the top. B, C) Phylogenetic trees of a total of 1,549 env sequences comprising: the 3 vaccine inserts (red open symbols), 6 BAMA antigens (red solid triangles), 71 placebo envs [solid circles, colour coded by (B) country or (C) clade], and 1,397 envs downloaded from the LANL database [open circles, colour coded by (B) country or (C) clade], sampled between the years 2000 and 2021, and representing eight different African countries and eight distinct subtypes. The tree was reconstructed from the global amino acid alignment, gap-stripped at 80% for clade stability, using the FastTree software^2,3^ using the Jones-Taylor-Thornton (JTT) evolutionary model and CAT single rate approximation.

**
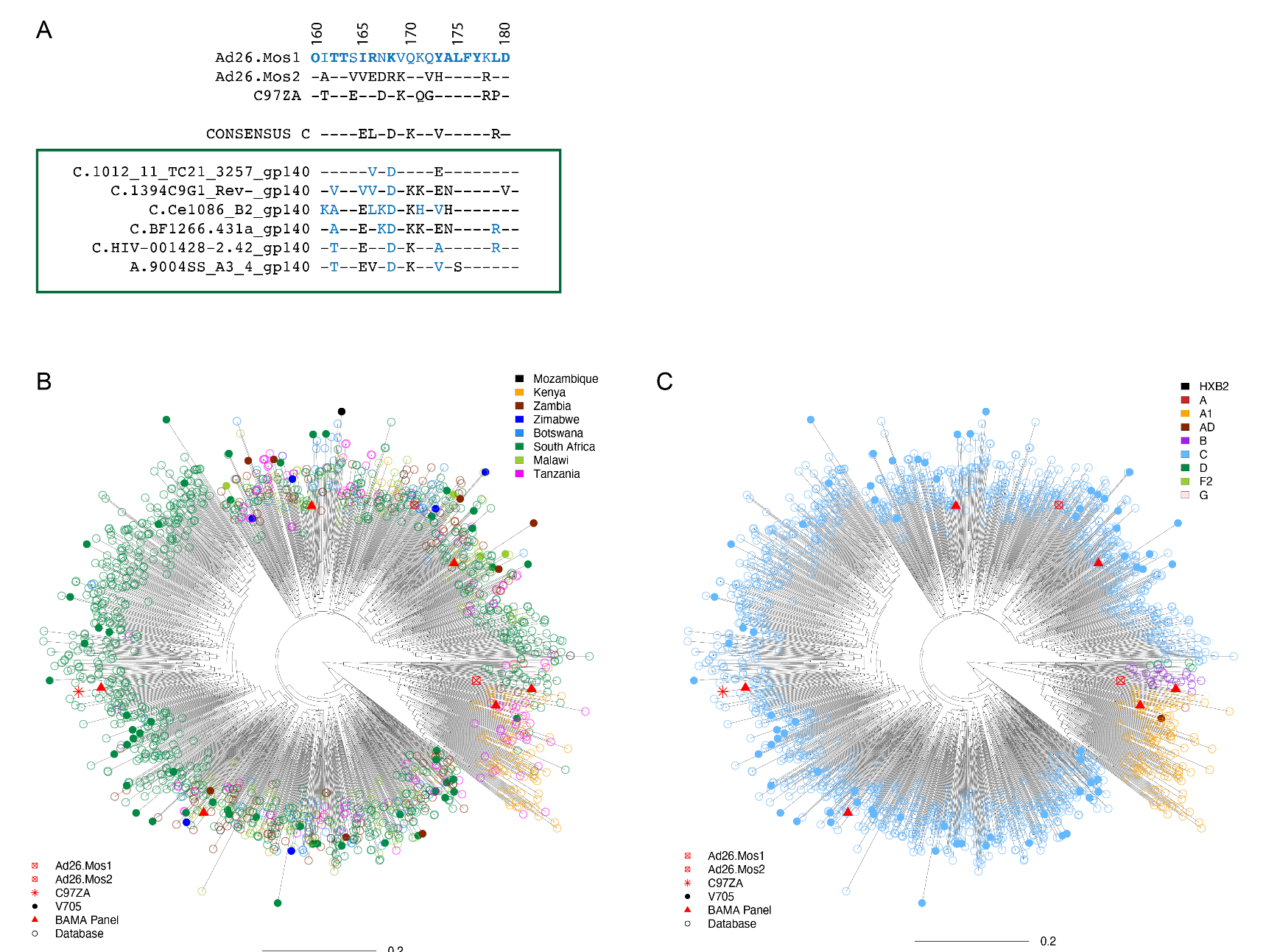
**

*Multi-epitope Function (Primary Marker)*

The multi-epitope function primary marker was calculated using the ICS T cell markers and not the ELISpot marker, where inclusion or exclusion of the ELISpot marker in the combination marker had no meaningful impact given the high concordance (r = 0·99).

*IgG3 V2i and V2p Breadth Scores*

The IgG3 V2i breadth score was computed as the maximal diversity-weighted (MDW) linear combination of IgG3 readouts to the gp70-001428.2.42 V1V2, gp70-1012.11.TC21.3257 V1V2, and gp70_B.CaseA2 V1V2 antigens. It was computed the same way as the primary variable IgG breadth score, except restricting to three antigens rather than including all eight in Table S2. The IgG3 V2p breadth score was computed in the same way, restricting to the five antigens gp70-1394C9G1 V1V2, gp70-BF1266 431a V1V2, gp70-Ce1086 B2 V1V2, C.1086C V1V2 Tags, and AE.A244 V1V2 Tags/293F antigens.

*IgG3 V1V2 Breadth Sensitivity Analysis*

Four exploratory markers were added as a sensitivity analysis of IgG3 V1V2 breadth: IgG340mdw V1V2 trunc1, mdw_xassay_select_igg3v1v2_trunc1, IgG340mdw_V2i_trunc1, and IgG340mdw_V2p_trunc1. These four MDW breadth score markers are the same as the previous MDW breadth score markers (with the text tag “trunc1” dropped) with difference as follows:

For the previous markers, before deriving the MDW score, net MFIs were truncated between 100 and 22000, and nonresponder net MFI was set to 100. For the trunc1 markers, before deriving the MDW score, net MFIs were truncated between 1 and 22000, and nonresponder net MFfonI was set to 1.

##

## Table S2: Viral clade/group of each of the eight antigens whose binding antibody readouts were used to calculate the IgG3 V1V2 breadth score for the BAMA assay

| **Breadth panel** | **Antigen** | **Clade/Group** |
| --- | --- | --- |
| V1V2 | AE.A244 V1V2 Tags/293F | AE |
|  | C.1086C_V1V2 Tags | C |
|  | gp70_B.CaseA2 V1V2 | B |
|  | gp70­1012.11.TC21.3257 V1V2 | C |
|  | gp70­1394C9G1 V1V2 | C |
|  | gp70­BF1266_431a_V1V2 | C |
|  | gp70­001428.2.42 V1V2 | C |
|  | gp70­Ce1086_B2 V1V2 | C |

## Table S3: Viral clade/group of each of the 13 antigens whose binding antibody readouts were used to calculate the IgG3 Env breadth score for the BAMA assay

| **Breadth panel** | **Antigen** | **Clade/Group** |
| --- | --- | --- |
| gp120 | Con 6 gp120/B | M |
|  | 1394C9_G1.D11gp120.avi | C |
|  | 1428_D11gp120.avi/293F | C |
|  | 1012_11.TC21D11gp120.avi | C |
|  | 1086C_D7gp120.avi/293F | C |
| gp140 | gp140 Mos1 fibritin* | N/A ­ Mosaic |
|  | gp140 C97ZA fibritin* | C |
|  | Con S gp140 CFI | M |
|  | 1012_gp140C.avi/293F | C |
|  | 1394C9_gp140C.avi/293F | C |
|  | 1086C gp140C_avi | C |
|  | BF1266_gp140C.avi/293F | C |
|  | 9004S.gp140C.avi | A |

∗ Vaccine-matched antigens are marked with an asterisk, which also are qualified antigens. All other antigens are considered exploratory.

*Immunoassay Development and Validation*

Lower and upper limits of quantification were determined to select the range in which analytes can be detected at both low and high levels accurately. Each coefficient of variability was assessed to measure assay precision and reproducibility. Specific acceptance criteria were set to ensure only high-quality data were used for the markers selected for correlates analysis. Some assays were also evaluated for linearity and dilution potential (see below). All immunoassays were performed blinded to treatment assignment and to case/control status.

Antibody Dependent Cellular Phagocytosis (ADCP): Clinical human serum samples were tested in an ADCP assay to measure the ability of vaccine specific antibodies to mediate phagocytosis. C97ZA gp140 Trimer and Mos1 gp140 Trimer biotinylated antigens (produced in-house by Janssen) conjugated to neutravidin beads (ThermoFisher, catalog #F8776, lot 2181023) were incubated with serum for 3 hours. A monocyte cell line (THP-1, ATCC, catalog #TIB-202, lot #70029994; cell line and lot used in the qualification assays, tested for mycoplasma two passages after their first thaw) was added to the immune complexes and spinoculated at 4ºC followed by incubation at 37ºC. Cells were then analysed for bead internalisation by flow cytometry (bead positive versus bead negative detection) (BD LSRFortessa). Samples were tested in duplicate in each assay.

The magnitude of the ADCP immune response was calculated as a phagocytic score, determined based on the ratio of experimental sample to phosphate-buffered saline (PBS) control. Mean phagocytosis score is defined as: (% bead positive for participant x MFI bead positive for participant) / (% bead positive for PBS only control x MFI bead positive for PBS only control). An assay run was considered valid if the positive control met their predefined criteria for the low-quality control sample (LQC) and the negative (NQC) control sample was below 3.0. The medium (MQC) and high (HQC) quality control samples were used for trending only. The C97ZA gp140 Trimer ADCP is qualified for the following assay characteristics: precision, accuracy, linearity, assay range, and specificity (Table S4). The Mos1 gp140 Trimer is a developed assay. Qualification assays were based on 1:25 dilution, study samples used for analysis were tested at 1:250.

#### Table S4: ADCP assay characteristics for qualification

| **Assay Characteristics** | **Antigen** | **Criteria** | **Status** |
| --- | --- | --- | --- |
|  | **C97ZA g140** |  |  |
| LLOQ | 2.87 | NA | NA |
| ULOQ | 16.11 | NA | NA |
| Overall Intermediate Precision (%CV IP) | 24% | %CV IP ≤ 30% | Pass |
| Linearity (Slope and 90% CI) | 0.86 (0.79, 0.94) | 90% CI [0.80, 1.25] | Pass |
| Specificity | 100% | Negative response rate >90% | Pass |
| Sensitivity | 100% | Positive response rate >90% | Pass |
| LOD (Mean+3SD) | 1.24 | NA | NA |

%CV, percentage of coefficient of variation; IP, intermediate precision; LLOQ, lower limit of quantitation; LOD, limit of detection; SD, standard deviation; ULOQ, upper limit of quantitation.

**Binding Antibody Multiplex Assay (BAMA):** The HIV-1 BAMA is a flow cytometric-based technology that utilises antibody and antigen interactions to test for the presence of multiple HIV specific IgG3 antibodies (Tables S2 and S3) in serum samples. Partially purified HIV antigens are covalently coupled to specific fluorescent bead sets and are mixed to allow for multiplexing. Serum samples were diluted 1:40 in assay diluent and added to the multiplexed bead sets within a 96-well plate; samples and controls were tested in duplicate. Serum sample and bead sets were mixed for an incubation period of 30 ± 5 minutes at 22 ± 2°C. Following sample incubation, bead sets were washed with assay wash buffer. Wash step was followed by addition of an unconjugated mouse anti-human IgG3 heavy chain secondary antibody (ThermoFisher Scientific, Cat #: 05-3600) and a 30 ± 5-minute incubation period at 22 ± 2°C. Bead sets were washed once again with assay wash buffer and then a tertiary goat anti-mouse IgG antibody conjugated to phycoerythrin (PE) (SouthernBiotech, Cat #: 1030-09) to allow for a measurable fluorescence readout of IgG3 antibody binding was added to the bead sets followed by a 30 ± 5-minute incubation period at 22 ± 2°C. Prior to reading the 96-well assay plate, bead sets were washed with assay wash buffer to remove any unbound material. Bead sets were acquired using a Bio-Rad 200 system with BioPlex manager operating software (version 6.1.1). The readout was background-subtracted mean fluorescence intensity (MFI), where background referred to a plate level control (i.e., a blank well run on each plate).

#### Standard positive and negative controls were included in each assay to ensure specificity and to maintain consistency and reproducibility between assays. The positive control includes purified polyclonal IgG from persons living with HIV (HIVIG; NIH AIDS Reagent Program, Cat #: 3957) using a 10-point standard curve (4PL fit) and CH58 V1V2 IgG.^4^ The negative controls were NHS (HIV-1 seronegative human sera; Sigma-Aldrich,

#### Cat #: H4522) and blank beads (Luminex, Product No: MC10036-04). Additional positive controls included an IgG3 monoclonal antibody (DHVI PPF, Cat #: N/A, Protein name: CH58_IgG3/293i) as a detection control. The BAMA IgG3 assay is qualified for two vaccine antigens: gp140 C97ZA fibritin and gp140 Mos1 fibritin for the following parameters linearity, precision, range, dilutional linearity, specificity, and sensitivity (Table S5). All assays were conducted according to Good Clinical Laboratory Practice Guidelines. Specific characteristics of all commercially obtained secondary and tertiary antibodies are reported on the individual product certificates, technical bulletins, and specification sheets provided by the manufacturers.

V2i and V2p antigenicity were determined by monoclonal antibody binding with known V2i and V2p preference (Figure S20).

**Table S5. BAMA IgG3 assay characteristics for qualification.** The qualification report was reviewed and approved by Duke QADVIP.

| **Assay Characteristic** | **Antigen** | | **Criteria** | **Status** |
| --- | --- | --- | --- | --- |
|  | **C97ZA** | **Mos1** |  |  |
| LLOQ (MFI) | 254 | 241 | NA | NA |
| ULOQ (MFI) | 32480 | 43537 | NA | NA |
| Overall Intermediate Precision (%CV IP) | 18% | 20% | %CV IP ≤ 30% | Pass |
| Linearity (Slope:CI) | 0.96 (0.94-0.97) | 0.95 (0.94-0.97) | 90% CI [0.80, 1.25] | Pass |
| Dilutional Linearity (Slope and 90% CI) | 1.01 (1.00-1.03) | 0.98 (0.97-0.99) | 90% CI [0.80, 1.25] | Pass |
| Specificity | 100% | 100% | Negative response rate >90% | Pass |
| Sensitivity | 100% | 100% | Positive response rate >90% | Pass |
| LOD (MFI) | 9 | 13 | NA | NA |

Enzyme-Linked ImmunoSorbent Assay (ELISA): Clinical human serum samples (heat-inactivated in water bath for 60 ± 5 min at 57 ± 1°C) were tested in a quantitative ELISA designed to measure total IgG antibodies specific to the vaccine-matched HIV Envelope (Env) clade C (C97ZA) or Mos1 antigens (Biogen, Research Triangle Park, NC, USA; generated at Biogen via contract with Janssen). Ninety-six-well Nunc flat-bottom Maxisorp assay plates were coated with 1 µg/mL clade C or Mos1 in PBS (pH 7.4) and incubated overnight at 2 - 8°C. Plates were washed with washing buffer (1X PBS + 0.05% Tween-20) and blocking buffer (2% GBB + 2% foetal bovine serum [FBS]) was added. Plates were incubated for 60 ± 5 min at 23 ± 2°C on a shaker at low speed (appr. 300 rpm), thereafter the blocking buffer was removed. HIV Env reference standard (commercially purchased human serum from Seralab positive for HIV Env antibodies, lot number: BRH1136333), assay controls (high, mid, low and negative QCs were clinical samples provided by Janssen, no commercial antibodies used), and clinical human serum samples were added and incubated for 120 ± 10 min at 23 ± 2°C on a shaker at low speed (appr. 300 rpm) and washed with washing buffer. Mouse anti-human IgG(Fc)- horseradish peroxidase (HRP) conjugate (Jackson ImmunoResearch Laboratories, West Grove, PA, USA, cat no. 209-035-098; RRID: AB_2339090) diluted in 2% GBB was added and further incubated for 60 ± 5 min at 23 ± 2°C and again washed with washing buffer. Tetramethylbenzidine (TMB) substrate (Abcam, Cambridge, UK, Cat # Ab171527) was added and incubated for 20 ± 1 min at ambient temperature protected from light. The colorimetric reaction was stopped using 1 M sulfuric acid and the plate was immediately read at 450 nm absorbance to determine optical density (OD). Serum sample antibody concentration was then determined by interpolating OD response from the standard curve and considered valid when passing the pre-defined sample and plate acceptance criteria.

The HIV clade C and Mos-1 ELISAs are considered validated as all assay characteristics met the predefined acceptance criteria regarding linearity, relative accuracy, precision dilutional linearity, specificity, selectivity, interference, and cut-point (Table S6). The validation was performed according to the Bioanalytical Method Validation Guidance for Industry, published by the US Food and Drug Administration (FDA)/Center for Drug Evaluation and Research (CDER) in May 2018.

#### Table S6. ELISA characteristics for validation

| **Assay characteristic** | **Antigen** | | **Criteria** | **Status** |
| --- | --- | --- | --- | --- |
|  | **Clade C** | **Mos-1** |  |  |
| LLOQ (EU/mL, neat) | 80 | 100 | NA | NA |
| ULOQ (EU/mL, neat) | 14720 | 14608 | NA | NA |
| Overall Intermediate Precision (%CV IP) | 13.2% | 9.2% | %CV IP ≤ 30% | Pass |
| Linearity (Slope and 90% CI) | 1.029 (1.024, 1.034) | 1.001 (0.998, 1.005) | 90% CI [0.80, 1.25] | Pass |
| Dilutional Linearity (Slope and 90% CI) | 0.99 (0.97, 1.01) | 0.94 (0.91, 0.96) | 90% CI [0.80, 1.25] | Pass |
| Homologous specificity (GMR and 90% CI) | 0.09 (0.06, 0.14) | 0.03 (0.02, 0.06) | 90% Upper CL: ≤ 0.25 | Pass |
| Heterologous specificity* (GMR and 90% CI) | 1.00 (0.95, 1.05) | 1.01 (0.99, 1.03) | 90% CI: [0.70, 1.43] | Pass |
| Selectivity (GMR and 90% CI) | 1.10 (1.06, 1.14) | 1.03 (1.01, 1.05) | 90% CI: [0.70, 1.43] | Pass |
| Interference (GMR and Dunnett corrected 90% CI) | Hemolytic: 1.00 (0.94, 1.05)  Lipemic: 0.92 (0.87, 0.97)  Icteric: 0.96 (0.90, 1.02) | Hemolytic: 1.02 (0.98, 1.07)  Lipemic: 0.91 (0.87, 0.96)  Icteric: 1.01 (0.96, 1.06) | 90% CI: [0.70, 1.43] | Pass |
| Cut point | 168 EU/mL | 116 EU/mL | NA | NA |

* Heterologous specificity assessed with RSV-A post-fusion protein.

CI = Confidence Interval, CL = Confidence Limits, %CV= Percent Coefficient of Variation, EU/mL: ELISA Units per milli-Liter, GMR = Geometric Mean Ratio, IP = Intermediate Precision, LLOQ: Lower Limit of Quantitation, NA = not applicable, ULOQ: Upper Limit of Quantitation

ELISpot: Peripheral blood mononuclear cells (PBMCs) were thawed and rested overnight prior to the stimulation with 15-mer peptides (manufactured by the NIH, obtained from the NIH by the Barouch lab) that overlap by 11 amino acids. The peptides represent the HIV Potential T cell Epitope Envelope (PTE ENV).^5^ The CEF pool representing immunodominant CD8+ T cell epitopes within cytomegalovirus (CMV), Epstein-Barr virus (EBV) and influenza was used as a positive control, to assess run validity. All peptide pools were used at a final concentration of 2 μg/ml.

Ninety-six-well hydrophobic polyvinylidene difluoride membrane bottomed plates (Millipore) were coated over night at 4°C with anti-human IFN-γ monoclonal antibody (final concentration 1 μg/ml; Mabtech). Plates were washed with Dulbecco’s phosphate buffered saline DPBS (Gibco) and blocked with R10 medium (Roswell Park Memorial Institute [RPMI] supplemented with 10% bovine serum, 2 mM L-glutamine, 50 μg/ml streptomycin, 50 U/ml penicillin, 1 mM sodium pyruvate, 0.055 mM 2-mercaptoethanol, and 0.01 M 4-(2-hydroxyethyl)-1-piperazineethanesulfonic acid (HEPES) in a humidified incubator at 37°C for 2-4 hours. Subsequently, PBMCs were resuspended in R10 medium and added to the plate at a concentration of 2 x 10^5^ /well.

Mock wells containing PBMCs and media only were supplemented with the equivalent concentration of dimethyl sulfoxide (DMSO) and served as negative controls. Concanavalin A (ConA) was used as an additional positive control for the functionality of each individual control or clinical sample. PBMCs plus peptide antigens or DMSO were tested in three replicate wells and at least one additional well was stimulated with ConA. After incubation at 37°C in 5% CO_2_ for 15 to 20 hours, PBMCs were removed by washing with DPBS. Captured IFN-γ was detected by incubation for 2-4 hours at 37°C with biotinylated anti-human IFN-γ monoclonal antibody at 1 μg/ml. Following incubation, plates were washed and alkaline phosphatase conjugated anti-biotin antibody at 1.33 μg/ml was added and incubated for 2-3 hours. The final wash was followed by the addition of warmed and filtered nitro-blue tetrazolium chloride/5-bromo-4-chloro-3-indolyl phosphate p-toluidine salt (BCIP/NBT chromagen) substrate solution for 7 minutes. The chromagen was discarded and the plates were washed with water and dried in a for at least 24 hours with minimal exposure to light. Spots were counted with a CTL analyser (Shaker Heights, OH) and associated software. Results are expressed as mock subtracted spot-forming cells (SFCs)/10^6^ PBMCs. The assay was qualified for the parameters described in Table S7.

#### Table S7. ELISpot assay characteristics for qualification

| **Assay characteristic** | **Result** |
| --- | --- |
| LLOQ (mock subtracted SFC/10^6^ PBMCs) | 117 |
| ULOQ (mock subtracted SFC/10^6^ PBMCs) | 2373 |
| Overall Intermediate Precision (%CV IP) | 27.86% |
| Linearity (Slope and 90% CI) | 0.87 (0.84, 0.90) |
| Cut point (mock subtracted SFC/10^6^ PBMCs) | 60 |

CI = confidence interval, CV= coefficient of variation, IP = intermediate precision, LLOQ = lower limit of quantification, ULOQ = Upper limit of quantification

Intracellular cytokine staining (ICS): ICS was performed on cryopreserved PBMCs as described.^6^ Briefly, cryopreserved PBMC were thawed, incubated overnight at 37°C/5% CO_2_ in R10 (RPMI 1640 [Gibco BRL Life Technologies, Grand Island, NY, USA]) containing 10% FBS (Nucleus Biologics, San Diego, CA, USA), 2 mM L-glutamine (Gibco BRL Life Technologies), 100 U/ml penicillin G, and 100 μg/ml streptomycin sulfate (Gibco BRL Life Technologies)] and stimulated on Day 2 for six hours at 37°C with either the peptide pools (peptides of 15 amino acids overlapping in sequence by 11 amino acids) listed in Table S8, dimethyl sulfoxide (DMSO, 0.5%; Sigma Aldrich; negative control) or staphylococcal enterotoxin B (SEB, 0.25 µg/mL; Sigma Aldrich, St. Louis, MO, USA; positive control) in the presence of costimulatory antibodies CD28 and CD49d (1 µg/ml, BD Biosciences) and brefeldin A (BFA, 10 µg/ml; Sigma Aldrich). Cells were incubated with ethylenediaminetetraacetic acid (EDTA, 2 mM; Life Technologies) overnight at 4°C, then stained with a 28-color antibody staining panel (Table S9) and acquired on Day 3 on a BD FACSymphony A5 flow cytometer (BD Biosciences). Data were analysed using FlowJo version 9.9.4 (FlowJo LLC).

#### Table S8: Peptide pools

| **Peptide Pool** | **Used in Pilot** | **Used in Case Control** |
| --- | --- | --- |
| J Mos1 gp120 | Y | Y |
| J Mos1 gp41 | Y | Y |
| J Mos1 Gag | Y | N/A |
| 97ZA012 gp120 | Y | N/A |
| 97ZA012 gp41 | Y | N/A |
| J Mos1 RT | Y | N/A |
| J Mos1 RNAseInt | Y | N/A |
| J Mos2 Gag | Y | Y |
| J Mos2 RT | Y | N/A |
| J Mos2 RNAseInt | Y | Y |
| J Mos2S gp120 | Y | Y |
| J Mos2S gp41 | Y | Y |
| CMV pp65 | Y | Y |

Y: yes and N/A: Not applicable

#### Table S9: 28-Color ICS panel used for the characterization of antigen-specific T cell responses. Antibodies were titrated upon first use to find the optimal titre in the staining panel, and new antibody lots bridged against the current lot to ensure maintained performance.

| **Fluorochrome** | **Specificity** | **Clone** | **Supplier** | **Catalog #** |
| --- | --- | --- | --- | --- |
| BUV395 | CD3 | UCHT1 | BD | 563546 |
| Viability | Viability | NA | Invitrogen | L34962 |
| BUV496 | CD45RA | HI100 | BD | 624283 |
| BUV563 | CD19 | SJ25C1 | BD | 612916 |
| BUV661 | CXCR5 | RF8B2 | BD | 741559 |
| BUV737 | CD154 | TRAP1 | BD | 624286 |
| BUV805 | CD8 | SK1 | BD | 612889 |
| V450 | IFN𝛾 | B27 | BD | 560371 |
| BV480 | CD4 | SK3 | BD | 566104 |
| BV570 | CD16 | 3G8 | Biolegend | 302036 |
| BV605 | CCR7 | G043H7 | Biolegend | 353224 |
| BV650 | Integrin B7 | FIB504 | BD | 564285 |
| BV711 | CD25 | 2A3 | BD | 563159 |
| BV750 | CD56 | 5.1H11 | Biolegend | 362556 |
| BV785 | CCR5 | J418F1 | Biolegend | 359132 |
| FITC | TNF𝛼 | Mab11 | Thermo Fisher | 11-7349-82 |
| BB630 | IL-4 | MP4-25D2 | BD | 624294 |
| BB630 | IL-13 | JES10-5A2 | BD | 624294 |
| BB660 | CD14 | MΘP9 | BD | 624295 |
| PerCP-Cy5.5 | Perforin | B-248 | Biolegend | 353314 |
| BB790 | Ki67 | B56 | BD | 624296 |
| PE | MIP-1β | REA511 | Miltenyi | 130-107-700 |
| PE-Dazzle594 | LAG3 | 11C3C65 | Biolegend | 369332 |
| PE-Dazzle594 | TIM3 | F332E2 | Biolegend | 345034 |
| PE-Cy5 | CTLA-4 | BNI3 | BD | 555854 |
| PE-Fire750 | HLA-DR | TU36 | Thermo Fisher | 35-0567-42 |
| PE-Cy7 | PD-1 | EH12.2H7 | Biolegend | 329918 |
| APC | IL-2 | MQ1-17H12 | Biolegend | 500310 |
| Alx700 | Granzyme B | GB11 | BD | 560213 |
| APC-Fire750 | CD38 | HB-7 | Biolegend | 356626 |

**Assay validation**

The IFN-γ and/or IL-2 expression upon stimulation with 97ZA012 gp120, JMos1 gp120 and JMos1 Gag peptide pools for CD4+ and CD8+ T cell subsets was validated for the parameters described in Table S10.

#### Table S10: Assay characteristics

| **Assay characteristics** | **Criterion** | **Peptide / Stimulation** | **T cell subset** | **Results** | **Status** |
| --- | --- | --- | --- | --- | --- |
|  |  |  |  | **CCC positive responders (95% CI: Lower, upper bound)** | |
| Accuracy | 95% lower bound of CCC*>0.70 | 97ZA012 gp120 | CD4+ | 0.80 (0.66, 0.89) | Fail |
|  |  |  | CD8+ | 0.87 (0.71, 0.94) | Pass |
|  |  | J Mos1 gp120 | CD4+ | 0.80 (0.69, 0.88) | Fail |
|  |  |  | CD8+ | 0.87 (0.76, 0.93) | Pass |
|  |  | J Mos1 Gag | CD4+ | N/A | N/A** |
|  |  |  | CD8+ | 0.97 (0.93, 0.98 ) | Pass |
|  |  |  |  | **No. of samples that passed CV<30%** |  |
| Precision | CV<30% | 97ZA012 gp120 | CD4+ | 1/6 | See below |
|  |  |  | CD8+ | 0/6 | See below |
|  |  | J Mos1 gp120 | CD4+ | 5/6 | See below |
|  |  |  | CD8+ | 3/6 | See below |
|  |  | J Mos1 Gag | CD4+ | 2/6 | See below |
|  |  |  | CD8+ | 4/6 | See below |
|  |  |  |  | **No. of samples passed (Range of slope; 90% CI)** | |
| Linearity | 90% CI of the slope [0.8-1.25], slope R ^2 >0.8 | PMA/ionomycin | CD4+ | 3/3 (0.98-0.99; 0.82 - 0.89) | Pass |
|  |  |  | CD8+ | 3/3 (0.99***; 0.82 - 0.90) | Pass |
|  |  |  |  | **% False positive rate across all peptides** |  |
| Specificity | <10% false positivity rate | Peptides listed in Table S8 | CD4+ | All peptides = 0% | Pass |
|  |  |  | CD8+ | All peptides < 6.4% | Pass |
| LLOQ/ULOQ |  |  |  |  | N/D |
| *CCC= Concordance Correlation Coefficient  **Most CD4 responses below level of detection and thus accuracy could not be assessed  *** For all three replicate runs the R2 was 0.99, so hence no range. | | | | | |

LLOQ and ULOQ were not determined during this validation. For accuracy, there were some measures that failed, with the lower bound of the 95% CI falling just below the acceptable threshold of 0.7. For CD4+ 97ZA012 gp120 and J Mos1 gp120, many responses were of low level (<0.1%), and more variability is expected at this level. Additionally, both peptide pools induce some toxicity, also affecting reproducibility.

For precision, calculations at individual sample levels showed a decreased intermediate precision (CV>30%) for several samples, most often because the response levels (frequencies of responding cells) were very low (below 0.03%). For 97ZA012 gp120, most samples failed – in addition to low response magnitudes, this was likely due to a cell toxicity effect observed with this peptide pool. This pool was not used as a stimulation in the case-control samples.

For J Mos1 gp120, for CD4+ T cells there is one sample that failed with a CV of 32%, just above the threshold. For CD8, two samples that failed were low level, and in fact, were not positive based on our statistical positivity testing; one other sample had CV of 32%. For J Mos1 Gag, all the CD4+ T-cell responses were very low and fell below the 0.08% level. The CD8+ T cell responses were higher magnitude in all the participant identification numbers (PTIDs); two of the PTIDs assayed had a singular data point that was an outlier, resulting in a higher CV.

The precision results technically did not pass based on the preset criteria, but for the reasons we next describe, we considered the results acceptable. Overall, one reason for the fails in precision is that the criteria for passing did not include a requirement for the response magnitudes to be detectable (as determined by positivity testing). Additionally, after completion of these validation experiments, based on extensive validation testing for a similar 27-colour ICS assay developed for SARS-CoV-2 vaccine assessments (including FDA review), this revealed that the expected performance of the assay is more appropriately reflected using a CV threshold of 35% and allowing for up to 20% of measurements to exceed this threshold. If these criteria had been established before the validation reported here, all precision assessments would have passed.

Antibody dependent cell-mediated cytotoxicity (ADCC): The Antibody-Dependent Cell-Mediated Cytotoxicity assay was qualified according to Good Clinical Laboratory Procedure (GCLP) under the supervision of the Duke Quality Assurance Unit and has been previously published.^7^ The principle of the assay is to quantify the elimination of HIV-1-infected cells by natural killer (NK) cells using luciferase activity as the final readout. Target cells were generated by infecting and freezing CEM.NKR-CCR5 cells^8^ with HIV-1 infectious molecular clones (IMCs; produced under GCLP guidelines by Dr. Montefiori laboratory, Duke University), each of which represents a full-length infectious HIV-1 isolate. The IMCs expressing the subtype B CH058 (accession number EU289194), WITO (AY835451), SUMA (EU577073), and subtype C CAP8 (FJ443492) HIV-1 envelopes were selected based on a previous publication^9^ to evaluate the breadth of ADCC responses. CEM.NKR-CCR5 cells are regularly tested for Mycoplasma according to GCLP guidelines and never tested positive. Peripheral blood mononuclear cells (PBMCs) were obtained from a HIV-seronegative donor by leukapheresis and cryopreserved until the day of the assay. After thawing and overnight resting in RPMI 1640 (Invitrogen, Carlsbad, CA) supplemented with antibiotics, 10% foetal bovine serum (Gemini Bio-Products, West Sacramento, CA) (R10), and 10 ng/mL of IL-15 (Miltenyi Biotec, GmbH), the PBMCs were used as effector cells at an effector-to-target ratio of 30:1. Target and effector cells were plated in white 96-well half-area plates and co-cultured with 4-fold serial dilutions of trial participant serum starting at the 1:50 dilution. Co-cultures were incubated for 6 hours at 37°C in 5% CO_2_. For each sample, percent specific killing was measured in duplicate at dilutions of 1:50, 1:200, 1:800, 1:3200, 1:12800, and 1:51200. The monoclonal anti-RSV antibody Synagis (Arexis Ab c/o Swedish Orphan Biovitrum AB; Sweden) and a cocktail of HIV-1 monoclonal antibodies (A32, 2G12, CH44, and 7B2) were used as negative and positive controls, respectively. The Synagis mAb was purchased from the clinical pharmacy*.* The recombinant mAbs, used as positive controls, are produced by the Duke Protein Production Facility with a certificate of analysis released for each lot. Different lots are bridged under GCLP guidelines.

In this analysis, the relative luminescence units (RLUs) of the target plus effector wells represents spontaneous lysis in the absence of any source of antibody and is used to calculate and correct for background activity. The percentage of killing was calculated using Eq 1. Negative values were truncated at zero. Pre- and post-vaccination samples from the same participant were measured in the same assay run to correct from baseline activity (Eq 2).

Eq 1:

$$percentage killing=\frac{RLU background-RLU sample or control}{RLU background}$$

Eq 2:

$$Baseline- subtracted percentage killing=percengate killing post- vaccination-\mathrm{percengate}killing pre-vaccination$$

The percentage killing was plotted on the y-axis and the dilution was plotted on the x-axis. The reportable value is the partial area under the baseline-subtracted curves (pAUC), calculated using the trapezoidal rule on the first four dilutions of the baseline-subtracted curves. A response is defined as positive if the peak baseline-subtracted percentage killing activity was greater than or equal to 10% for either the 1:50 or 1:200 dilution.

The assay was qualified for the parameters described in Table S11:

#### Table S11: ADCC characteristics for qualification

| **IMC** | **IMC** | | |
| --- | --- | --- | --- |
|  | CAP8 | CH58 | WITO |
| LLOQ (baseline subtracted pAUC) | 14.577 | 20.577 | 10.567 |
| ULOQ (baseline subtracted pAUC) | 25.881 | 31.352 | 37.892 |
| % CV IP | 39.9% | 45.5% | 43.3% |

* Heterologous specificity assessed with RSV-A post-fusion protein.

pAUC = peak Area Under the Curve, %CV= Percent Coefficient of Variation, IP = Intermediate Precision, LLOQ: Lower Limit of Quantitation, NA = not applicable, ULOQ: Upper Limit of Quantitation

## Figure S2. Flowchart of participants from enrolment through to case-cohort sampling for inclusion in the analysis. MITT, modified intent to treat.

**
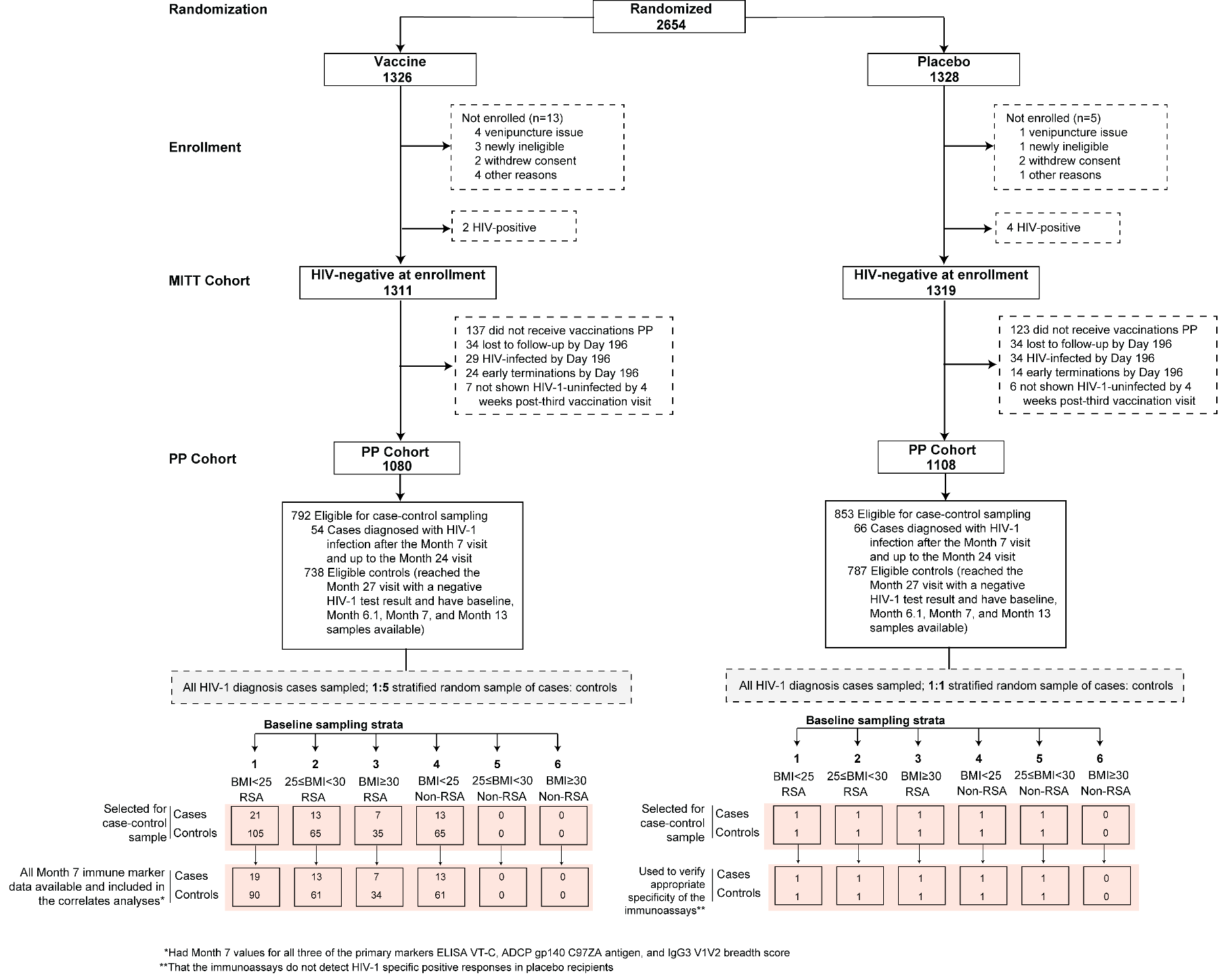
**

# Pilot Immunogenicity Experiments

In total, 60 trial participants (n=50 from active arm, n=10 from placebo arm) were randomly selected from participants in the full immunisation set (receiving all 4 vaccinations within the respective visit windows) that reached the Month 27 visit, remained HIV negative, and had samples available from Months 0, 7, 13, and 24 as well as data on BMI, country, and age. The assays for the pilot immunogenicity experiments are shown in Table S12.

## Table S12. Assays for the pilot immunogenicity experiments.

| **Assay** | **Details** |
| --- | --- |
| ELISA | Two antigens;  Mos1 gp140,  C97ZA gp140 |
| ICS | 9 peptide pools: see Table S8 |
| ADCP | Two antigens;  Mos1 gp140, C97ZA gp140 |
| ELISpot | ENV PTE |
| ADCC | 4 IMCs:  CAP8, SUMA, WITO, CH5 |
| BAMA | gp140, gp120 and V1V2 panel |

PTE, potential T-cell epitope.

Figure S3. Distribution of the IgG gp140 C97ZA responses at Month 7, Month 13, and Month 24 in the pilot immunogenicity experiments, stratified by randomisation arm (vaccine vs. placebo). Each violin plot contains a boxplot showing the estimated 25th, 50th, and 75th percentiles of the marker distribution, as well as a (rotated) kernel density estimate of the marker probability density function. Boxplots are based on observed response magnitudes among responders (filled colour-coded circles) and non-responders (grey triangle outlines). Panels show the actual numbers of participants with available assay data above the response rates and GM estimates, with the latter two accounting for inverse-probability-of-sampling weights. GM, geometric mean; LLoQ: lower limit of quantitation.


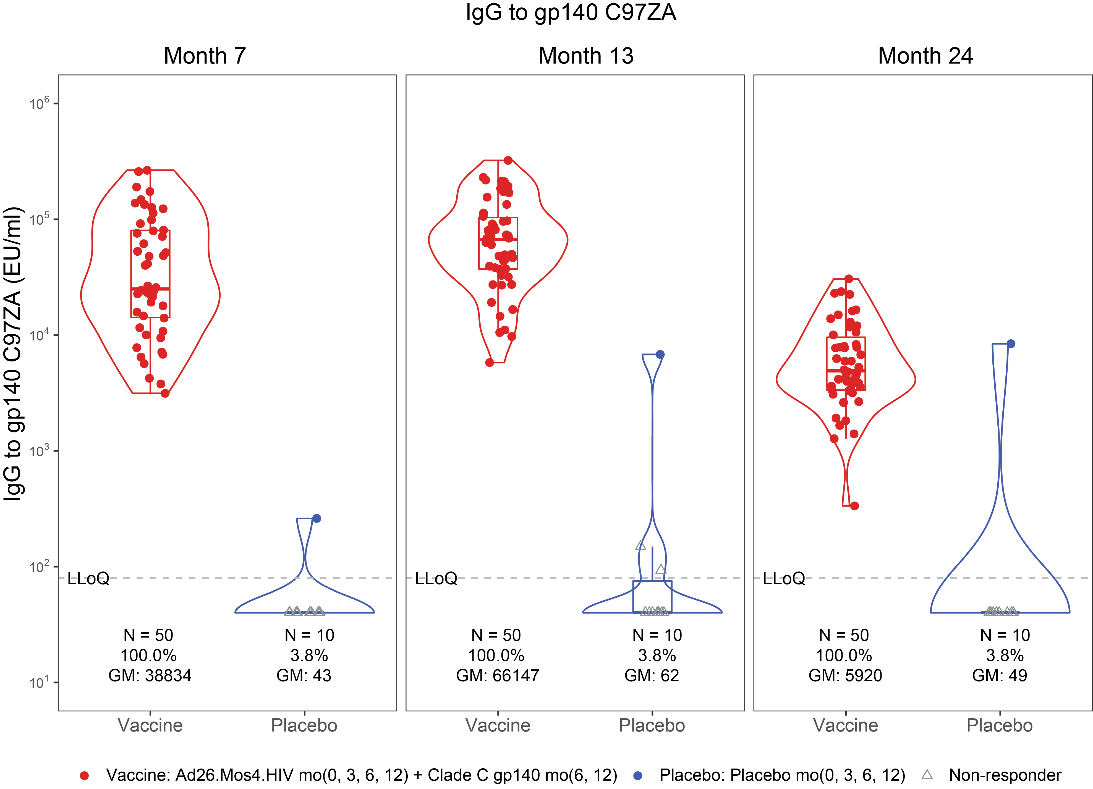


Figure S4. Distribution of ADCP gp140 C97ZA scores at Month 7, Month 13, and Month 24 in the pilot immunogenicity experiments, stratified by randomisation arm (vaccine vs. placebo). Each violin plot contains a boxplot showing the estimated 25th, 50th, and 75th percentiles of the marker distribution, as well as a (rotated) kernel density estimate of the marker probability density function. Boxplots are based on observed response magnitudes among responders (filled colour-coded circles) and non-responders (grey triangle outlines). Panels show the actual numbers of participants with available assay data above the response rates and median scores, with the latter two accounting for inverse-probability-of-sampling weights.


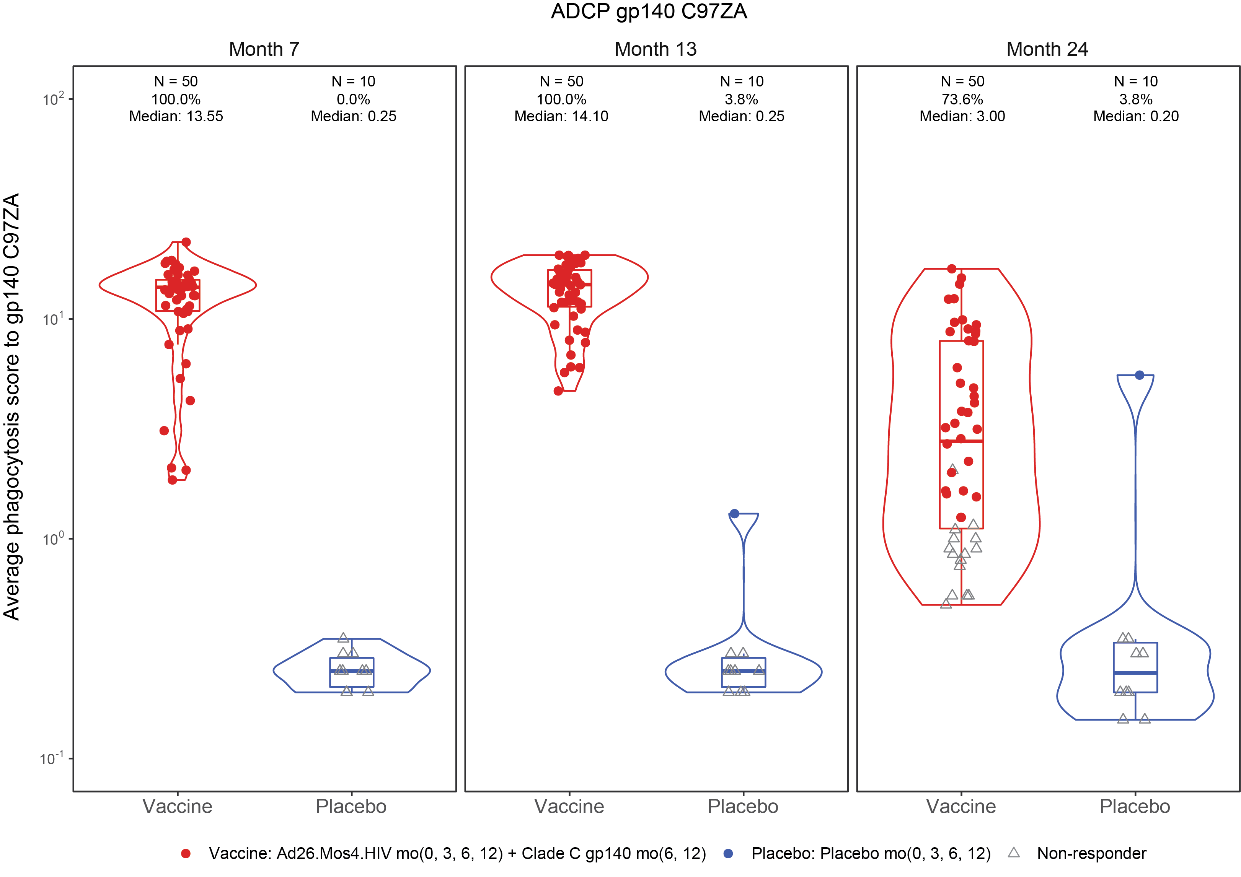


Figure S5. Distribution of ELISpot PTE Env responses at Month 7, Month 13, and Month 24 in the pilot immunogenicity experiments, stratified by randomisation arm (vaccine vs. placebo). Each violin plot contains a boxplot showing the estimated 25th, 50th, and 75th percentiles of the marker distribution, as well as a (rotated) kernel density estimate of the marker probability density function. Boxplots are based on observed response magnitudes among responders (filled colour-coded circles) and non-responders (grey triangle outlines). Panels show the actual numbers of participants with available assay data above the response rates and median values, with the latter two accounting for inverse-probability-of-sampling weights.


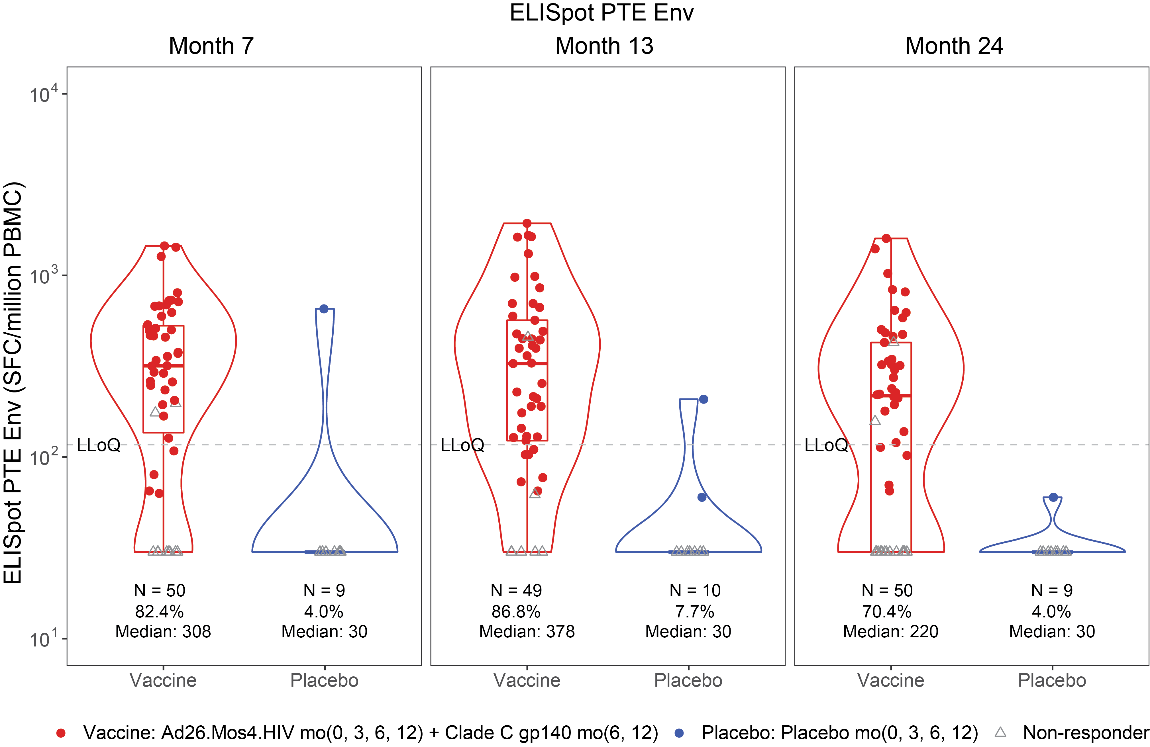


Figure S6. Distribution of IgG3 V1V2 breadth responses at Month 7, Month 13, and Month 24 in the pilot immunogenicity experiments, stratified by randomisation arm (vaccine vs. placebo). Each violin plot contains a boxplot showing the estimated 25th, 50th, and 75th percentiles of the marker distribution, as well as a (rotated) kernel density estimate of the marker probability density function. Panels show the actual numbers of participants with available assay data above the geometric means (GMs), with the latter accounting for inverse-probability-of-sampling weights.


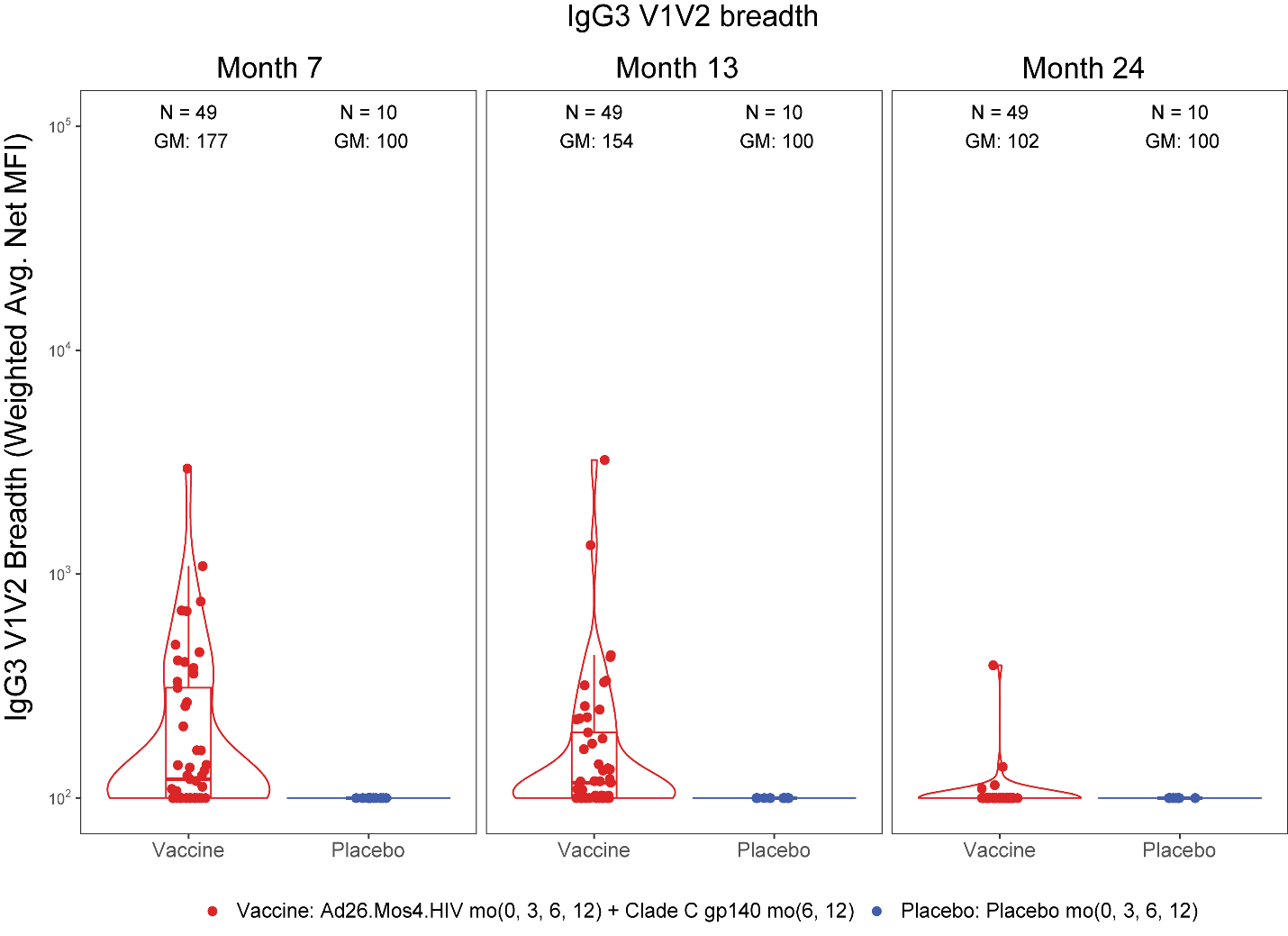


Figure S7. Distribution of IgG3 gp120+gp140 breadth responses at Month 7, Month 13, and Month 24 in the pilot immunogenicity experiments, stratified by randomisation arm (vaccine vs. placebo). Each violin plot contains a boxplot showing the estimated 25th, 50th, and 75th percentiles of the marker distribution, as well as a (rotated) kernel density estimate of the marker probability density function. Panels show the actual numbers of participants with available assay data above the geometric means (GMs), with the latter accounting for inverse-probability-of-sampling weights.


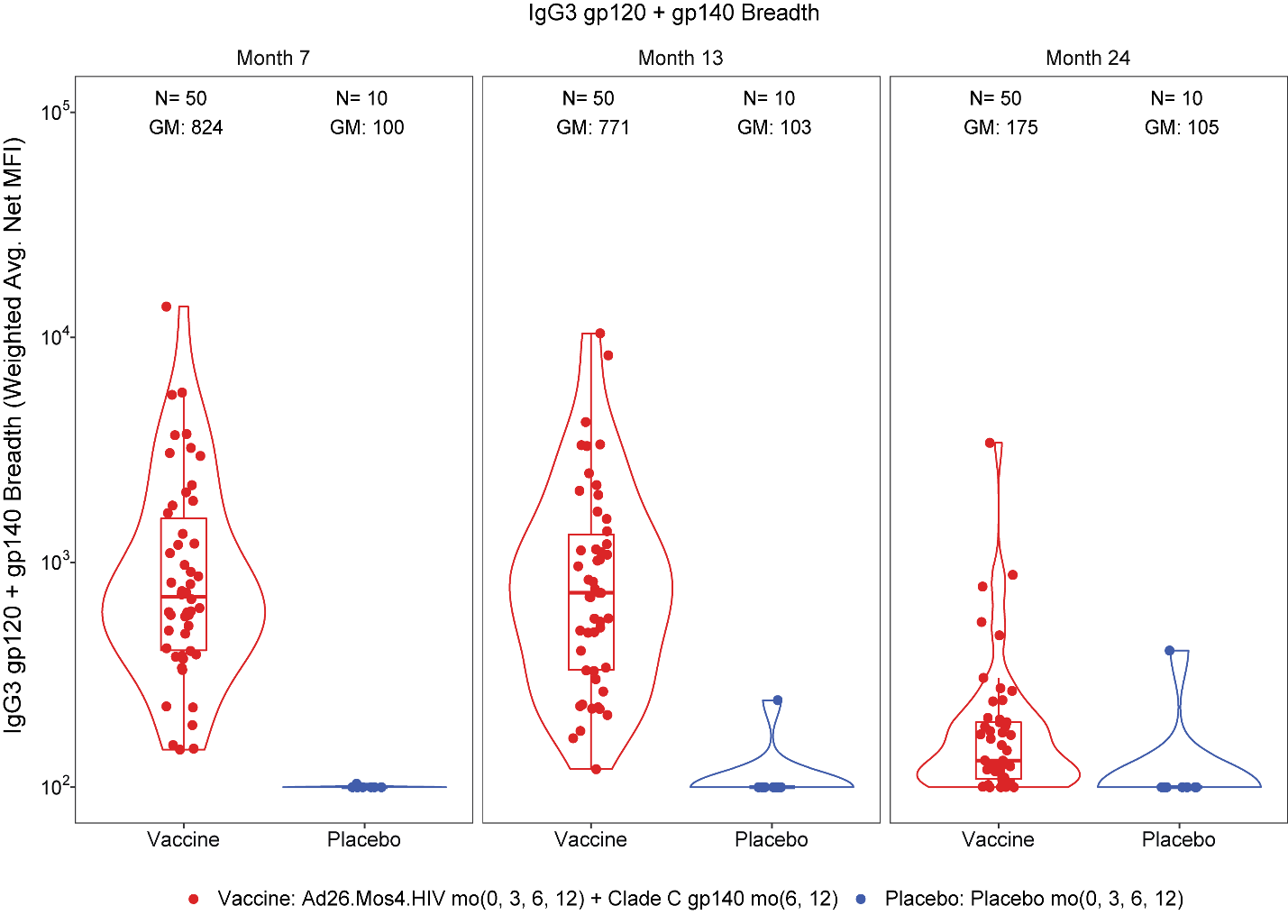


Figure S8. Distribution of ADCC AUC WITO responses at Month 7, Month 13, and Month 24, stratified by randomisation arm (vaccine vs. placebo), in the pilot immunogenicity experiments. Each violin plot contains a boxplot showing the estimated 25th, 50th, and 75th percentiles of the marker distribution, as well as a (rotated) kernel density estimate of the marker probability density function. Boxplots are based on observed response magnitudes among responders (filled colour-coded circles) and non-responders (grey triangle outlines). Panels show the actual numbers of participants with available assay data above the response rates and geometric means (GMs), with the latter two accounting for inverse-probability-of-sampling weights.


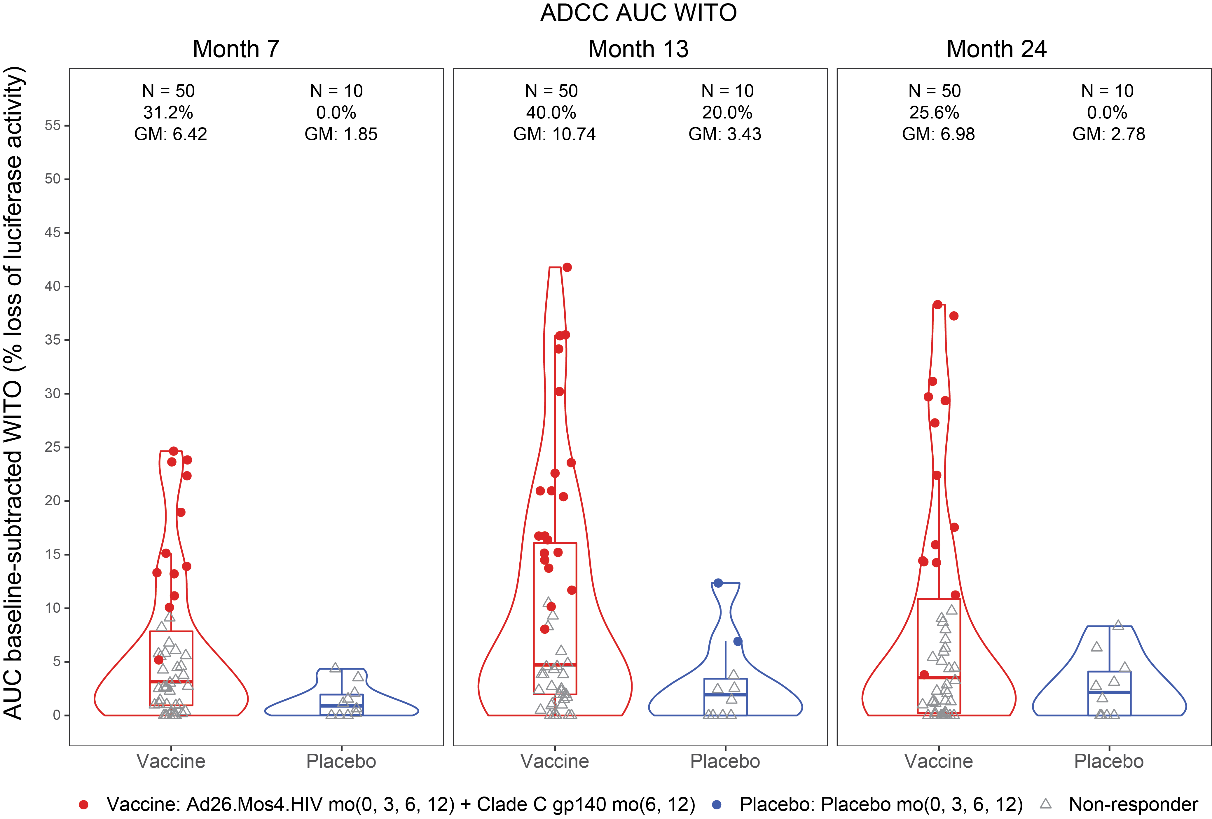


Figure S9. Distribution of ADCC AUC CAP8 responses at Month 7, Month 13, and Month 24 in the pilot immunogenicity experiments, stratified by randomisation arm (vaccine vs. placebo). Each violin plot contains a boxplot showing the estimated 25th, 50th, and 75th percentiles of the marker distribution, as well as a (rotated) kernel density estimate of the marker probability density function. Boxplots are based on observed response magnitudes among responders (filled colour-coded circles) and non-responders (grey triangle outlines). Panels show the actual numbers of participants with available assay data above the response rates and geometric means (GMs), with the latter two accounting for inverse-probability-of-sampling weights.


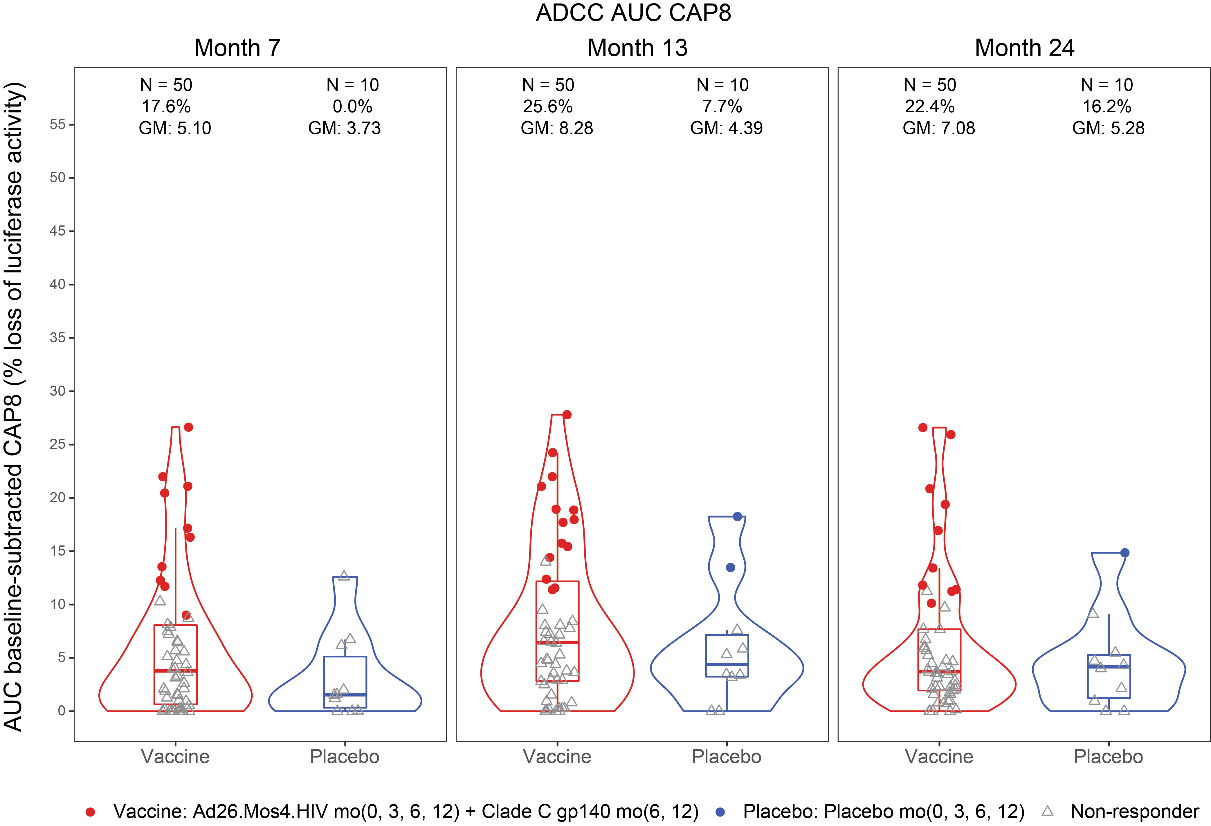


Figure S10. Distribution of ADCC AUC CH58 responses at Month 7, Month 13, and Month 24, stratified by randomisation arm (vaccine vs. placebo), in the pilot immunogenicity experiments. Each violin plot contains a boxplot showing the estimated 25th, 50th, and 75th percentiles of the marker distribution, as well as a (rotated) kernel density estimate of the marker probability density function. Boxplots are based on observed response magnitudes among responders (filled colour-coded circles) and non-responders (grey triangle outlines). Panels show the actual numbers of participants with available assay data above the response rates and geometric means (GMs), with the latter two accounting for inverse-probability-of-sampling weights.


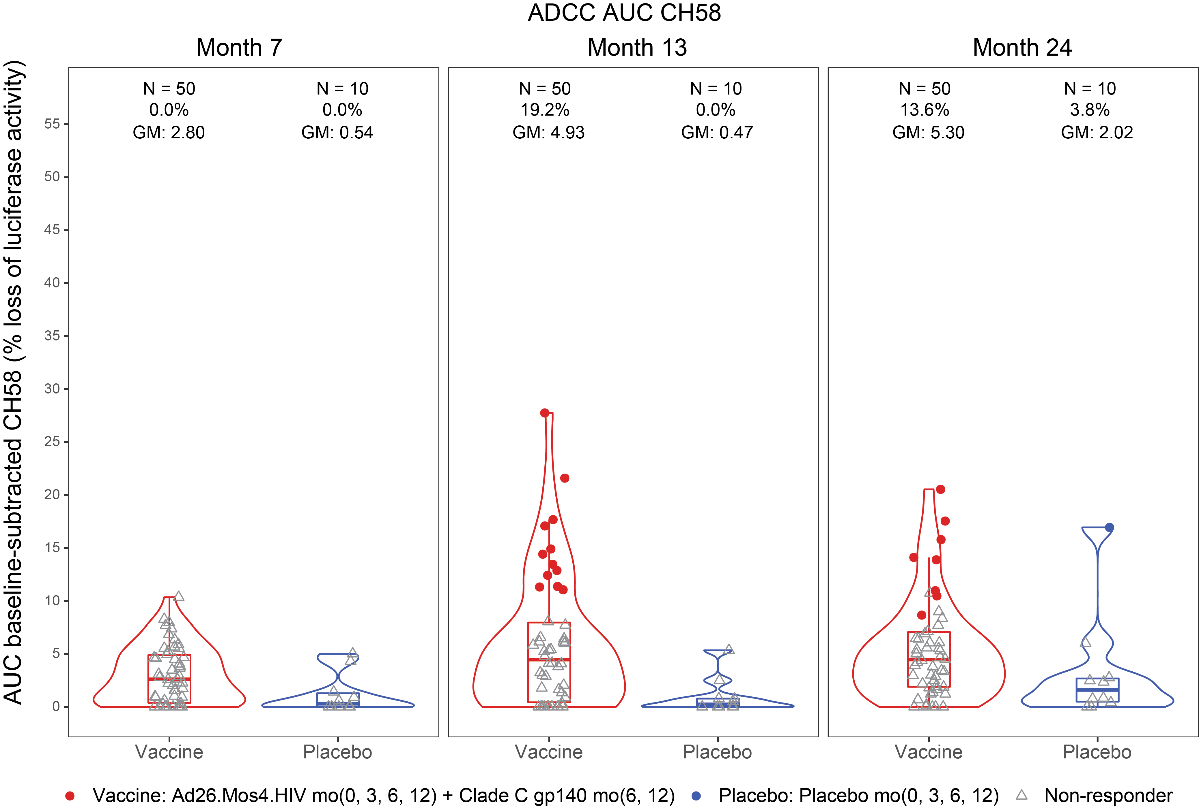


Figure S11. Distribution of CD4+ T cell IFN-γ and/or IL-2 responses to Any Env at Month 7, Month 13, and Month 24, stratified by randomisation arm (vaccine vs. placebo), in the pilot immunogenicity experiments. Each violin plot contains a boxplot showing the estimated 25th, 50th, and 75th percentiles of the CD4+ T cell IFN-γ and/or IL-2 response distribution, as well as a (rotated) kernel density estimate of the marker probability density function. Boxplots are based on observed response magnitudes among responders (red filled circles) and non-responders (grey triangle outlines). Panels show the actual numbers of participants with available assay data above the response rates and median values, with the latter two accounting for inverse-probability-of-sampling weights.


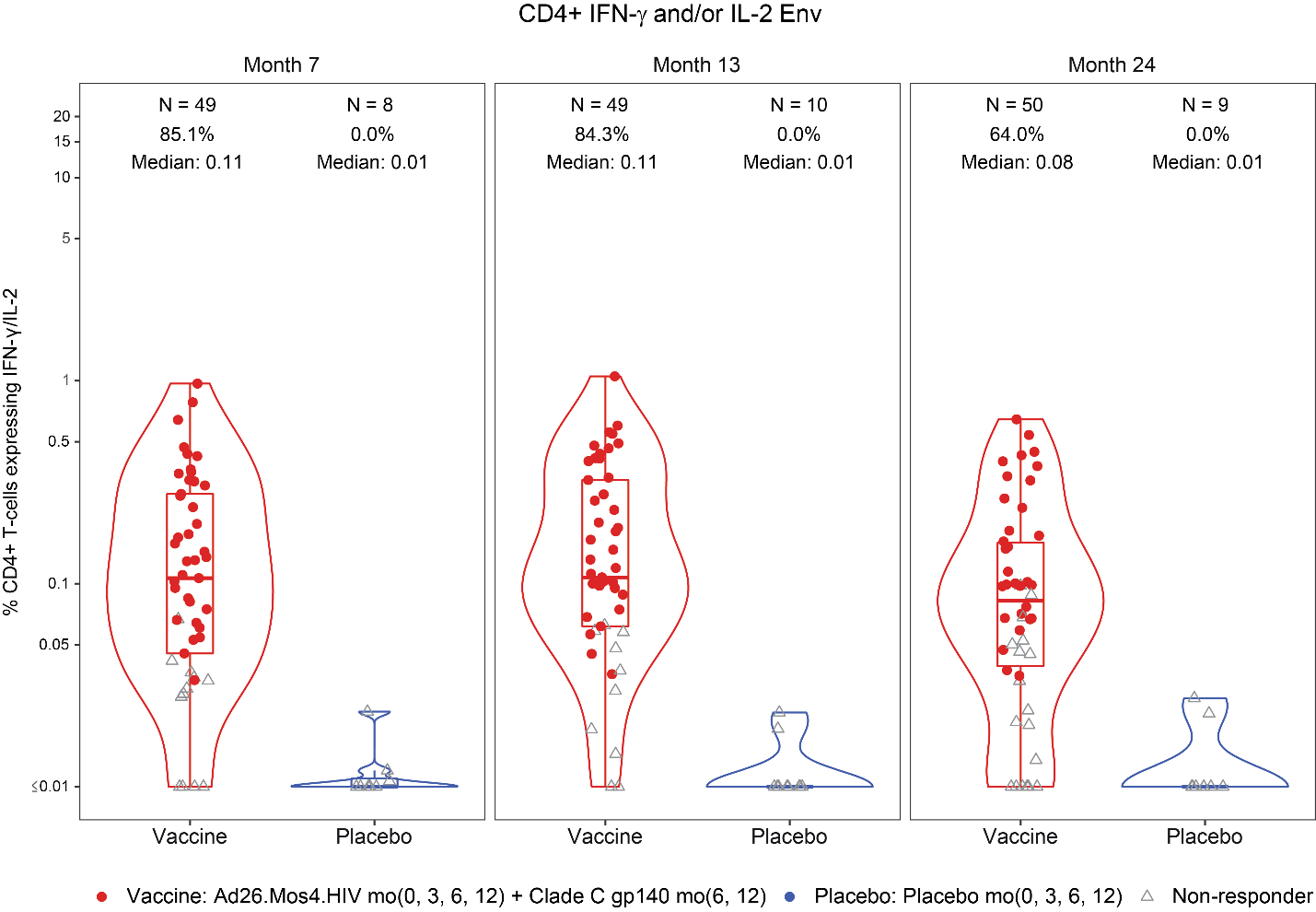


Figure S12. Distribution of CD8+ T cell IFN-γ and/IL-2 responses to Any Env at Month 7, Month 13, and Month 24, stratified by randomisation arm (vaccine vs. placebo), in the pilot immunogenicity experiments. Each violin plot contains a boxplot showing the estimated 25th, 50th, and 75th percentiles of the marker distribution, as well as a (rotated) kernel density estimate of the marker probability density function. Non-responders are shown in grey. Boxplots are based on observed response magnitudes among responders (filled colour-coded circles) and non-responders (grey triangle outlines). Panels show the actual numbers of participants with available assay data above the response rates and median values, with the latter two accounting for inverse-probability-of-sampling weights.


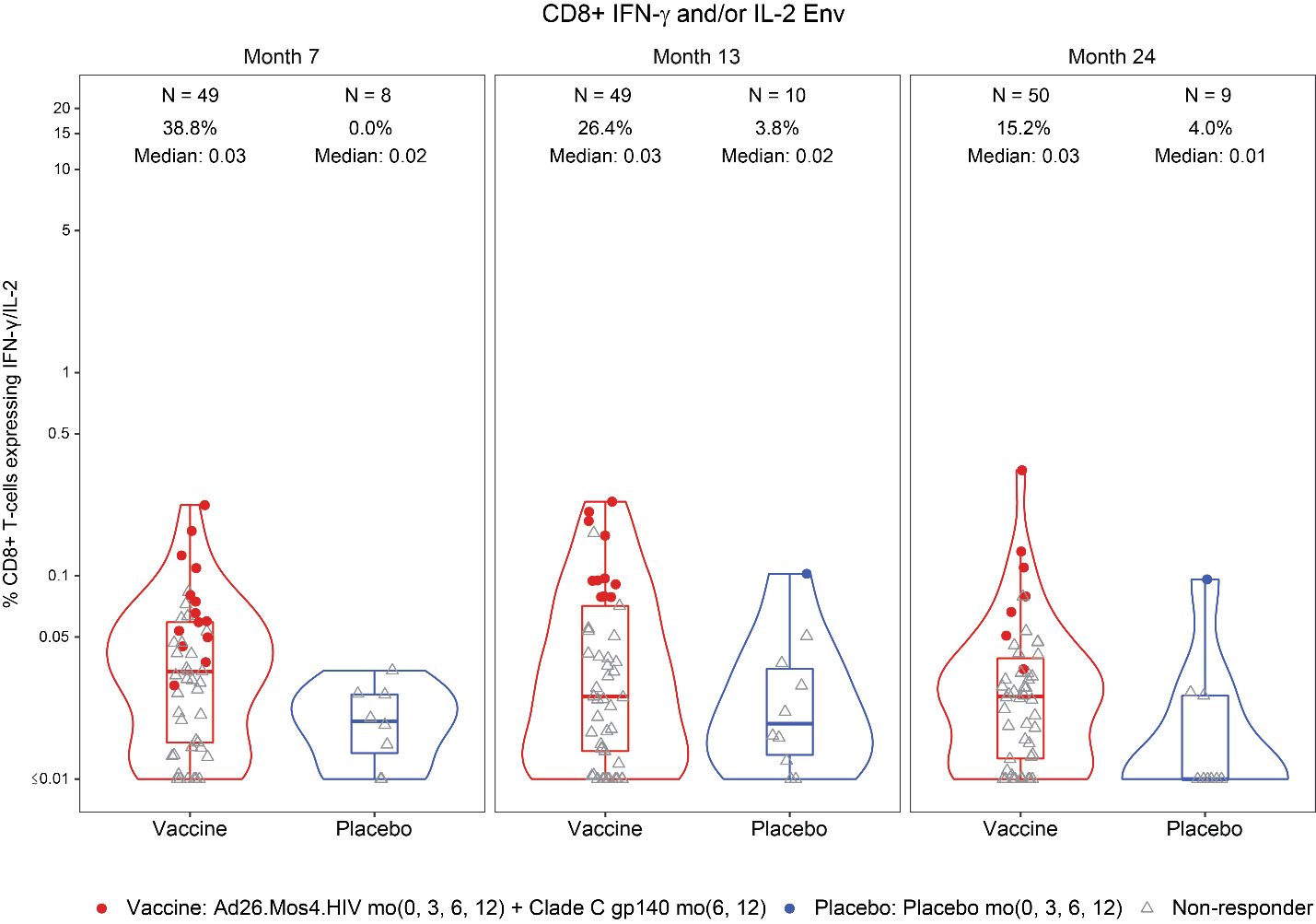


## Figure S13. Correlations of CD4+ T-cell IFN-γ and/ IL-2 response magnitudes at Month 7 between peptide pools tested in the pilot immunogenicity study. The peptide pools selected for ICS for the case control assays (designated with asterisks) were: J Mos2S gp120, J Mos2S gp41, J Mos2S RNAseInt, J Mos2S Gag, J Mos1 gp120, J Mos1 gp41.


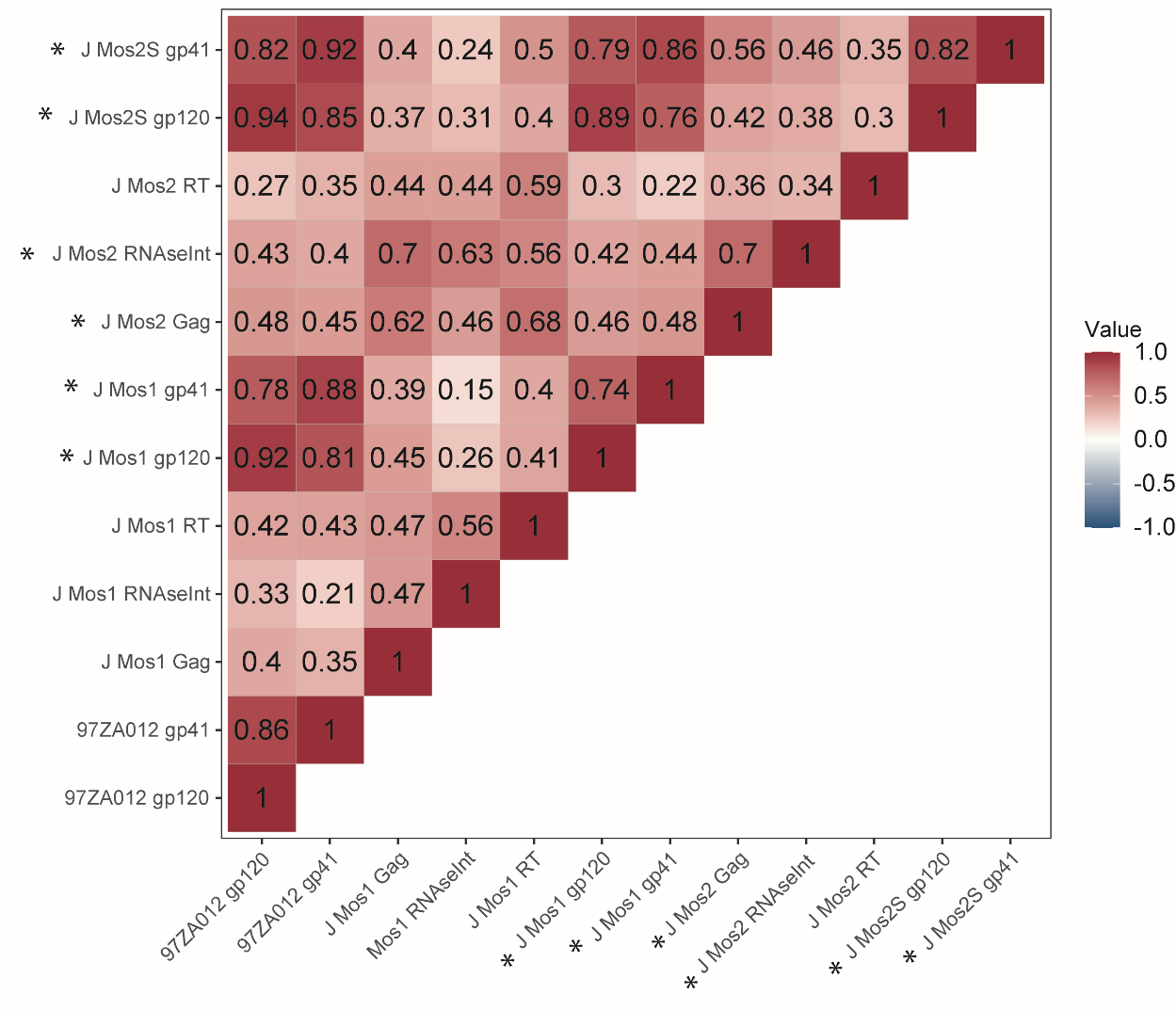


# Figure S14. Inter-correlations of the seven primary markers measured at Month 7 in the case-control subset. All numbers shown are Spearman correlation coefficients,^10^ with correlation magnitude proportional to font size.

**
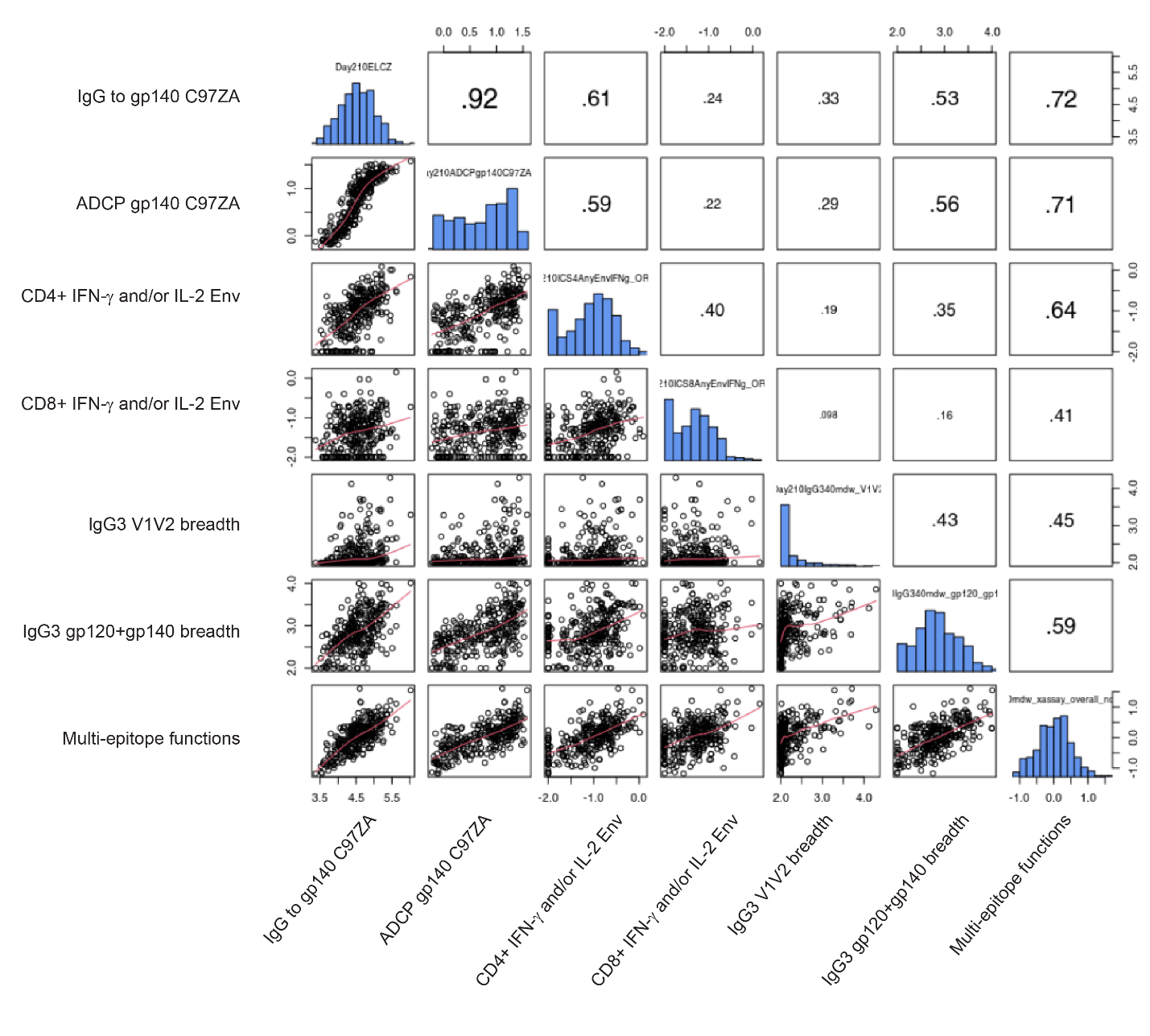
**

Figure S15. Distribution of the exploratory ELISpot PTE Env marker (measured at Month 7), stratified by treatment arm (vaccine vs. placebo), in (A) the pilot assay (only non-cases were run in the pilot assay) and (B) the case-control set (only non-cases are shown). Each violin plot contains a boxplot showing the estimated 25^th^, 50^th^, and 75^th^ percentiles of the marker distribution, as well as a (rotated) kernel density estimate of the marker probability density function. Boxplots are based on observed response magnitudes among responders (filled colour-coded circles) and non-responders (grey triangle outlines). Panels show the actual numbers of participants with available assay data above the response rates and median values, with the latter two accounting for inverse-probability-of-sampling weights.

**
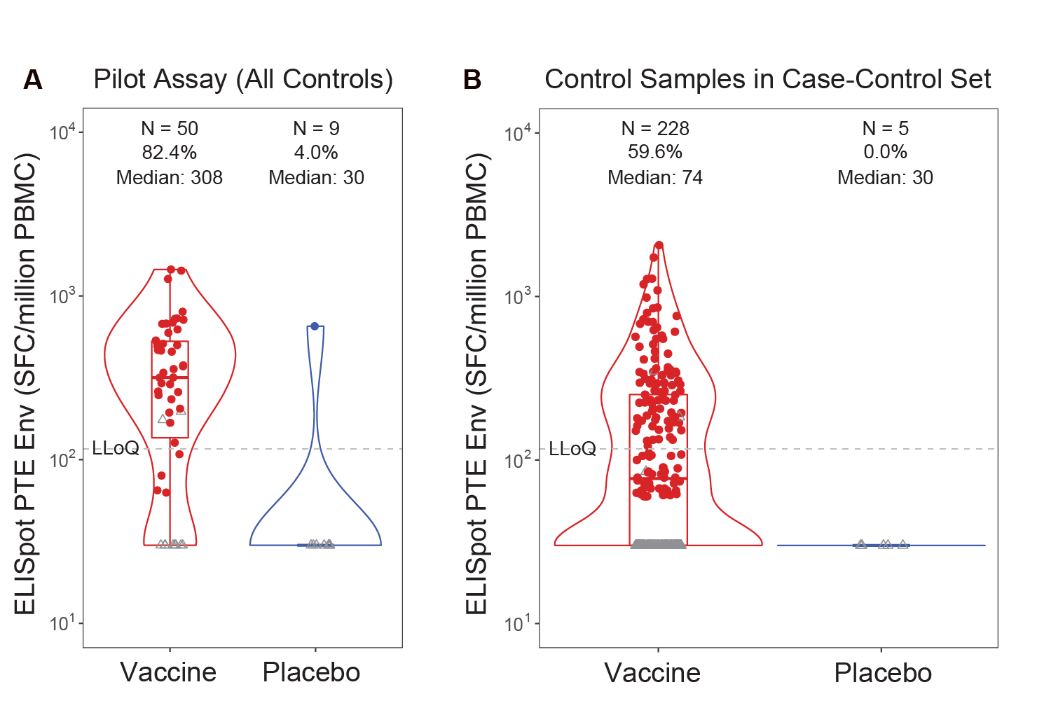
**

Table S13. Comparison of Month 7 ELISpot PTE Env marker distributions between per-protocol vaccine recipient non-cases from the pilot study vs. per-protocol vaccine recipient non-cases from the case-control study.

| **Day 210 Marker** |  | | **Per-Protocol Vaccine Recipient Non-Cases** | | | | | | | | |
| --- | --- | --- | --- | --- | --- | --- | --- | --- | --- | --- | --- |
|  | **Pilot Study** | | | | **Case-Control Study** | | |  | **Case-Control Comparison to Pilot** | | |
|  | N | Proportion with Positive Response  (95% CI) | | Geometric Mean (95% CI) | N | Proportion with Positive Response (95% CI) | Geometric Mean  (95% CI) | Positive Response Difference  (95% CI) | P-value (positive response)* | Ratio of GM  (Case-Control/  Pilot)* | P-value (GMR)* |
| ELISpot PTE Env | 50 | 80.0%  (67.0%, 88.8%) | | 240.9  (173.6, 334.2) | 228 | 60.1%  (53.6%, 66.2%) | 96.9  (83.4, 112.6) | 19.9%  (0.7%, 31.2%) | 0.0015 | 0.33 (0.22, 0.5) | < 0.001 |

*Analysis adjusted for country (South Africa vs. outside of South Africa) and BMI category (BMI <25, 25≤BMI<30, BMI ≥30) by logistic regression or linear regression. Analyses do not use inverse probability weighting, given the objective to compare the observed pilot vs. case-control control samples, whereas Figures S2-S14 use inverse probability weighting for estimating positive response rates (explaining why the positive response rates differ in Figure S14 and Table S13).

Figure S16. Distribution of four of the exploratory IgG3 V1V2 immune markers (measured at Month 7) that contributed to the IgG3 V1V2 breadth primary marker and hence also the multi-epitope functions primary marker, stratified by treatment arm (vaccine vs. placebo) and case/non-case outcome status. Markers include (**A**) IgG3 AE.A244 V1V2 Tags 293F, (**B**) IgG3 C.1086C V1V2 Tags, (**C**) IgG3 gp70-001428.2.42 V1V2, and (**D**) IgG3 gp70-1012.11.TC21.3257 V1V2. Each violin plot contains a boxplot showing the estimated 25^th^, 50^th^, and 75^th^ percentiles of the marker distribution, as well as a (rotated) kernel density estimate of the marker probability density function. Boxplots are based on observed response magnitudes among responders (filled colour-coded circles) and non-responders (grey triangle outlines). Panels show the actual numbers of participants with available assay data above the response rates and median values. The latter two accounted for inverse-probability-of-sampling weights and thus are for the population of eligible participants from which the case-control study cohort was randomly sampled.

**
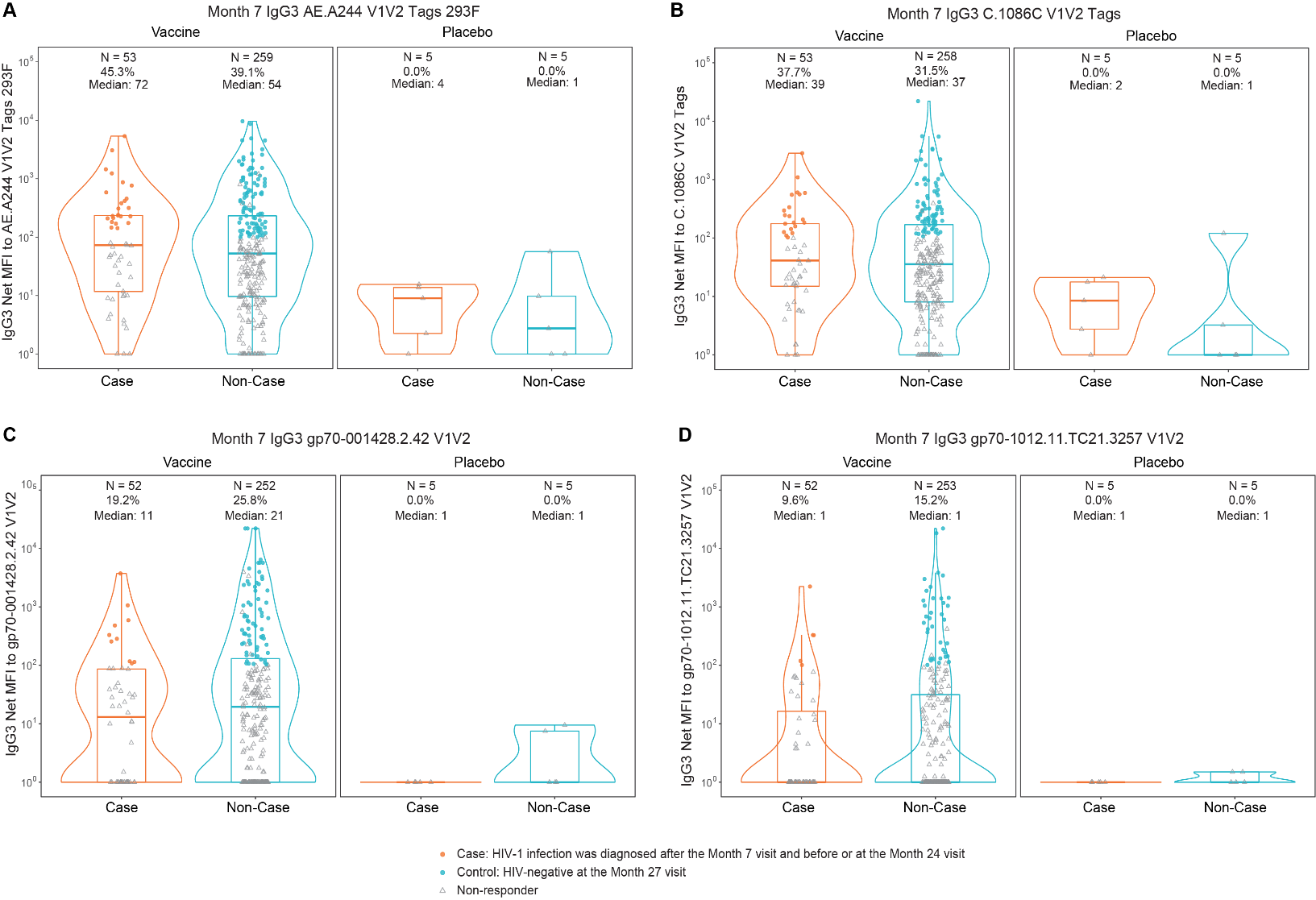
**

Figure S17. Distribution of the other four exploratory IgG3 V1V2 immune markers (measured at Month 7) that contributed to the IgG3 V1V2 breadth primary marker and hence also the multi-epitope functions primary marker, stratified by treatment arm (vaccine vs. placebo) and case/non-case outcome status. Markers include (**A**) IgG3 gp70-1394C9G1 V1V2, (**B**) IgG3 gp70-BF1266 431a V1V2, (**C**) IgG3 gp70-Ce1086 B2 V1V2, and (**D**) IgG3 gp70-B.CaseA2 V1V2Each violin plot contains a boxplot showing the estimated 25th, 50th, and 75th percentiles of the marker distribution, as well as a (rotated) kernel density estimate of the marker probability density function. Boxplots are based on observed response magnitudes among responders (filled colour-coded circles) and non-responders (grey triangle outlines). Panels show the actual numbers of participants with available assay data above the response rates and median values. The latter two accounted for inverse-probability-of-sampling weights and thus are for the population of eligible participants from which the case/control study cohort was randomly sampled.


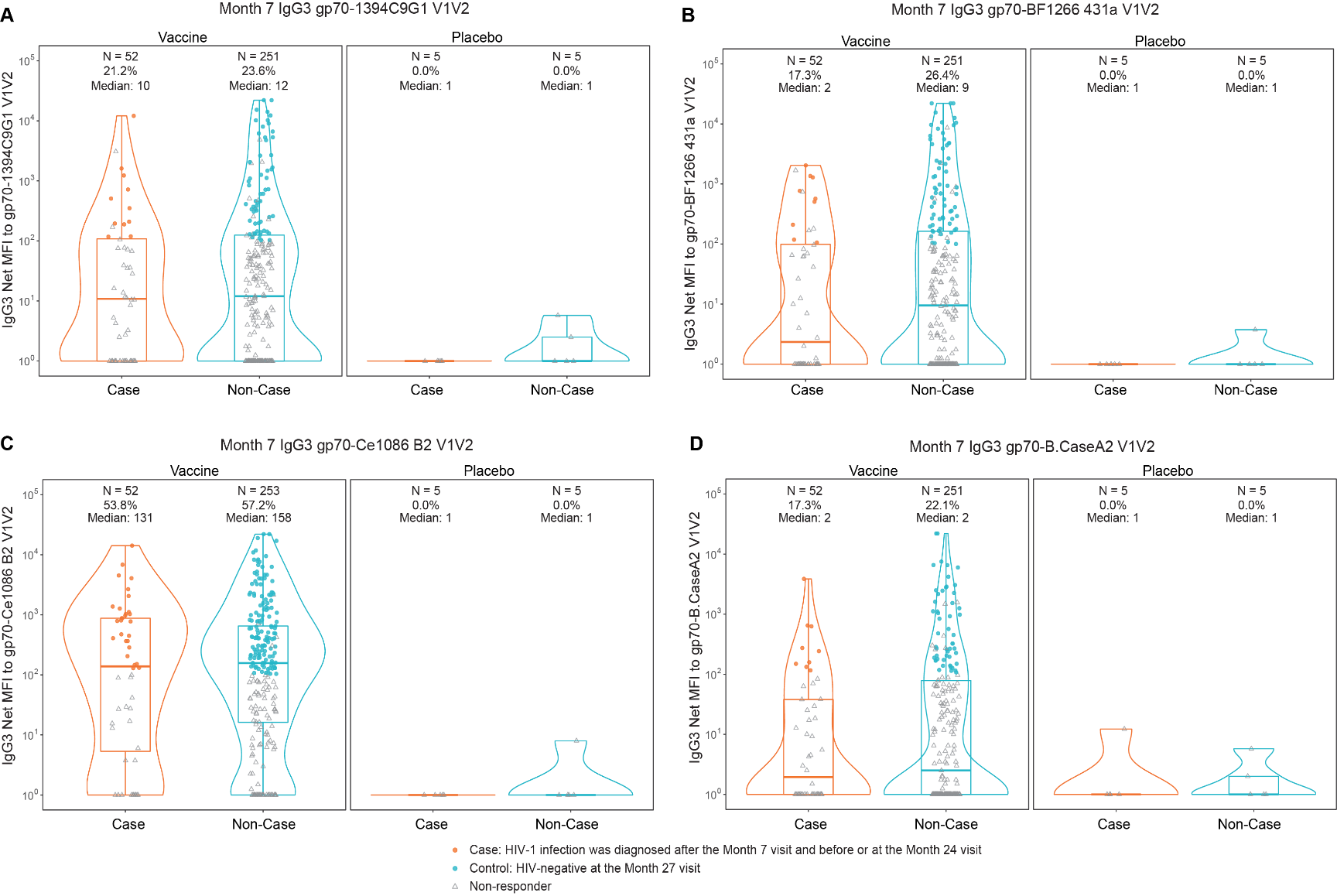


Figure S18. Distribution of the four Fc effector exploratory immune markers (measured at Month 7) that contributed to the multi-epitope functions primary marker, stratified by treatment arm (vaccine vs. placebo) and case/non-case outcome status. Markers include (A) ADCP gp140 Mos1, (B) ADCC AUC CAP8, (C) ADCC AUC CH58, and (D) ADCC AUC WITO. Each violin plot contains a boxplot showing the estimated 25th, 50th, and 75th percentiles of the marker distribution, as well as a (rotated) kernel density estimate of the marker probability density function. Boxplots are based on observed response magnitudes among responders (filled colour-coded circles) and non-responders (grey triangle outlines). Panels show the actual numbers of participants with available assay data above the response rates and median or geometric mean (GM) values. The response rates and median or GM values accounted for inverse-probability-of-sampling weights and thus are for the population of eligible participants from which the case/control study cohort was randomly sampled.

**
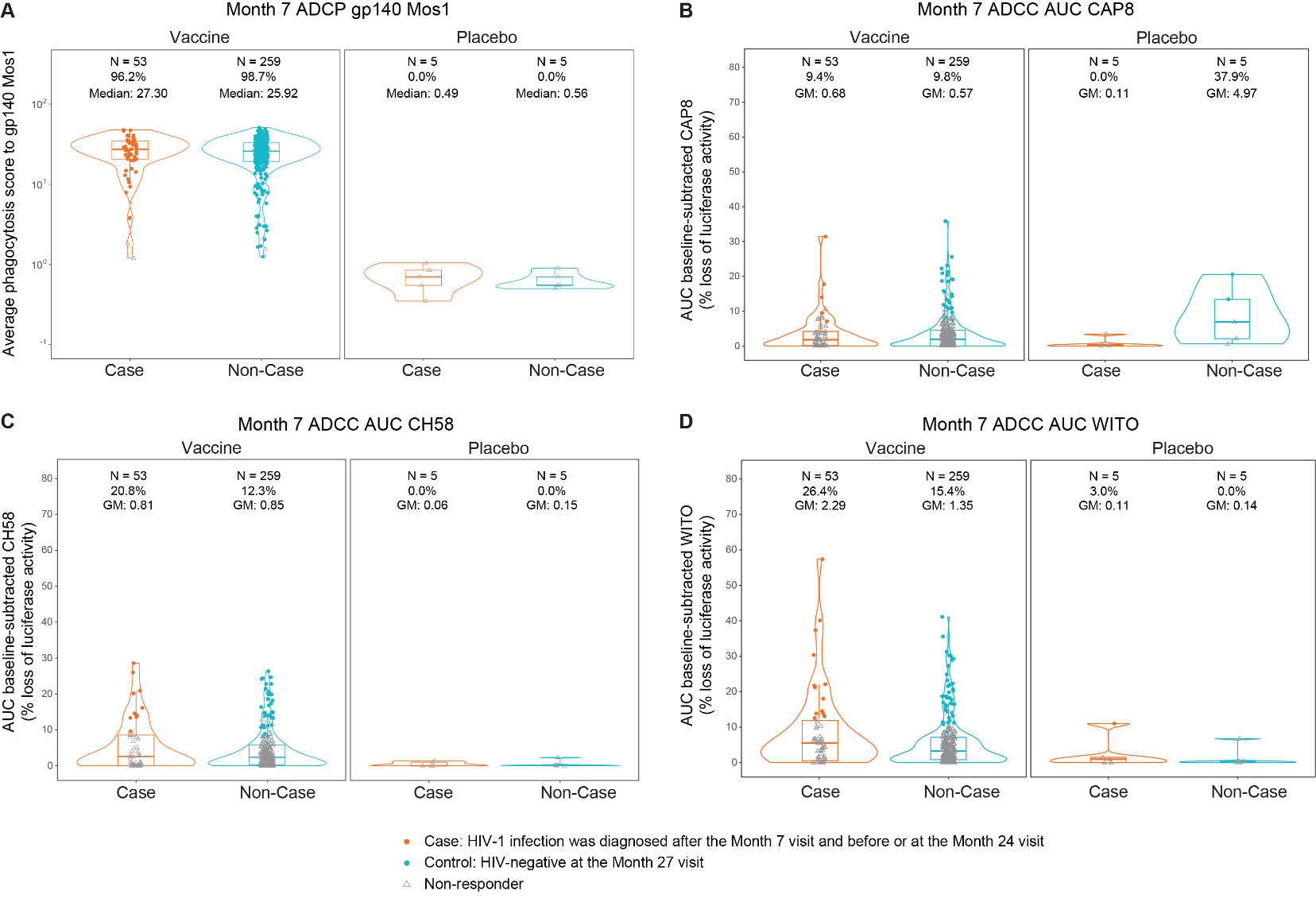
**

Figure S19. Distribution of the exploratory binding antibody breadth score immune marker (measured at Month 7) that contributed to the multi-epitope functions primary marker, stratified by treatment arm (vaccine vs. placebo) and case/non-case outcome status. The marker is IgG V1V2 breadth (same antigens as for the IgG3 V1V2 breadth primary marker). Each violin plot contains a boxplot showing the estimated 25^th^, 50^th^, and 75^th^ percentiles of the marker distribution, as well as a (rotated) kernel density estimate of the marker probability density function. Panels show the actual numbers of participants with available assay data above the geometric mean (GM) values. The latter accounted for inverse-probability-of-sampling weights and thus are for the population of eligible participants from which the case/control study cohort was randomly sampled.


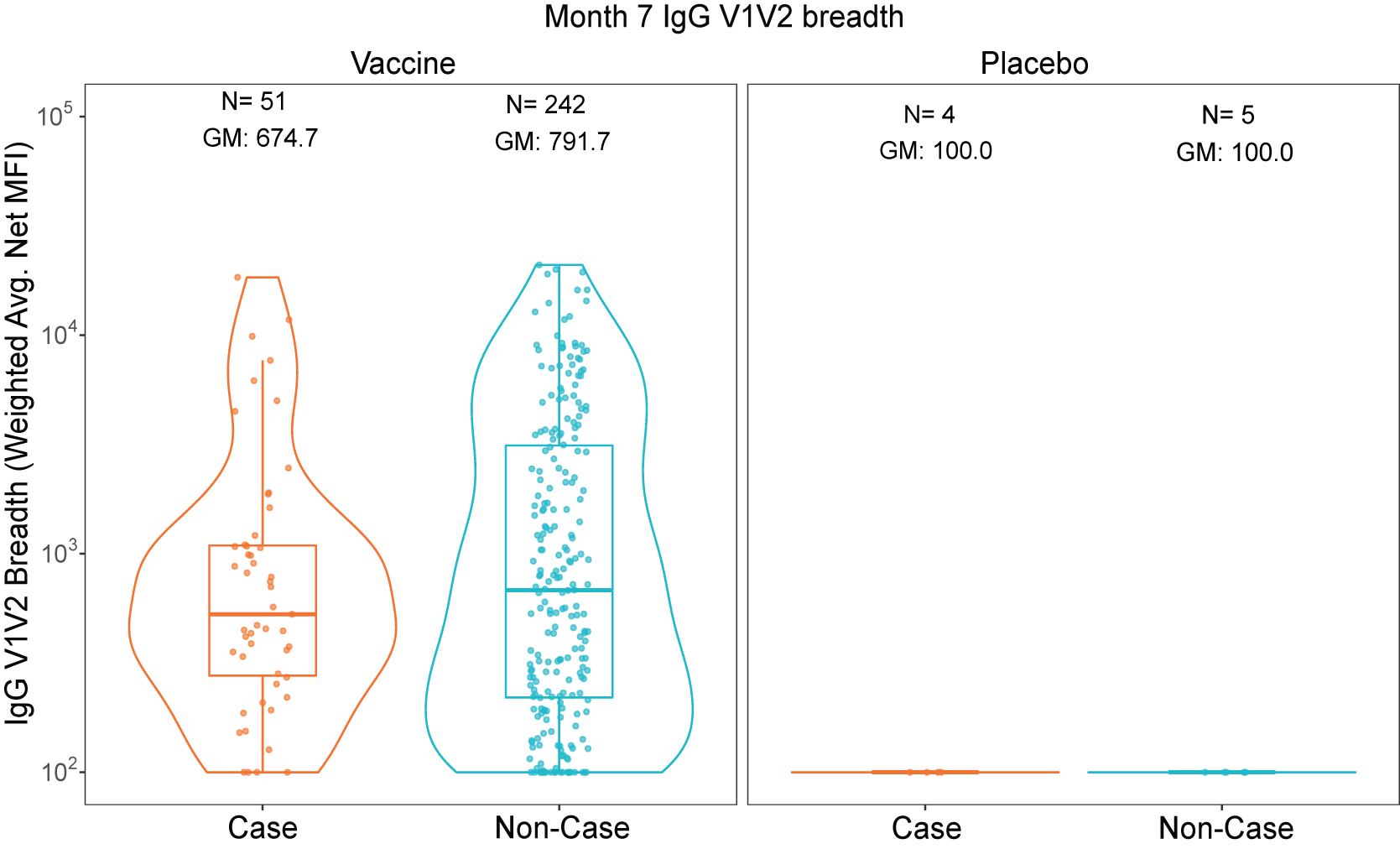


# Table S14. For each of the 15 markers at Month 7 that are included in one of the breadth primary markers or the multi-epitope functions primary marker, hazard ratio of HIV-1 in the per-protocol vaccine group per 10-fold marker increase, estimated using univariate Cox models.

^1^The V1V2 antigens are listed in descending order from most V2i tropic to most V2p tropic.

| **Marker** | **HR per 10-fold increase Pt. Est. (95% CI)** | **P-value (2-sided)** | **Mean AUC difference (average AUC against 7 V2p mAbs minus the average AUC against 4 V2i mAbs)** |
| --- | --- | --- | --- |
| **V1V2 antigens** |  |  |  |
| IgG3 gp70-001428.2.42 V1V2 | 0.80 (0.61-1.05) | 0.11 | -58142 (favors V2i) |
| IgG3 gp70-1012.11.TC21.3257 V1V2 | 0.86 (0.63-1.17) | 0.33 | -44485 (favors V2i) |
| IgG3 gp70-B.CaseA2 V1V2 | 0.86 (0.66-1.13) | 0.28 | -43851 (favors V2i) |
| IgG3 gp70-1394C9G1 V1V2 | 0.95 (0.75-1.21) | 0.70 | 34029 (favors V2p) |
| IgG3 gp70-BF1266 431a V1V2 | 0.89 (0.69-1.13) | 0.33 | 50666 (favors V2p) |
| IgG3 gp70-Ce1086 B2 V1V2 | 0.93 (0.72-1.20) | 0.58 | 53387 (favors V2p) |
| IgG3 C.1086C V1V2 Tags | 1.03 (0.77-1.38) | 0.82 | 64203 (favors V2p) |
| IgG3 AE.A244 V1V2 Tags 293F | 1.06 (0.80-1.42) | 0.67 | 82062 (favors V2p) |
| **Other markers not in IgG3 V1V2 breadth** |  |  |  |
| ADCP gp140 Mos 1 | 1.38 (0.46, 4.09) | 0.56 |  |
| IgG V1V2 breadth | 0.99 (0.65, 1.50) | 0.95 |  |
| ADCC AUC CAP8 | 1.01 (0.78, 1.30) | 0.97 |  |
| ADCC AUC CH58 | 0.99 (0.75, 1.31) | 0.96 |  |
| ADCC AUC WITO | 1.31 (0.90, 1.93) | 0.16 |  |
| CD4+ IFN-γ and/or IL-2 Env | 1.00 (0.56, 1.76) | 0.99 |  |
| CD8+ IFN-γ and/or IL-2 Env | 1.18 (0.64, 2.19) | 0.59 |  |

AUC, area under the ROC curve; mAb, monoclonal antibody.

**Figure S20. Antigenicity screening results using V2i monoclonal antibodies (mAbs) and V2p mAbs for the eight individual V1V2 antigens comprising the V1V2 breadth panel.** V2i mAbs (2158, 697D, 830A and R-1361 mAbs) and V2p mAbs (CH58, CH59, DH827, and HG107 mAbs) were titrated with V1V2 antigens to calculate AUC (area under the curve). Assays were run with total IgG detection since the tested mAbs included both IgG1 and IgG3. gp70-001428.2.42 V1V2 had strong binding for all V2i mAbs, but not all V2p mAbs. mAb, monoclonal antibody.


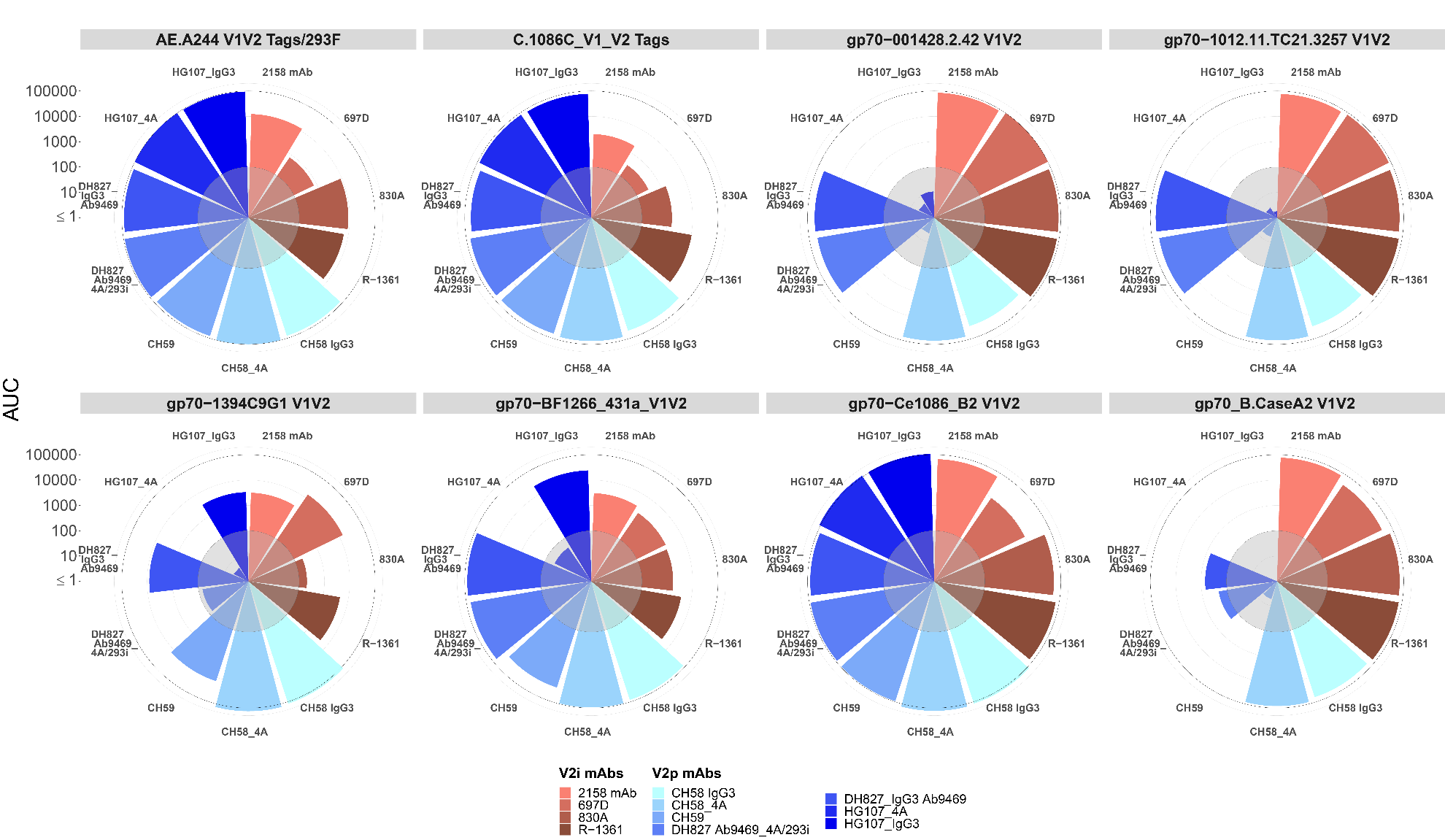


**Figure S21. Covariate-adjusted cumulative incidence of HIV-1 by Day 550 (post Month 7) for subgroups of per-protocol vaccine recipients with Month 7 marker value above a given threshold value, estimated using nonparametric threshold regression: (A) IgG gp140 C97ZA, (B) IgG3 gp120+gp140 breadth, (C) ADCP gp140 C97ZA, (D) CD4+ IFN-γ and/or IL-2 Env, (E) CD8+ IFN-γ and/or IL-2 Env, and (F) Multi-epitope functions.** The blue dots represent point estimates at each HIV-1 primary endpoint, and the black lines represent a linear interpolation of these points. The grey shaded area represents pointwise 95% confidence bands, and the upper boundary of the green shaded area represents the estimated reverse cumulative distribution function (CDF) of the marker. Estimates are cut off at the marker value at which at least 5 cases had a higher value.


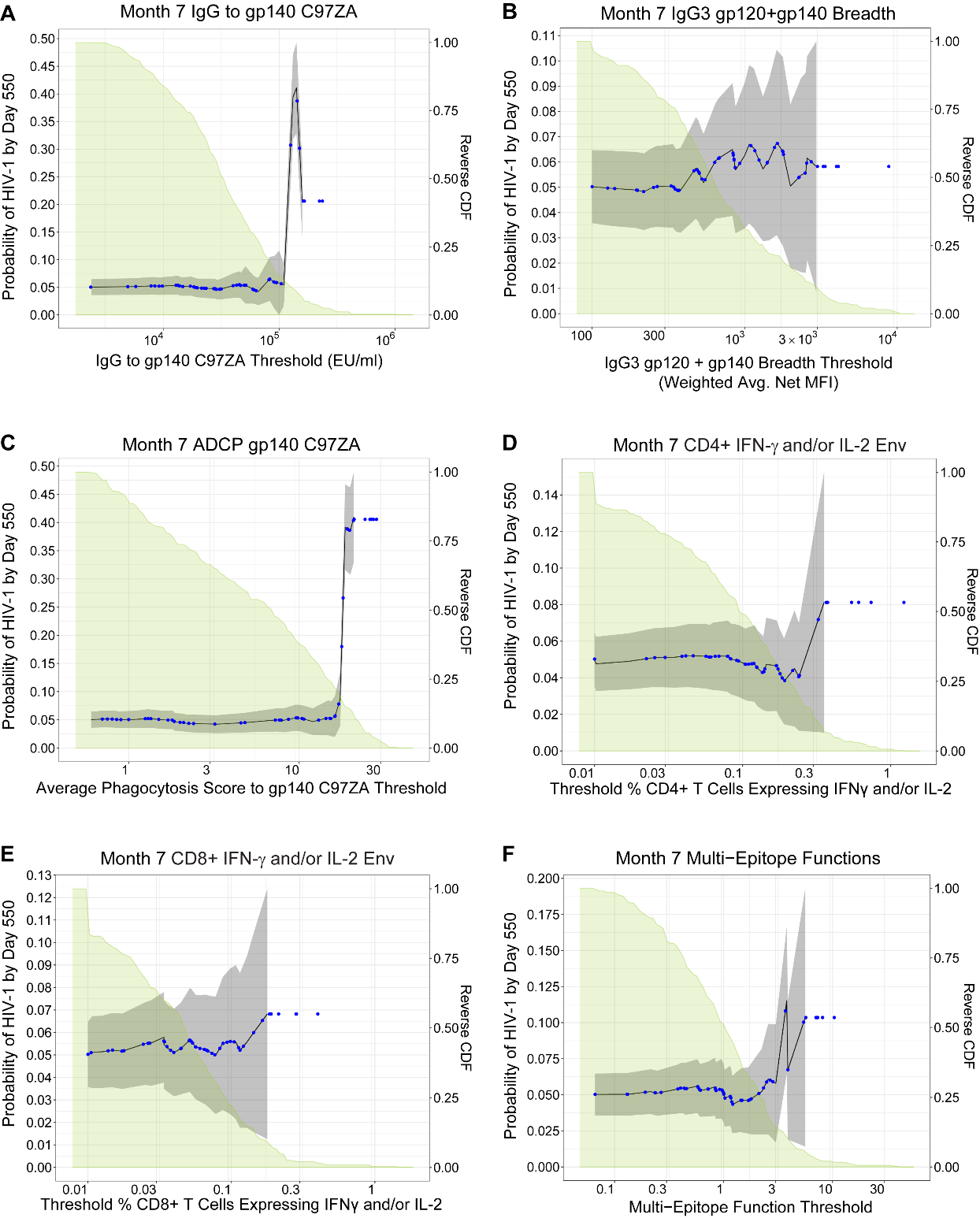


**Figure S22. Covariate-adjusted cumulative incidence of HIV-1 by day 550 (post Month 7) for subgroups of per-protocol vaccine recipients with Month 7 marker value above a given threshold value, estimated using nonparametric threshold regression: (A) IgG3 AE.A244 V1V2 Tags 293F, (B) IgG3 gp70-B.CaseA2 V1V2, (C) IgG3 gp70-Ce1086 B2 V1V2, (D) IgG3 gp70-BF1266 431a V1V2, (E) IgG3 gp70-1394C9G1 V1V2, (F) IgG3 gp70-1012.11.TC21.3257 V1V2, and (G) IgG3 C.1086C V1V2 Tags.** The blue dots represent point estimates at each HIV-1 primary endpoint, and the black lines represent a linear interpolation of these points. The grey shaded area represents pointwise 95% confidence bands, and the upper boundary of the green shaded area represents the estimated reverse cumulative distribution function (CDF) of the marker. The estimates and CIs assume a nonincreasing threshold-response function. The vertical red dashed line is the marker threshold corresponding to the largest observed HIV-1 event time (within the time frame of interest).


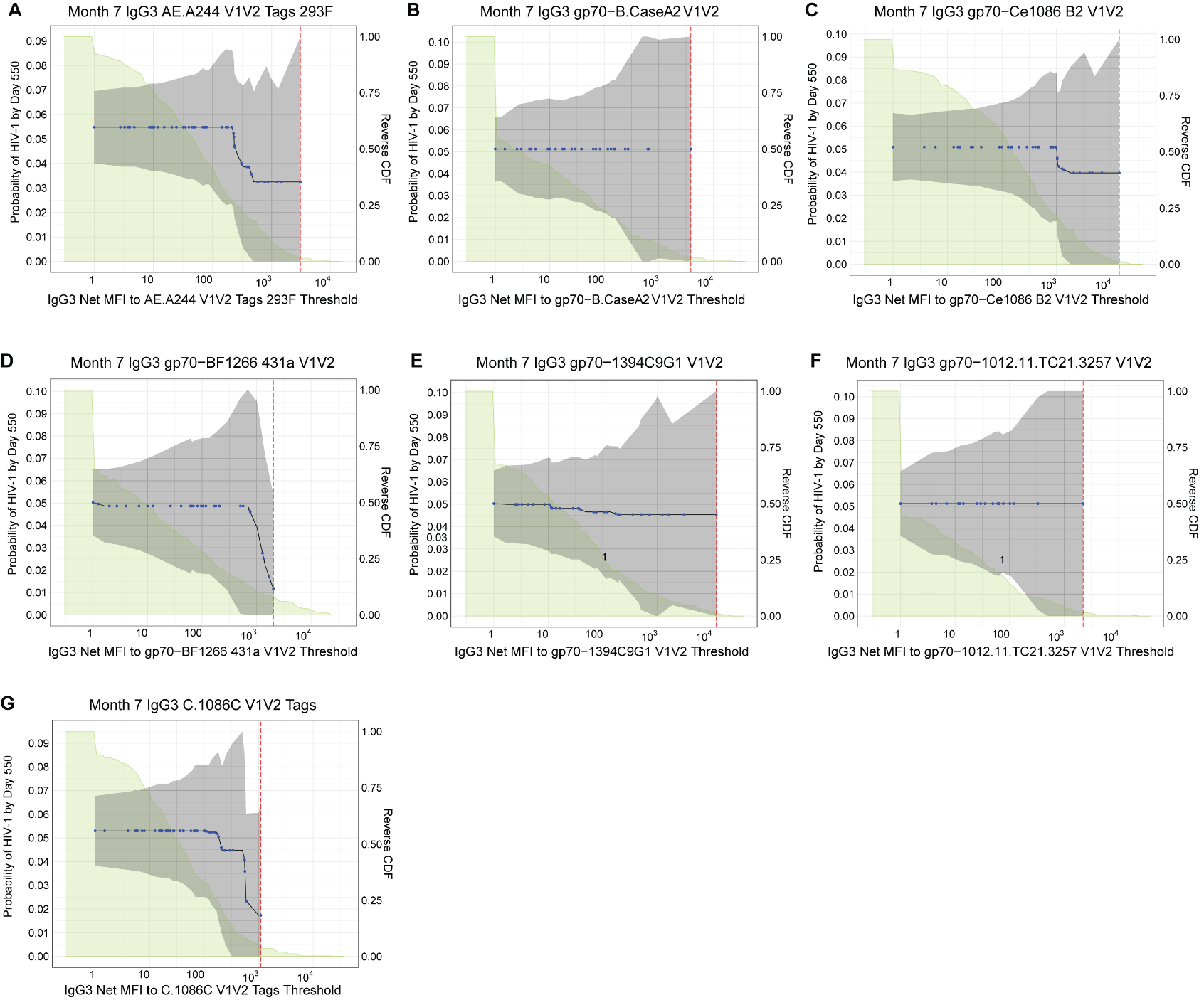


**Figure S23. Controlled vaccine efficacy (CVE) as a function of each of four exploratory IgG3 V1V2 breadth markers measured at Month 7: (A) IgG3 V1V2 breadth score trunc1, (B) IgG3 V1V2 breadth score selected trunc1, (C) IgG3 V2i breadth score trunc1, (D) IgG3 V2p breadth score trunc1, controlling for covariates.** As a sensitivity analysis, for the trunc1 markers, before deriving the MDW score, net MFIs were truncated between 1 and 22000, and nonresponder net MFIs were set to 1 (see Supplementary Methods). Estimates are computed separately using a Cox proportional hazards model (solid purple line) and a nonparametric method that assumes that the CVE curve is nondecreasing (solid blue line). Corresponding 95% pointwise confidence bands are shown in shaded blue/purple, with dotted lines representing the corresponding lower and upper limits. The distribution of the marker is plotted in orange and was done with kernel density estimation (KDE). The solid grey line represents estimated overall VE, and the dotted grey lines represent the corresponding 95% CI limits. The Cox model estimates are cut off at the 97.5^th^ quantile of the marker distribution and the nonparametric estimates are cut off at the 95^th^ quantile.

**
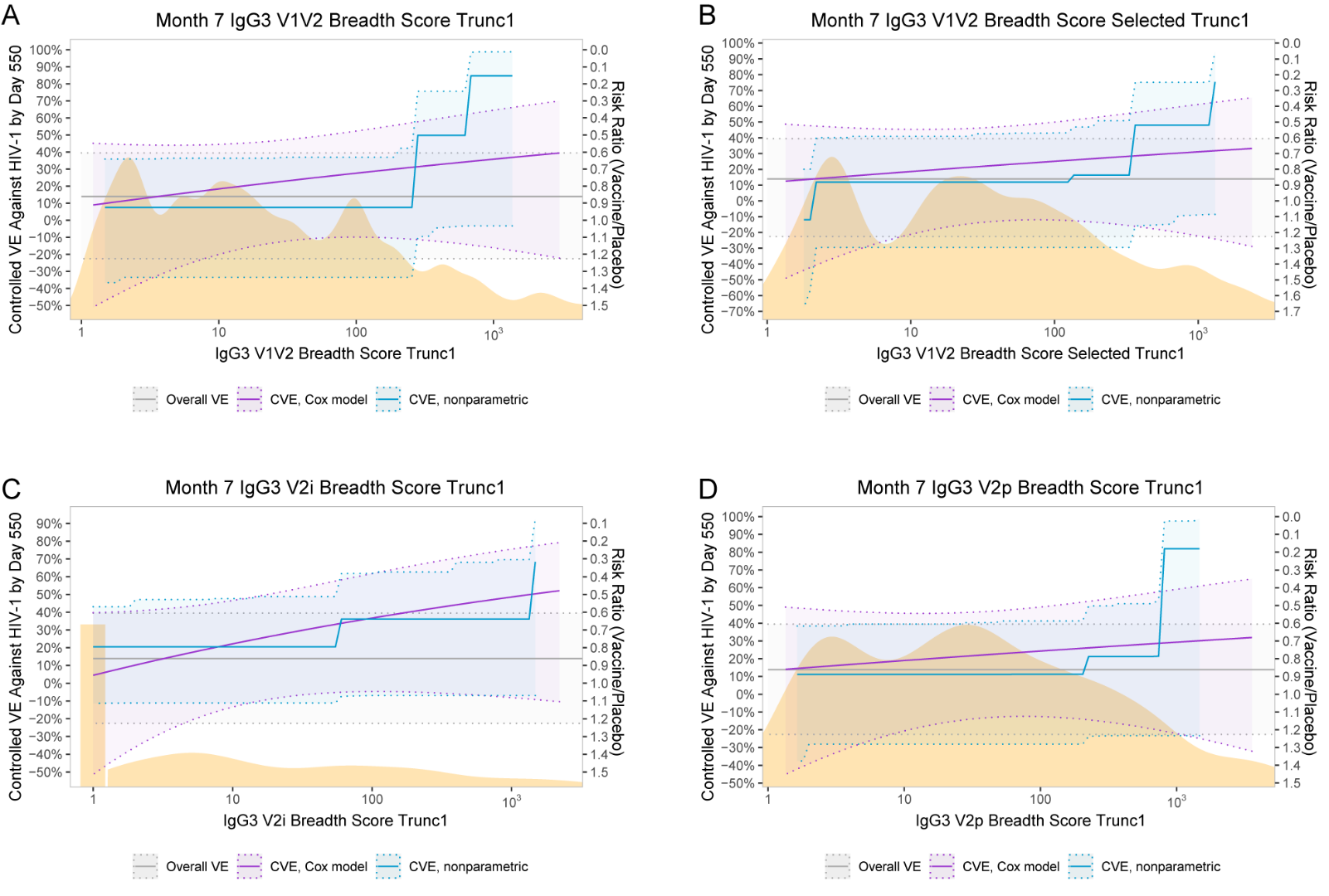
**

**Figure S24. Controlled vaccine efficacy (CVE) as a function of Month 7 (A) IgG gp140 C97ZA, (B) IgG3 gp120+gp140 breadth, (C) ADCP gp140 C97ZA, (D) CD4+ IFN-γ and/or IL-2 Env, (E) CD8+ IFN-γ and/or IL-2 Env, and (F) Multi-epitope functions, controlling for covariates.** Estimates are computed separately using a Cox proportional hazards model (solid purple line) and a nonparametric method that assumes that the CVE curve is nondecreasing (solid blue line). Corresponding 95% pointwise confidence bands are shown in shaded blue/purple, with dotted lines representing the corresponding lower and upper limits. The distribution of the marker is plotted in orange and was done with kernel density estimation (KDE), with the (possible) addition of a rectangle at the lower limit representing a point mass at the marker minimum value. The solid grey line represents estimated overall VE, and the dotted grey lines represent the corresponding 95% CI limits. The Cox model estimates are cut off at the 97.5^th^ quantile of the marker distribution and the nonparametric estimates are cut off at the 95^th^ quantile.

**
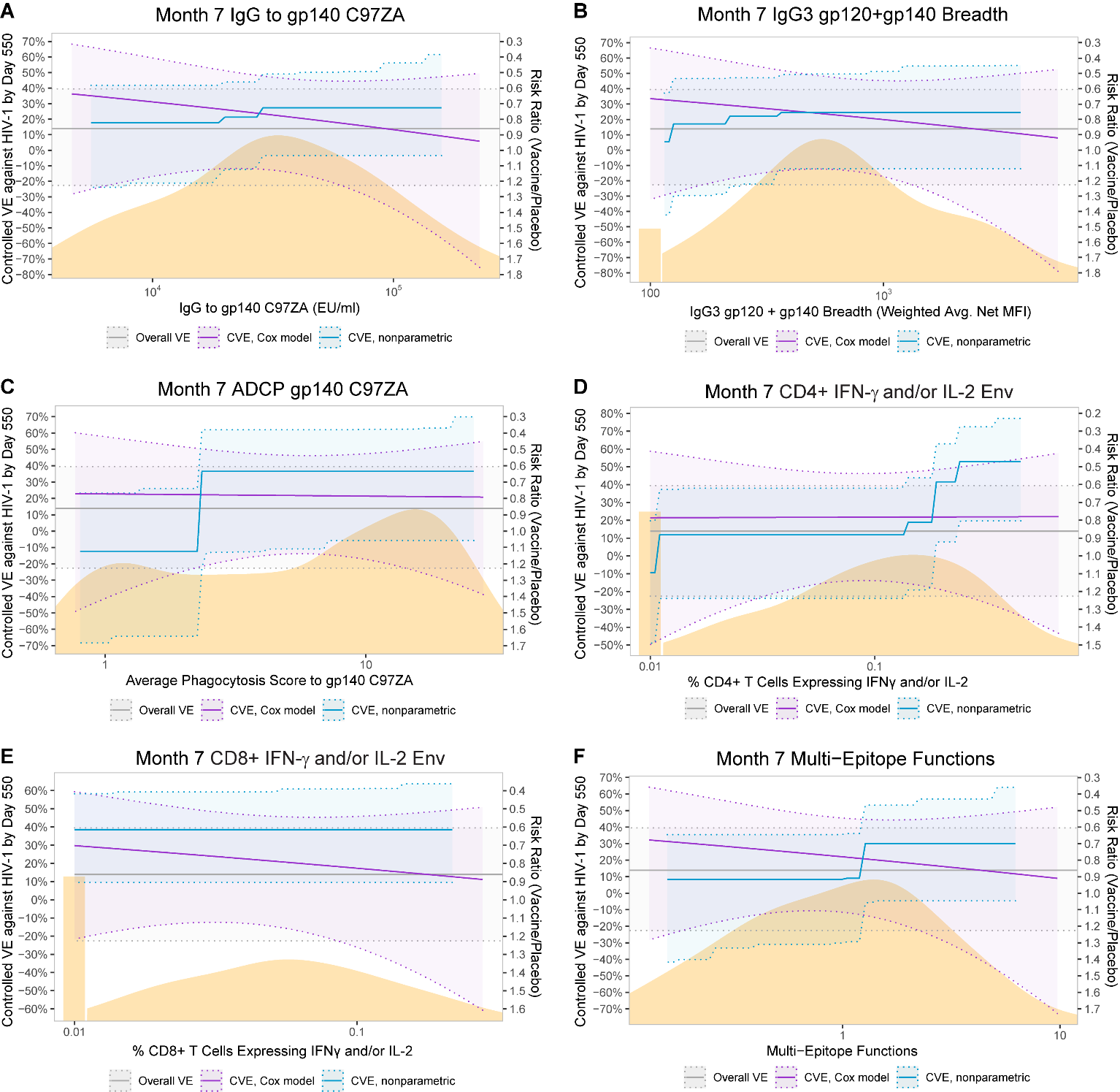
**

**Figure S25. Controlled vaccine efficacy (CVE) as a function of Month 7 (A) IgG3 AE.A244 V1V2 Tags 293F, (B) IgG3 gp70-B.CaseA2 V1V2, (C) IgG3 gp70-Ce1086 B2 V1V2, (D) IgG3 gp70-BF1266 431a V1V2, (E) IgG3 gp70-1394C9G1 V1V2, (F) IgG3 gp70-1012.11.TC21.3257 V1V2, and (G) IgG3 C.1086C V1V2 Tags.** Estimates are computed separately using a Cox proportional hazards model (solid purple line) and a nonparametric method that assumes that the CVE curve is nondecreasing (solid blue line). Corresponding 95% pointwise confidence bands are shown in shaded blue/purple, with dotted lines representing the corresponding lower and upper limits. The distribution of the marker is plotted in orange, estimated using KDE with the addition of a rectangle at the lower limit representing a point mass at the marker minimum value. The solid grey line represents estimated overall VE, and the dotted grey lines represent the corresponding 95% CI limits. The Cox model estimates are cut off at the 97.5^th^ quantile of the marker distribution and the nonparametric estimates are cut off at the 95^th^ quantile.


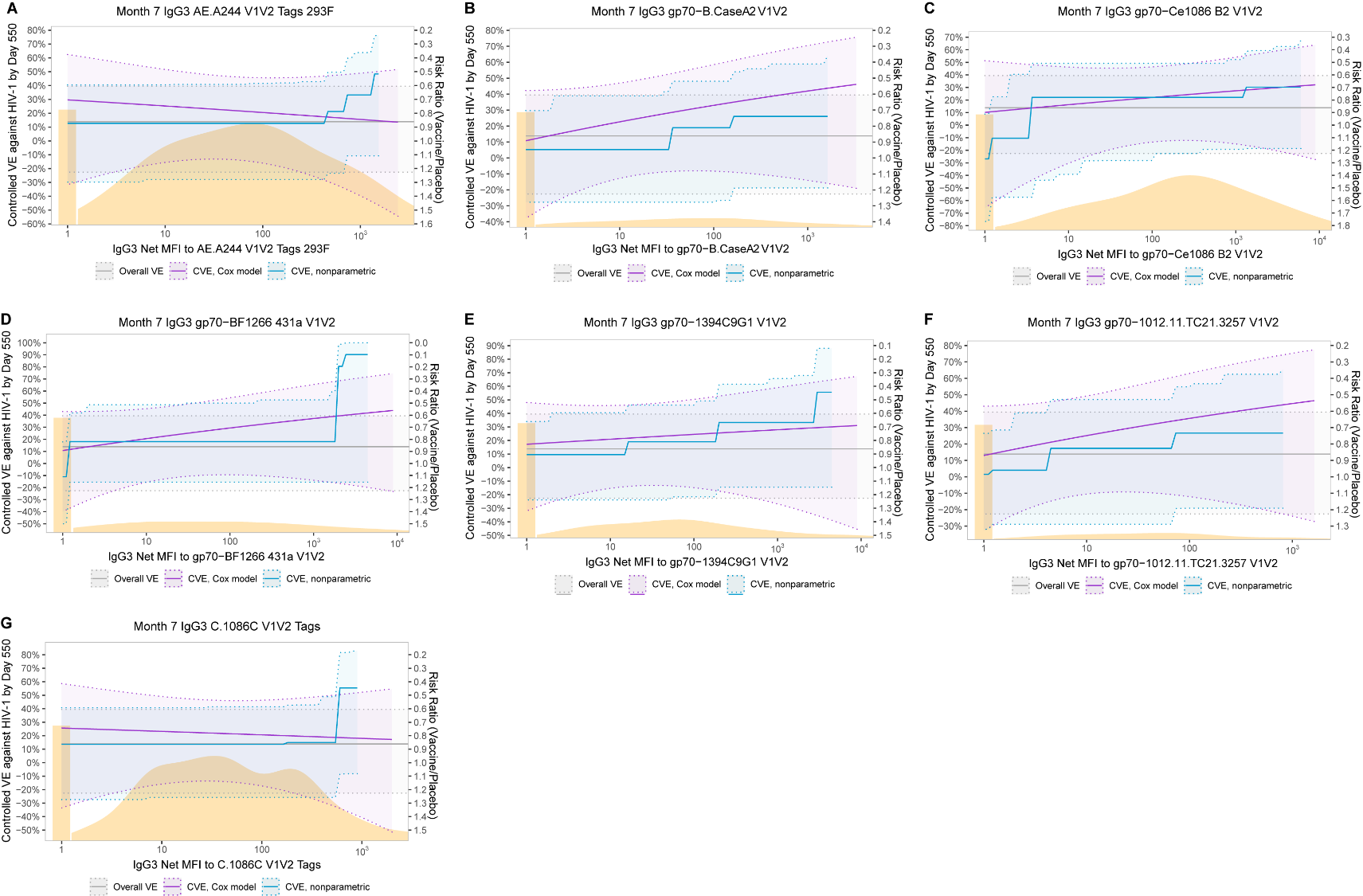


# Table S15. Comparison of Month 7 ELISA, BAMA, and ADCP response rates between baseline Ad26 serostatus among per-protocol vaccine recipients

| **Assay** | **Analyte** | **Comparison by Baseline Ad26 Serostatus^1^** | **N** | **Weighted Response Rates** | **P-value** |
| --- | --- | --- | --- | --- | --- |
| ADCP | ADCP gp140 C97ZA | Negative vs. Positive | 47 vs. 265 | 93.4% vs. 92.3% | 0.846 |
|  | ADCP gp140 Mos1 | Negative vs. Positive | 47 vs. 265 | 100.0% vs. 98.2% | 0.323 |
| BAMA | IgG3 gp70-001428.2.42 V1V2 | Negative vs. Positive | 46 vs. 258 | 22.5% vs. 25.8% | 0.668 |
| ELISA | IgG to gp140 C97ZA | Negative vs. Positive | 47 vs. 263 | 100.0% vs. 100.0% | ± |
|  | IgG gp140 Mos1 | Negative vs. Positive | 47 vs. 263 | 100.0% vs. 100.0% | ± |

^1^Positive = Ad26 neutralisation titre (90% inhibitory concentration [IC90]) of Month 0 serum sample greater than the lower limit of quantitation (LLOQ) (LLOQ = 17); Negative = Ad26 neutralisation titre (IC90) of Month 0 serum was at or below the lower limit of quantitation.

Positive response was not calculated for BAMA magnitude breadth, so no comparisons were performed.

Both ELISA analytes had 100% response rates, so P values were not calculated.

Weighted response rates were calculated with inverse probability of sampling weights, so estimates and inferences are for the population of eligible participants from which the case/control set was selected. Observed response rates are unweighted.

The chi-squared test was used for comparing the comparing 2 x 2 contingency table of weighted response rates accounting for the two-phase sampling. Statistical tests are all based on 2-sided α = 0.05. Multiplicity adjustment was not performed on the collection of P-values.

**Supplementary References**

1. Yates NL, deCamp AC, Korber BT, et al. HIV-1 Envelope Glycoproteins from Diverse Clades Differentiate Antibody Responses and Durability among Vaccinees. *J Virol* 2018; **92**(8).

2. Price MN, Dehal PS, Arkin AP. FastTree: computing large minimum evolution trees with profiles instead of a distance matrix. *Mol Biol Evol* 2009; **26**(7): 1641-50.

3. Price MN, Dehal PS, Arkin AP. FastTree 2--approximately maximum-likelihood trees for large alignments. *PLoS One* 2010; **5**(3): e9490.

4. Liao HX, Bonsignori M, Alam SM, et al. Vaccine induction of antibodies against a structurally heterogeneous site of immune pressure within HIV-1 envelope protein variable regions 1 and 2. *Immunity* 2013; **38**(1): 176-86.

5. Li F, Malhotra U, Gilbert PB, et al. Peptide selection for human immunodeficiency virus type 1 CTL-based vaccine evaluation. *Vaccine* 2006; **24**(47-48): 6893-904.

6. Horton H, Thomas EP, Stucky JA, et al. Optimization and validation of an 8-color intracellular cytokine staining (ICS) assay to quantify antigen-specific T cells induced by vaccination. *J Immunol Methods* 2007; **323**(1): 39-54.

7. Fisher L, Zinter M, Stanfield-Oakley S, et al. Vaccine-Induced Antibodies Mediate Higher Antibody-Dependent Cellular Cytotoxicity After Interleukin-15 Pretreatment of Natural Killer Effector Cells. *Front Immunol* 2019; **10**: 2741.

8. Trkola A, Matthews J, Gordon C, Ketas T, Moore JP. A cell line-based neutralization assay for primary human immunodeficiency virus type 1 isolates that use either the CCR5 or the CXCR4 coreceptor. *J Virol* 1999; **73**(11): 8966-74.

9. Mielke D, Stanfield-Oakley S, Borate B, et al. Selection of HIV Envelope Strains for Standardized Assessments of Vaccine-Elicited Antibody-Dependent Cellular Cytotoxicity-Mediating Antibodies. *J Virol* 2022; **96**(2): e0164321.

10. Liu Q, Li C, Wanga V, Shepherd BE. Covariate-adjusted Spearman's rank correlation with probability-scale residuals. *Biometrics* 2018; **74**(2): 595-605.
