# Supplementary material for: Immune correlates analysis of the Imbokodo (HVTN 705/HPX2008) efficacy trial of a mosaic HIV-1 vaccine regimen evaluated in Southern African people assigned female sex at birth: a two-phase case-control study
Source: eBioMedicine. 2024 Sep 4;108:105320. doi: 10.1016/j.ebiom.2024.105320 (PMC11404224; doi:10.1016/j.ebiom.2024.105320)
Supplement: REDACTED_SAP-FD-VAC89220HPX2008-1251709 [file mmc2.pdf]

Statistical Analysis Plan for Assessing Correlates in HVTN 705/VAC89220HPX2008: Month 7 Marker Analyses

Version 12.0

PPD

PPD

PPD

Fred Hutchinson Cancer Center and Janssen Statistics

STATISTICAL ANALYSIS PLAN

HVTN 705/VAC89220HPX2008

A multicenter, randomized, double-blind, placebo-controlled phase 2b efficacy study of a heterologous prime/boost vaccine regimen of Ad26.Mos4.HIV and aluminum phosphate-adjuvanted Clade C gp140 in preventing HIV-1 infection in women in sub-Saharan Africa

Version: 12.0

STATISTICAL ANALYSIS PLAN

**Protocol Name:**	A multicenter, randomized, double-blind, placebo-controlled phase 2b efficacy study of a heterologous prime/boost vaccine regimen of Ad26.Mos4.HIV and aluminum phosphate-adjuvanted Clade C gp140 in preventing HIV-1 infection in women in sub-Saharan Africa
**Protocol Number:**	HVTN 705/VAC89220HPX2008
**Author(s):**	PPD [redacted]
**Version:**	12.0

Contents

1 Updates in the v12 SAP 9

2 Updates in the v11 SAP 9

3 Updates in the v10 SAP 10

4 Updates in the v9 SAP 10

5 Updates to the v8 SAP 12

6 Updates in this v7 SAP 14

7 Updates to v6 SAP 15

8 Updates to v5 SAP 16

9 Updates to v4 SAP 17

10 Updates to v3 SAP 17

11 Updates to v2 SAP 19

12 Updates to v1 SAP 19

13 Correlates Study Objectives from the Protocol 20

14 Re-structuring the Correlates Objectives 21

14.1 Host Variables (Correlates Objectives) 21

14.2 HIV-1 Pathogen Variables (Sieve Analysis Objectives) 22

14.3 Host and HIV-1 Pathogen Variables (Integrated Correlates and Sieve Analysis Objectives) 22

14.4 Scope of this Correlates SAP 23

15 Cohorts Relevant for the Assessment of Correlates 24

15.1 Cohort Definitions 24

15.2 Two-phase case-control sampling design 25

16 Baseline and Month 7 Immune Marker Variables for CoR and CoP Analyses 26

16.1 Month 7 Markers 26

16.2 Rationale for the 7 primary immune markers 31

16.3 Baseline Markers 39

17 Summary of the Approach to the Data Analyses 39

17.1 Analysis of Objective 3: Month 7 Individual Marker CoRs 40

17.1.1 Analysis of Objective 3: Month 7 Multivariable Primary Marker CoRs 40

17.2 Analysis of Objective 5: Month 7 Individual Marker Controlled VE CoPs . . 40

17.3 Analysis of Objective 6: Month 7 Individual Marker Mediators of VE 41

18 Details of the Data Analyses 41

18.1 Analysis of Objective 3: Month 7 Individual Marker CoRs 41

18.1.1 Inverse probability sampling weights used in CoR analyses 41

18.1.2 Choice of regression methods 42

18.1.3 Univariate CoR: Nonparametric threshold regression modeling 44

18.1.4 Univariable CoR: Supportive Exploratory Flexible Parametric Risk Modeling 45

18.1.5 P-values and Multiple hypothesis testing adjustment for CoR analysis 45

18.1.6 Analysis of Objective 3: Month 7 Primary Multivariable Marker CoRs 46

18.1.7 Exploratory Analysis of Objective 3: Month 7 Multivariable Primary Marker CoRs 46

18.2 Analysis of Objective 5: Month 7 Individual Marker Controlled VE CoPs . . 46

18.3 Analysis of Objective 6: Month 7 Individual Marker Mediators of VE 51

19 Handling missing data 53

20 Plan for determining the dilution of the ADCP for defining the ADCP biomarker 54

20.1 Notes on the ADCP biomarkers 54

20.2 Notes on the ELISpot PTE Env marker 55

References 56

21 Appendix: Notes for Statisticians Implementing the Immune Correlates Code Suite 57

List of Tables

1 Baseline Subgroups that are Analyzed for Cox Modeling Correlates of Risk. . 44

2 Learning Algorithms in the super learner Library for mediation methods[1]. . 53

List of Figures

1 Updates in the v12 SAP

Based on evidence of degradation of samples in the case-control sample set assayed by the ELISpot PTE Env primary marker, this ELISpot PTE Env marker was omitted from the set of markers included in correlates of risk and correlates of protection analyses (additional details in Section 16.2). Descriptive plots of the ELISpot PTE Env primary marker are included. Because of the importance of including T-cell marker measurements in the set of primary markers evaluated as correlates of risk and as correlates of protection, the two ICS markers that are in the Multi-epitope functions primary marker (ICS CD4 ANY-ENV and ICS CD8 ANY-ENV) are now analyzed as primary markers (previous versions of the SAP analyzed these markers as non-primary markers). ICS CD4 is the percentage of CD4+ T cells expressing IFN-gamma and/or IL-2 against the ANY-ENV antigen, and ICS CD8 is the percentage of CD8+ T cells expressing IFN-gamma and/or IL-2 against the ANY-ENV antigen. In addition, because of the removal of the ELISpot marker from the correlates analyses, the multi-epitope functions primary marker was recalculated to no longer include the ELISpot marker as one of the constituent variables. The new multi-epitope functions marker is

- mdw_xassay_overall_noelispot

2 Updates in the v11 SAP

The v11 SAP adds 4 exploratory markers, for conduct of univariate correlates of risk and protection analyses. These markers are

- IgG340mdw_V1V2_trunc1
- mdw_xassay_select_igg3v1v2_trunc1
- IgG340mdw_V2i_trunc1
- IgG340mdw_V2p_trunc1

These 4 maximal diversity weighted (MDW) breadth score markers are the same as the previous mdw breadth score markers (with the text tag "trunc1" dropped) with difference as follows. For the previous markers, before deriving the MDW score, Net MFIs were truncated between 100 and 22000, and nonresponder Net MFI were set to 100. For the trunc1 markers, before deriving the MDW score, Net MFIs were truncated between 1 and 22000, and nonresponder Net MFI were set to 1.

3 Updates in the v10 SAP

This v10 SAP modifies the exploratory marker IgG V2p breadth score to combine over two additional antigens:

- IgG3 V2p breadth score, computed as the maximal diversity weighted linear combination of IgG3 V1V2 readouts to the gp70-1394C9G1 V1V2, gp70-BF1266 431a V1V2, gp70-Ce1086 B2 V1V2, C.1086C V1V2 Tags, and AE.A244 V1V2 Tags 293F antigens.

4 Updates in the v9 SAP

This v9 SAP adds a IgG3 V1V2 breadth score variable mdw_xassay_select_igg3v1v2 that is a maximal signal diversity weighted breadth score calculated using the 4 variables (antigens that were studied in the RV144 trial):

- IgG3 AE.A244 V1V2 Tags 293F
- IgG3 C.1086C V1V2 Tags
- IgG3 gp70-Ce1086 B2 V1V2
- IgG3 gp70-B.CaseAV1V2

The ADCC peak breadth score and the ADCC pAUC breadth score are each modified to not include antigen-specific readouts (among the three antigens studied) for which there is a negative antigen-specific response. For each antigen, a response is defined as positive if the peak baseline-subtracted percent loss Luciferase activity is greater than or equal to 10%, and otherwise the response is negative. Thus, each breadth score only includes positive antigen-specific responses. This process means the updated ADCC breadth variables can take the value 0. The variables are analyzed on the transformed scale transformed-ADCC breadth = 0 if the original ADCC breadth = 0 and transformed-ADCC breadth = log10(ADCC breadth) if the original ADCC breadth exceeds 1 (no original ADCC breadth values are between 0 and 1).

This SAP specifies that the ELISpot PTE Env primary marker is included in the correlates analyses, and the Multi-epitope functions primary marker is also included, because updated/corrected ELISpot data became available.

The definition of a positive response for the ELISpot PTE Env primary marker was updated (Section 20.2).

This SAP updates the "handling missing data" section, changing the first sentence of the second paragraph to: "First, we define the two-phase sampling indicator ϵ as taking value

of one if a participant has data available at Month 7 for the three primary immune markers ELISA VT-C data, ADCP gp140 C97ZA antigen, and IgG3 V1V2 breadth score."

Lastly, this SAP specifies the scope of the immune correlates analyses that will be included in the first immune correlates manuscript (Section 14.4).

5 Updates to the v8 SAP

The v8 SAP adds the following IgA markers measured at Month 7:

```
IgA Con 6 gp120/B
IgA 1394C9_G1.D11gp120.avi
IgA 1428_D11gp120.avi/293F
IgA 1012_11.TC21D11gp120.avi
IgA 1086C_D7gp120.avi/293F
IgA gp140 Mos1 fibritin
IgA gp140 C97ZA fibritin
IgA Con S gp140 CFI
IgA 1012_gp140C.avi/293F
IgA 1394C9_gp140C.avi/293F
IgA 1086C gp140C_avi
IgA BF1266_gp140C.avi/293F
IgA 9004S.gp140C.avi
IgA 00MSA 4076 gp140
IgA A1.con.env03 140 CF
IgA gp70-1012.11.TC21.3257 V1V2
IgA gp70-1394C9G1 V1V2
IgA gp70-BF1266_431a_V1V2
IgA gp70-001428.2.42 V1V2
IgA gp70-Ce1086_B2 V1V2
```

In addition, a maximal diversity weighted (MDW) IgA Env breadth score (variable name IgA-Env-breadth-score) is calculated based on all of the IgA gp120 and gp140 variables listed above. The IgA-Env-breadth score is computed by first computing MDW IgA-gp120 and MDW IgG-gp140 breadth scores based on all gp120 antigens and all gp140 antigens, respectively, and secondly calculating the IgA-Env-breadth score as: (MDW score for gp120 $\times$ tree-weight for MDW score for gp120) + (MDW score for gp140 $\times$ tree-weight for MDW score for gp140.

Lastly, this SAP adds two ADCC breadth score variables: one the average of the three variables ADCC CAP8 peak, ADCC CH58 peak, and ADCC WITO peak (ADCC peak breadth score); and the other the average of the three variables ADCC CAP8 pAUC, ADCC CH58 pAUC, and ADCC WITO pAUC (ADCC pAUC breadth score). The ADCC peak breadth score and the ADCC pAUC breadth score are each modified to not include antigen-specific readouts (among the three antigens studied) for which there is a negative antigen-specific response. For each antigen, a response is defined as positive if the peak baseline-subtracted percent loss Luciferase activity is greater than or equal to 10%, and otherwise the response is negative. Thus, each breadth score only includes positive antigen-specific responses.

Exploratory analyses are done assessing each of the above-listed individual Month 7 immune markers as correlates of risk, controlled VE CoPs, and mediators. In addition, a multivariable Cox model is fit including the IgA-Env-breadth-score variable, as described in Section 18.1.7.

6 Updates in this v7 SAP

This v7 SAP adds 1 exploratory marker:

1. ADCP gp70-1428 V1V2, measured by the Alter lab

The new exploratory marker is described in Section 16.1.

7 Updates to v6 SAP

This v6 SAP adds 7 exploratory markers:

1. IgG3 V2i breadth score, computed as the maximal diversity weighted linear combination of IgG3 V1V2 readouts to the gp70-1428, gp70-1012, and gp70-BCaseA antigens
2. IgG3 V2p breadth score, computed as the maximal diversity weighted linear combination of IgG3 V1V2 readouts to the gp70-1086, gp70-1266, and gp70-1394 antigens
3. Individual IgG3 gp120 variables for each of the 5 gp120 antigens included in the gp120 antigen breadth panel

The 7 exploratory markers are described in Section 16.1.

8 Updates to v5 SAP

This v5 SAP adds 12 exploratory markers, 6 each for CD4+ T cells and for CD8+ T cells. In the v4 SAP, there are 2 ICS exploratory markers: Percentage of CD4+ T cells expressing IFN-gamma and/or IL-2, and percentage of CD8+ T cells expressing IFN-gamma and/or IL-2, each for the Any-Env antigens. These Any-Env antigen markers are defined based on the 4 individual Env peptide pools JMos1gp120, JMos1gp41, JMos2Sgp120, JMos2Sgp41 as follows: Any MOS1 Env = sum(J Mos1 gp120, J Mos1 gp41), Any MOS2 Env = sum(J Mos2S gp120, J Mos2S gp41), and Any Env = max(Any MOS1 Env, Any MOS2 Env). The 6 new markers for each of CD4+ T cells and CD8+ T cells are defined for these 4 individual Env peptide pools and for the non-Env peptide pools JMos2Gag and JMos2RNAseInt.

9 Updates to v4 SAP

The v3 SAP included markers involving the ELISpot assay. It was discovered that this assay had technical issues with implication that the CSR will be populated with correlates results excluding markers that include ELISpot readouts (there are 2 such markers: ELISpot PTE Env and Expanded multi-epitope functions). In addition, the two ELISA markers have been updated with the latest ELISA PPD data received. This v4 SAP is identical to the v3 SAP except with (i) the 2 ELISpot markers removed, (ii) the ENSEMBLE superlearning results removed, and (iii) applied to the updated ELISA markers, as detailed below.

1. Sections 16.1 and 17.1.1 have removed the ELISpot PTE Env marker variable, as well as the single score variable that included the ELISpot PTE Env marker variable, Expanded multi-epitope functions. Thus two fewer primary markers are analyzed; four remain.
2. The multivariable correlate of risk modeling and the Westfall-Young multiplicity adjustment now includes the set of four primary variables excluding the ELISpot PTE Env and Expanded multi-epitope functions primary variables (Section 18.1.5).
3. The Superlearner objective is canceled for this SAP, because this multivariable analysis will be conducted after the ELISpot PTE Env marker and the new Expanded multi-epitope functions variables are included.

10 Updates to v3 SAP

For the correlates analyses based on the v2 SAP, the primary marker ELISpot PTE Env was not yet available, and as such the v2 correlates results were preliminary with no intention to disseminate the results. In March of 2022 the ELISpot PTE Env marker data became available, such that results from this v3 correlates SAP constitute a complete correlates analysis that may be considered for dissemination.

1. The ELISpot PTE Env marker variable has been added as a primary variable (Section 16.1). Section 17.1.1 has been updated to reflect 6 primary markers. Section 20.2 describes the details of the ELISpot PTE Env variable.
2. Two ICS markers have been added as non-primary variables: Percentage of CD4+ T cells expressing IFN-gamma and/or IL-2, and percentage of CD8+ T cells expressing IFN-gamma and/or IL-2, each for the ANY-ENV antigen. The new variables are listed in Section 16.1.
3. The multivariable correlate of risk modeling and the Westfall-Young multiplicity adjustment now includes the set of six primary variables (Section 18.1.5).

4. A second ADCP marker variables has been added (in the non-primary tier): ADCP Mos 1 (Section 16.1).

5. Six new ADCC marker variables (in the non-primary tier) have been added (Section 16.1).

6. A BAMA IgG total (IgGt) V1V2 breadth score variable has been added (Section 16.1).

7. A new overall combined maximum signal diversity weighted score variable has been added, which is calculated the same as the previous overall combined score, except that it also includes ELISpot PTE Env, IgGt V1V2 breadth, and the three ADCC pAUC markers into the score (Section 16.1). This new marker is placed in the primary tier, supplanting the previous overall combined score, which is retained as a non-primary tier marker.

8. 17 individual V1V2 antigen non-primary/exploratory variables were added for correlates analyses. These variables are included in the set of univariable correlates analyses. The 17 variables are listed in Section 16.1.

9. For univariable marker analyses, all of the added marker variables are analyzed in the same way as the marker variables that were analyzed in the v2 SAP; both the v2 and v3 SAPs state that each marker variable is evaluated.

10. In Section 16.1, additional updates include a revised summary of the rationale for the markers, and lower and upper truncation limits for the new markers have been added.

11. In the v2 SAP, it was noted that the multivariable correlate of risk estimated optimal surrogate Objective 7 could not be conducted until additional markers were measured. For this v3 SAP, these variables are now available. Accordingly, the marker variable sets for this Superlearner objective have been updated.

12. In Section 18.1.2, the G-computation formula (2) has been changed to remove inverse probability weighting, because G-computation marginalizes over the phase 1 baseline covariates that are measured in everyone, such that this change may provide more efficient estimation. The previous approach that used inverse probability weighting is correct; it may be less efficient. Similarly, the G-computation formula (3) was changed in the same way.

13. The van der Laan et al. (2021) citation was updated (this paper is in press at Biometrics as of March 27, 2022).

14. Changed the title of the SAP to note "Month 7 Marker Analyses."

11 Updates to v2 SAP

1. In the previously-included section on Superlearner multivariable CoR analysis, added the following text: “In addition, all Month 7 individual markers that are constituents for defining one or more of the 12 markers are included; for example the antigen-specific breadth score variables aggregate over readouts to a set of antigens. Therefore, for example, the variable set “All BAMA IgG3 gp140 markers” in the table includes all individual antigen IgG gp140 markers as well as including the IgG3 gp140 breadth score marker.

2. Fixed a typo in a table in the previously-included section on Superlearner multivariable CoR analysis in to clarify that HIV-1 infection endpoint diagnosed through the Month 24 visit are studied.

3. The right-censoring time used in time-to-event analyses is now defined (Section 17).

4. Updated the IgG3 Env breadth score to only include variables 6, 7, dropping variables 4 and 5. Variables 4 and 5 (gp140 vaccine antigens) are excluded because the IgG3 Env breadth score already includes the gp140 vaccine antigens.

12 Updates to v1 SAP

1. Updated the V1V2 IgG3 breadth score to not include the vaccine strain antigen

2. Updated the Env IgG3 breadth score to include the 2 gp140 vaccine strain antigens

3. Updated the IgG3 Env breadth score to include variables 4, 5, 6, 7

4. Updated the Overall combined maximal signal diversity weighted score variable to only include variables 1, 3, 8, 11. Variables 4. and 5. (gp140 vaccine antigens, variable umbering from v1 SAP) are excluded because the IgG3 Env breadth score already includes the gp140 vaccine antigens.

5. Updated the BAMA IgG3 multi-epitope breadth score to include V1V2.

6. The set of vaccine arm cases for inclusion in the analysis was changed in Section 15.2. In particular, “PPcases7to36” was changed to “PPcases7to24” in the sentence “immune markers are measured at Month 7 (and typically also Month 0) in all vaccine recipient cases in the PPcases7to24 cohort” and in one other sentence in that section. The current text is now consistent with the already-implemented HVTN 705 / VAC89220HPX2008 Case Control Correlate Study Sampling Plan.

7. A nonparametric sensitivity analysis was added to the approach for assessing for the presence of a Controlled vaccine efficacy correlate of protection (Section 17.2).

13 Correlates Study Objectives from the Protocol

The following study objectives, copied from the protocol, are considered to be "correlates" study objectives, and the purpose of this statistical analysis plan (SAP) is to describe the statistical methods that will be used to answer these study objectives, as well as closely related correlates objectives. The endpoints listed in the protocol corresponding to these objectives are also recapitulated here.

Secondary objective 6: To evaluate immunogenicity and immune response biomarkers among vaccine recipients after the third vaccination as correlates of risk of subsequent HIV acquisition and correlates of vaccine efficacy, if deemed applicable.

Secondary endpoint 6: Immune responses from assays based on the HVTN Laboratory Assay Algorithm (available at https://atlas.scharp.org/) and/or more assays down-selected from a larger pool of pilot studies, in HIV-1-infected vaccine cases and HIV-1-uninfected vaccine controls.

Secondary objective 8: If significant positive evidence of vaccine efficacy from month 7 through 24 months is seen, to assess if and how vaccine efficacy depends on genotypic characteristics of HIV such as signature mutations

Secondary endpoint 8: HIV-1 infection diagnosed after month 7 through Month 24 and genotypic characteristics of viral sequences from HIV-1-infected participants at HIV-1 diagnosis, such as signature site mutations.

Secondary objective 9: To evaluate and compare genomic sequences of viral isolates from HIV-1-infected vaccine and placebo recipients, and use sieve analysis methods to assess whether VE differs by genotypic or phenotypic characteristics of exposing HIVs and whether there is evidence of vaccine-induced immune pressure on the viral sequences.

Secondary endpoint 9: Viral sequences from HIV-1-infected participants at the earliest available post-infection timepoint and possibly subsequent visits.

Exploratory objective 3: To evaluate early and innate immune responses (eg, whole blood transcriptomics, serum cytokines) one day after the third vaccination (ie, the first protein boost) as correlates of risk of subsequent HIV acquisition.

Exploratory objective 6: To assess use of biomedical interventions and biological and behavioral factors in the study cohort and how they modify vaccine efficacy. In addition, the ADCC peak breadth score and the ADCC pAUC breadth score are each modified to not include antigen-specific readouts (among the three antigens studied) for which there is a negative antigen-specific response. For each antigen, a response is defined as positive if the peak baseline-subtracted percent loss Luciferase activity is greater than or equal to 10%, and otherwise the response is negative. Thus, each breadth score only includes positive antigen-specific responses. This process means the updated ADCC breadth variables can take value 0. The variables are analyzed on the transformed scale transformed-ADCC breadth = 0 if

the original ADCC breadth = 0 and transformed-ADCC breadth = log10(ADCC breadth) if the original ADCC breadth exceeds 1 (no original ADCC breadth values are between 0 and 1). In addition, the ADCC peak breadth score and the ADCC pAUC breadth score are each modified to not include antigen-specific readouts (among the three antigens studied) for which there is a negative antigen-specific response. For each antigen, a response is defined as positive if the peak baseline-subtracted percent loss Luciferase activity is greater than or equal to 10%, and otherwise the response is negative. Thus, each breadth score only includes positive antigen-specific responses. This process means the updated ADCC breadth variables can take valuethe 0. The variables are analyzed on the transformed scale transformed-ADCC breadth = 0 if the original ADCC breadth = 0 and transformed-ADCC breadth = log10(ADCC breadth) if the original ADCC breadth exceeds 1 (no original ADCC breadth values are between 0 and 1). **Exploratory objective 7:** To evaluate the role of host genetic factors in the immune response to the vaccine regimen and in vaccine effects on study endpoints.

14 Re-structuring the Correlates Objectives

This section defines a fuller set of correlates objectives that constitute sub-objectives of those specified above. In this structuring secondary objective 6 is a Month 7 Adaptive CoR/CoP objective, secondary objectives 8 and 9 are collectively the Amino acid (AA) sequence sieve analysis objective, and exploratory objective 3 is the Month 6.1 Innate CoR objective. Exploratory objective 6 refers either to assessment of baseline or post-vaccination potential vaccine efficacy modifiers.

14.1 Host Variables (Correlates Objectives)

1. Baseline CoR: To assess baseline markers as correlates of HIV-1 acquisition
2. Baseline coVE: To assess baseline markers as correlates of VE (= coVE)
3. Post-vaccination CoR: To assess post-vaccination markers as correlates of HIV-1 acquisition
4. Post-vaccination coVE: To assess post-vaccination markers if assigned to the vaccine group as correlates of VE (principal stratification)
5. Post-vaccination contVE: To assess post-vaccination markers as controlled vaccine efficacy (contVE) CoPs (Gilbert et al., 2022).
6. Post-vaccination mediation: To assess post-vaccination markers as mediators of VE
7. Post-vaccination multivariable CoR (individual-level signatures): To build conditional risk models based on baseline demographics and potential HIV-1 exposure variables,

baseline marker variables, and post-vaccination marker variables, as correlates of HIV-1 acquisition

8. Post-vaccination CoR (outcome-proximal): To assess current values (or histories up to a current time point) of post-vaccination markers as correlates of instantaneous HIV-1 acquisition

9. Post-vaccination mediation (outcome-proximal): To assess current values (or histories up to a current time point) of post-vaccination markers as mediators of instantaneous VE

Objectives 1–7 above are "approximate peak time point" correlates objectives, which assess associations (or effects) of markers measured post-vaccination with subsequent risk of HIV-1 acquisition over a specified follow-up period, and the post-vaccination markers focus on innate (Month 6.1) and adaptive (Month 7, Month 13) immune markers. Objectives 8–9 are "outcome-proximal" correlates objectives, which assess associations (or effects) of current values of markers with the instantaneous risk of HIV-1 acquisition. These analyses would require sampling all available longitudinal time points for HIV-1 infection cases and controls.

14.2 HIV-1 Pathogen Variables (Sieve Analysis Objectives)

10. AA sequence sieve analysis: To assess how VE depends on AA sequence features of exposing HIV-1 viruses

11. Phenotypic sieve analysis: To assess how VE depends on measured immunological features of exposing HIV-1 viruses

14.3 Host and HIV-1 Pathogen Variables (Integrated Correlates and Sieve Analysis Objectives)

12. Baseline CoR of Type-Specific HIV-1 Acquisition: To assess baseline markers as correlates of mark-specific HIV-1 acquisition and whether the correlation differs by mark.

13. Post-vaccination CoR of Type-Specific HIV-1 Acquisition: To assess post-vaccination markers if assigned to the vaccine group as correlates of mark-specific HIV-1 acquisition and whether the correlation differs by mark.

These analyses would only be considered if there is evidence for beneficial positive overall vaccine efficacy from the efficacy analyses.

14.4 Scope of this Correlates SAP

This SAP focuses on the assessment of peak immune time point (Month 7 visit) CoR and CoP objectives 3, 5, 6, for HIV-1 acquisition diagnosis through to the Month 24 visit.

The three included objectives can be stated as follows.

3. Post-vaccination CoR: To assess each individual Month 7 immune marker (primary and exploratory) as a CoR of HIV-1 acquisition diagnosed by the Month 36 visit, adjusting for baseline factors, and also to assess the Month 7 primary immune markers together as a multivariable CoR of HIV-1 acquisition diagnosed by the Month 36 visit.

5. Post-vaccination contVE: To assess each individual Month 7 immune marker (primary and exploratory) as a controlled VE CoP (contVE) against HIV-1 acquisition diagnosed by the Month 36 visit.

6. Post-vaccination mediation: To assess each individual Month 7 immune marker as a mediator of vaccine efficacy against HIV-1 acquisition diagnosed by the Month 36 visit.

There are a total of 90 primary and exploratory immune markers. The first immune correlates manuscript restricts to the six primary markers, all of the variables that are constituents of the sixth primary marker (multi-epitope functions, totaling 3 additional markers that includes ADCC and ICS markers), the IgG3 V1V2 individual-antigen variables, and the 4 trunc1 MDW IgG3 V1V2 breadth score markers. This adds up to 25 immune marker variables (6 primary markers, 7 additional individual markers integrated into the multi-epitope functions primary marker, 8 individual-antigen IgG3 V1V2 markers, 4 trunc1 MDW IgG3 V1V2 breadth score markers). In this approach, the multivariable CoR statistical learning (superlearning) objective that analyzes all 90 markers will be reported in a second immune correlates manuscript. Listed out, the 25 markers included in the first manuscript are:

1. The 7 primary markers: ELISA VT-C, ADCP C97ZA, BAMA IgG3 V1V2 breadth score, BAMA IgG3 Env breadth, CD4+ T cells Any-Env IFNgamma/IL-2, CD8+ T cells Any-Env IFNgamma/IL-2, Multi-epitope functions

2. The 5 additional individual markers that are included in the multi-epitope functions primary marker: ADCP Mos1 antigen, IgG total V1V2 breadth, ADCC CAP8 pAUC, ADCC CH58 pAUC, ADCC WITO pAUC

3. The 8 individual-antigen IgG3 V1V2 variables: IgG3 AE.A244 V1V2 Tags_293F, IgG3 C.1086C_V1_V2 Tags, IgG3 gp70-001428.2.42 V1V2, IgG3 gp70-1012.11.TC21.3257 V1V2, IgG3 gp70-1394C9G1 V1V2, IgG3 gp70-BF1266_431a_V1V2, IgG3 gp70-Ce1086_B2 V1V2, IgG3 gp70_B.CaseA_V1_V2

4. The 4 trunc1 markers IgG340mdw_V1V2_trunc1, mdw_xassay_select_igg3v1v2_trunc1, IgG340mdw_V2i_trunc1, IgG340mdw_V2p_trunc1

15 Cohorts Relevant for the Assessment of Correlates

15.1 Cohort Definitions

The following study cohorts or analysis sets are used for addressing the correlates study objectives. The terminology for the first 9 cohorts is also used in the protocol and in the safety and efficacy statistical analysis plan.

1. **Modified Intent-to-Treat (MITT) cohort:** participants in the Full Analysis Set (FAS) who are HIV-1 uninfected on the date of first vaccination.
2. **Per-Protocol (PP) cohort:** participants in the FAS who are HIV-1 uninfected 4 weeks after the 3rd vaccination visit, who received all planned vaccinations at the first 3 vaccination visits within the respective visit windows and have no other major protocol deviations that were judged to possibly impact the efficacy of the vaccine.
3. **Full Immunization Set (FIS):** participants in the FAS who are HIV-1 uninfected 4 weeks after the 4th vaccination visit and who receive all planned vaccinations within the respective visit windows.
4. **At risk Immunogenicity Cohort (IC-at risk):** participants in the FAS who are selected for measurement of immune response endpoints at the primary immunogenicity timepoints and who are HIV-1 uninfected 4 weeks after the 3rd vaccination visit, who have no other major protocol deviations that were judged to possibly impact the efficacy of the vaccine.
5. **Per Protocol Immunogenicity Cohort (IC-PP):** Participants in the IC-at risk who received all planned vaccinations at the first 3 vaccination visits within the respective visit windows.
6. **MITT Infected by 24 Months Cohort (MITTcases0to24):** Participants in the MITT cohort who are diagnosed with HIV-1 infection during the follow-up period after enrollment through the Month 24 visit.
7. **MITT Infected by 36 Months Cohort (MITTcases0to36):** Participants in the MITT cohort who are diagnosed with HIV-1 infection during the follow-up period after enrollment through the Month 36 visit.
8. **Per-Protocol Infected by 24 Months Cohort (PPcases7to24):** Participants in the IC-PP cohort who are diagnosed with HIV-1 infection during the follow-up period on or after the Month 7 visit through the Month 24 visit.
9. **Per-Protocol Infected by 36 Months Cohort (PPcases7to36):** Participants in the IC-PP cohort who are diagnosed with HIV-1 infection during the follow-up period on or after the Month 7 visit through the Month 36 visit.

10. **Full Immunization Set Infected by 24 Months Cohort (FIScases13to24):** Participants in the FIS cohort who are diagnosed with HIV-1 infection during the follow-up period on or after the Month 7 visit through the Month 24 visit.

11. **Full Immunization Set Infected by 36 Months Cohort (FIScases13to36):** Participants in the FIS cohort who are diagnosed with HIV-1 infection during the follow-up period on or after the Month 7 visit through the Month 36 visit.

12. **Month 24 Vaccine Group Controls (VxControls24):** Participants in the IC-PP cohort who reach the Month 24 visit with a negative HIV-1 test result and have sample availability at the baseline, Month 6.1, Month 7, and Month 13 time points.

13. **Month 27 Vaccine Group Controls (VxControls27):** Participants in the IC-PP cohort who reach the Month 27 visit with a negative HIV-1 test result and have sample availability at the baseline, Month 6.1, Month 7, and Month 13 time points.

14. **Selected Month 27 Vaccine Group Controls (SelectedVxControls27):** The subset of the VxControls27 cohort that is randomly sampled for the case-control study (for measurement of immune response biomarkers).

15. **Month 36 Vaccine Group Controls (VxControls36):** Participants in the IC-PP cohort who reach the Month 36 visit with a negative HIV-1 test result and have sample availability at the baseline, Month 6.1, Month 7, and Month 13 time points.

16. **Month 30 Vaccine Group Controls (VxControls30):** Participants in the IC-PP cohort who reach the Month 30 visit with a negative HIV-1 test result and have sample availability at the baseline, Month 6.1, Month 7, and Month 13 time points.

15.2 Two-phase case-control sampling design

A two-phase case-control sampling design is used, in which the immune markers are measured at Month 7 (and typically also Month 0) in all vaccine recipient cases in the PPcases7to24 cohort and in a stratified random sample of vaccine recipient non-cases in the VxControls27 cohort. Within each demographic stratum defined by randomization arm cross-classified with the six substrata defined by (BMI$<$25, 25$\leq$BMI$<$30, BMI$\geq$30)$\times$(RSA, Non-RSA), a without-replacement sample of non-cases is randomly sampled, with sample size equal to five times the number of vaccine recipient cases in the demographic stratum. In addition, 1:1 case:control sampling is employed in these strata in the placebo arm. Data from this small number of placebo recipients is included for verification of appropriate specificity of the immunoassays to not detect HIV-1 specific positive responses in placebo recipients. Further details of the case-control sampling plan can be found in the HVTN 705 / VAC89220HPX2008 Case Control Correlate Study Sampling Plan.

The current SAP considers analyses that make inferences for the population that is represented by the IC-PP cohort. Participants in the PPcases7to24 cohort and participants in

the IC-PP and SelectedVxControls27 cohort are included in the data analyses. Controls are selected among those in the IC-PP cohort who reached their Month 27 visit with an HIV negative test, rather than their Month 24 visit, to avoid the possibility of inadvertently including a Month 7-24 case in the set of controls used for analysis. Indeed, in the interim data used to generate the case-control cohort, a Month 24 HIV-negative control with no follow-up beyond the Month 24 visit (which was well possible at the time of sampling) could become seropositive at M27, and retrospective testing could result in an RNA-positive sample at Month 24. Restricting the sampled controls to those who were HIV negative at Month 27 ensures that participants sampled as controls will retain a Month 24 negative status with high probability. Though this sampling plan avoids the undesirable possibility of inadvertently including a Month 7-24 case in the set of controls used for analysis, it does entail that IC-PP Month 7-24 controls who enroll late in the trial may not be eligible for sampling because they have not yet reached their Month 27 visit, whereas IC-PP Month 7-24 cases that enrolled around the same time will be eligible. This would only be problematic if there were a temporal trend in incidence or vaccine efficacy that is controlled for neither by the stratified sampling scheme used to select controls that match cases by BMI $\times$ RSA/Non-RSA categories nor the adjustment for the baseline risk score that is employed in the conducted correlates analyses. Such a temporal trend has been deemed unlikely, and it has been judged to be more important to ensure that sampled controls are, in fact, Month 24 controls with high probability.

Additional cases, such as post-M24 cases, and corresponding matched controls will be included in future analyses if their samples become available.

16 Baseline and Month 7 Immune Marker Variables for CoR and CoP Analyses

We summarize the baseline and Month 7 immune marker variables that are needed for implementation of this SAP. Analysis of the pilot study data is used to finalize the list of baseline and Month 7 immune markers that are included in the correlates analyses. In addition, based on the pilot study data the Month 7 markers are divided into a primary tier and an exploratory tier. The reporting of results is segregated by tier, with formal hypothesis testing only applied to the primary tier markers; this hypothesis testing for Objective 3 tests for a CoR and for Objective 5 tests for a contVE CoP. Multiple hypothesis testing adjustment is applied to the set of primary tier markers.

16.1 Month 7 Markers

The following Month 7 types of immune markers are assessed as CoRs and CoPs, usually as quantitative variables and in some analyses as ordered trinary variables or binary variables,

all of which do not subtract Month 0 (baseline) values. The immune markers in the primary tier are noted; all other markers as studied as correlates in the exploratory tier.

1. [**Primary**]: ELISA VT-C
2. ELISA VT-M
3. [**Descriptive only**]: ELISpot PTE Env
4. [**Primary**]: ADCP C97ZA antigen
5. ADCP Mos1 antigen
6. BAMA IgG3 vaccine-matched gp140 C97ZA fibritin
7. BAMA IgG3 vaccine-matched gp140 Mosaic fibritin
8. BAMA IgG3 gp120 breadth score based on all antigens evaluated other than vaccine strain antigens, computed using maximal signal diversity weighting [He and Fong (2019) method]
9. BAMA IgG3 gp140 breadth score based on all antigens evaluated including vaccine strain antigens, computed using maximal signal diversity weighting
10. [**Primary**]: BAMA IgG3 V1V2 breadth score based on all antigens with evaluated other than vaccine strain antigens, computed using maximal signal diversity weighting
11. BAMA IgG3 V1V2 breadth score restricting to antigens in common with RV144 antigens, computed using maximal signal diversity weighting
12. BAMA IgG3 gp41
13. BAMA multi-epitope breadth score: Maximal signal diversity weighted score of the 4 breadth scores: BAMA IgG3 gp120, gp140, gp41, V1V2
14. [**Primary**]: BAMA IgG3 Env breadth: Maximal signal diversity weighted score of the BAMA IgG3 gp120 and gp140 breadth variables 8, 9 above
15. BAMA IgG total V1V2 breadth score based on all antigens with evaluated other than vaccine strain antigens, computed using maximal signal diversity weighting
16. [**Primary**]: Maximal signal diversity weighted score of variables 1, 4, 10, 14 above (ELISA VT-C, ADCP C97ZA , BAMA IgG3 V1V2 breadth score, BAMA IgG3 Env breadth)
17. ADCC CAP8 peak
18. ADCC CH58 peak

19. ADCC WITO peak
20. ADCC CAP8 pAUC
21. ADCC CH58 pAUC
22. ADCC WITO pAUC
23. [**Primary**]**:** CD4+ T cells Any-Env IFNgamma/IL-2
24. [**Primary**]**:** CD8+ T cells Any-Env IFNgamma/IL-2
25. [**Primary**]**:** (Multi-epitope functions) Overall combined maximal signal diversity weighted score of variables 1, 4, 5, 10, 14, 15, 20, 21, 22, 23, 24 above (ELISA VT-C, ADCP C97ZA, ADCP Mos1 antigen, BAMA IgG3 V1V2 breadth score, BAMA IgG3 Env breadth, IgG total V1V2 breadth, ADCC CAP8 pAUC, ADCC CH58 pAUC, ADCC WITO pAUC, CD4+ T cells Any-Env IFNgamma/IL-2, CD8+ T cells Any-Env IFNgamma/IL-2)
26. IgG3 AE.A244 V1V2 Tags_293F
27. IgG3 C.1086C_V1_V2 Tags
28. IgG3 gp70-001428.2.42 V1V2
29. IgG3 gp70-1012.11.TC21.3257 V1V2
30. IgG3 gp70-1394C9G1 V1V2
31. IgG3 gp70-BF1266_431a_V1V2
32. IgG3 gp70-Ce1086_B2 V1V2
33. IgG3 gp70_B.CaseA_V1_V2
34. IgG AE.A244 V1V2 Tags_293F
35. IgG C.1086C_V1_V2 Tags
36. IgG gp70-001428.2.42 V1V2
37. IgG gp70-1012.11.TC21.3257 V1V2
38. IgG gp70-1394C9G1 V1V2
39. IgG gp70-9004SS.A3.4 V1V2
40. IgG gp70-BF1266.431a V1V2
41. IgG gp70-Ce1086.B2 V1V2

42. IgG gp70.B.CaseA V1_V2
43. CD4+ T cells JMos1gp120 IFNgamma/IL-2
44. CD4+ T cells JMos1gp41 IFNgamma/IL-2
45. CD4+ T cells JMos2Sgp120 IFNgamma/IL-2
46. CD4+ T cells JMos2Sgp41 IFNgamma/IL-2
47. CD4+ T cells JMos2Gag IFNgamma/IL-2
48. CD4+ T cells JMos2RNAseInt IFNgamma/IL-2
49. CD8+ T cells JMos1gp120 IFNgamma/IL-2
50. CD8+ T cells JMos1gp41 IFNgamma/IL-2
51. CD8+ T cells JMos2Sgp120 IFNgamma/IL-2
52. CD8+ T cells JMos2Sgp41 IFNgamma/IL-2
53. CD8+ T cells JMos2Gag IFNgamma/IL-2
54. CD8+ T cells JMos2RNAseInt IFNgamma/IL-2
55. IgG3 v2i breadth score
56. IgG3 v2p breadth score
57. IgG3 V1V2 breadth score trunc1
58. IgG3 V1V2 breadth score selected trunc1
59. IgG3 V2i breadth score trunc1
60. IgG3 V2p breadth score trunc1
61. IgG3 1012_11.TC21D11gp120.avi
62. IgG3 1086C_D7gp120.avi/293F
63. IgG3 1394C9_G1.D11gp120.avi
64. IgG3 1428_D11gp120.avi/293F
65. IgG3 Con 6 gp120/B
66. IgA Con 6 gp120/B
67. IgA 1394C9_G1.D11gp120.avi

68. IgA 1428_D11gp120.avi/293F
69. IgA 1012_11.TC21D11gp120.avi
70. IgA 1086C_D7gp120.avi/293F
71. IgA gp140 Mos1 fibritin
72. IgA gp140 C97ZA fibritin
73. IgA Con S gp140 CFI
74. IgA 1012_gp140C.avi/293F
75. IgA 1394C9_gp140C.avi/293F
76. IgA 1086C gp140C_avi
77. IgA BF1266_gp140C.avi/293F
78. IgA 9004S.gp140C.avi
79. IgA 00MSA 4076 gp140
80. IgA A1.con.env03 140 CF
81. IgA gp70-1012.11.TC21.3257 V1V2
82. IgA gp70-1394C9G1 V1V2
83. IgA gp70-BF1266_431a_V1V2
84. IgA gp70-001428.2.42 V1V2
85. IgA gp70-Ce1086_B2 V1V2
86. IgA-Env-breadth-score
87. ADCC peak breadth score
88. ADCC pAUC breadth score
89. ADCP gp70-1428 V1V2
90. IgG3 CaseA2 Alter

The IgG3 v2i breadth score is computed as the maximal diversity weighted linear combination of IgG3 V1V2 readouts to the gp70-1428, gp70-1012, and gp70-BCaseA antigens. It is computed the same way as the primary variable IgG breadth score, except restricting to 3 antigens rather than including all 8. The IgG3 v2p breadth score is computed in the same way, restricting to the 5 antigens gp70-1394C9G1 V1V2, gp70-BF1266 431a V1V2, gp70-Ce1086 B2 V1V2, C.1086C V1V2 Tags, and AE.A244 V1V2 Tags 293F antigens. The last 5 exploratory variables are IgG3 readouts to each of the five individual gp120 antigens included in the gp120 antigen breadth panel.

The ADCC peak breadth score is defined as the average of the three variables ADCC CAP8 peak, ADCC CH58 peak, and ADCC WITO peak (ADCC peak breadth score). The ADCC pAUC breadth score is defined as the average of the three variables ADCC CAP8 pAUC, ADCC CH58 pAUC, and ADCC WITO pAUC (ADCC peak breadth score). These averages do not include antigen-specific readouts (among the three antigens studied) for which there is a negative antigen-specific response. For each antigen, a response is defined as positive if the peak baseline-subtracted percent loss Luciferase activity is greater than or equal to 10%, and otherwise the response is negative. Therefore, each breadth score only includes positive antigen-specific responses. The two analyzed ADCC breadth score variables are each transformed as follows: transformed-ADCC breadth = 0 if the original ADCC breadth = 0 and transformed-ADCC breadth = log10(ADCC breadth) if the original ADCC breadth exceeds 1. This process works because no original ADCC breadth values fall in (0,1).

16.2 Rationale for the 7 primary immune markers

We summarize a rationale for designation of the 7 primary markers. ELISA VT-C and ADCP C97ZA antigen were correlates of protection for the Imbokodo vaccine regimen in the non-human primate model, and ADCP C97ZA is a functional marker. ELISA VT-C is selected over ELISA VT-M because of the stronger antibody response to VT-C and the stronger correlate of protection in the non-human primate model. BAMA IgG3 V1V2 breadth was one of the strongest inverse correlate of risk in the RV144 vaccine efficacy trial (Yates et al., 2014) and was an inverse correlate of risk for the Imbokodo vaccine regimen in the non-human primate model. BAMA IgG3 Env breadth was one of the strongest inverse correlates of risk in the HVTN 505 vaccine efficacy trial (Neidich et al., 2019).

ELISpot PTE Env was an inverse correlate of risk for the Imbokodo vaccine regimen in the non-human primate model, and on that basis was planned for inclusion as a primary marker. However, there was evidence of sample degradation based on the lower responses observed for the the ELISpot PTE Env biomarker performed on the case-control samples compared to on the pilot samples ($p < 0.001$), an observation that was not seen for the other assays. This observation is partially, but incompletely, explained by the change in the source of peptides and it also appears that there is a sample degradation effect that is quite consistent between the Beth Israel Deaconess Medical Center lab that generated the ELISpot data and the sub-pool mapping experiments performed at the Fred Hutchinson Cancer Center. There is

no full explanation for the approximately 3-fold reduction in response from the pilot samples to the case-control samples. This reduction implies it is likely that the ELISpot case-control data does not fully and quantitatively accurately reflect the immune response to vaccination, such that is not possible to properly test the NHP-based correlate hypothesis in Imbokodo with the ELISpot assay. Graphical displays of the ELISpot data, for both the pilot set and the case-control set, are included in the results reports, although the ELISpot data are not used in the correlates of risk or correlates of protection analyses.

Given the importance of including both T cell and antibody markers in the primary tier of immune markers studied as immune correlates, the CD4+ T cells Any-Env IFNgamma/IL-2 and CD8+ T cells Any-Env IFNgamma/IL-2 markers were re-classified as primary markers, for which there was no evidence of sample degradation. The ICS assay is more informative than the ELISpot assay given it distinguishes CD4+ vs. CD8+ T cell responses, and the ICS assay has been used as a primary marker in several past HIV vaccine efficacy trials (e.g., (Haynes et al., 2012; Li et al., 2013; Janes et al., 2017; Fong et al., 2018)).

In addition, the pilot study supports that there is limited correlation among the primary markers.

The following describes LLOQs and ULOQs of the immune marker variables, and indicates whether values are truncated before use in correlates analyses.

Immune Marker	Lower truncation	Upper truncation	Truncation comments
1. ELISA VT-C	LLOQ=80	ULOQ=1884160	<LLOQ set to LLOQ/2. >ULOQ set to ULOQ.
2. ELISA VT-M	LLOQ=100	ULOQ=1869824	<LLOQ set to LLOQ/2. >ULOQ set to ULOQ.
3. ELISpot PTE Env	1	None	
4. ADCP C97ZA	LLOQ=2.87	ULOQ=16.11	No truncation applied by request of lab
5. ADCP Mos 1	LLOQ=2.87	ULOQ=16.11	No truncation applied by request of lab
6. IgG3 Vx-matched gp140 C97ZA	1	22000	<1 set to 1 (1 = convenient lower value) >22000 set to 2200 (22000 = top of linear range)
7. IgG3 Vx-matched gp140 Mos1	1	22000	<1 set to 1 (1 = convenient lower value) >22000 set to 2200 (22000 = top of linear range)
8. IgG3 gp120 breadth score	NA	NA	Before deriving MDW score,

			Net MFIs truncated between 100 and 22000, and nonresponder Net MFI set to 100. MDW score not truncated.
9. IgG3 gp140 breadth score	NA	NA	Same comment as MDW gp120
10. IgG3 V1V2 breadth score	NA	NA	Same comment as MDW gp120
11. IgG3 V1V2 breadth score selected	NA	NA	Same comment as MDW gp120
12. IgG3 gp41	1	22000	<1 set to 1 (1 = convenient lower value) >22000 set to 2200 (22000 = top of linear range)
13. IgG3 multi-epitope breadth	NA	NA	Same comment as MDW gp120
15. IgG3 Env breadth	NA	NA	Same comment as MDW gp120
16. IgGt V1V2 breadth score	NA	NA	
17. ADCC CAP8 peak	0.01	NA	Set < 0.01 to 0.01
18. ADCC CH58 peak	0.01	NA	Set < 0.01 to 0.01
19. ADCC WITO peak	0.01	NA	Set < 0.01 to 0.01
20. ADCC CAP8 pAUC	0.01	NA	Set < 0.01 to 0.01
21. ADCC CH58 pAUC	0.01	NA	Set < 0.01 to 0.01
22. ADCC WITO pAUC	0.01	NA	Set < 0.01 to 0.01
23. CD4+ Any-Env	0.01	NA	Set % cells < 0.01 to 0.01
24. CD8+ Any-Env	0.01	NA	Set % cells < 0.01 to 0.01
25. Overall combined score	NA	NA	
26. IgG3 AE.A244 V1V2 Tags_293F	1	22000	<1 set to 1 (1 = convenient lower value) >22000 set to 2200 (22000 = top of linear range)
27. IgG3 C.1086C_V1_V2 Tags	1	22000	<1 set to 1 (1 = convenient lower value) >22000 set to 2200 (22000 = top of linear range)
28. IgG3 gp70-001428.2.42 V1V2	1	22000	<1 set to 1 (1 = convenient lower value) >22000 set to 2200 (22000 = top of linear range)
29. IgG3 gp70-1012.11.TC21.3257 V1V2	1	22000	<1 set to 1 (1 = convenient lower value) >22000 set to 2200 (22000 = top of linear range)
30. IgG3 gp70-1394C9G1 V1V2	1	22000	<1 set to 1 (1 = convenient lower value) >22000 set to 2200 (22000 = top of linear range)
31. IgG3 gp70-BF1266_431a_V1V2	1	22000	<1 set to 1 (1 =

			convenient lower value) >22000 set to 2200 (22000 = top of linear range)
32. IgG3 gp70-Ce1086_B2 V1V2	1	22000	<1 set to 1 (1 = convenient lower value) >22000 set to 2200 (22000 = top of linear range)
33. IgG3 gp70_B.CaseA_V1_V2	1	22000	<1 set to 1 (1 = convenient lower value) >22000 set to 2200 (22000 = top of linear range)
34. IgG AE.A244 V1V2 Tags_293F	1	22000	<1 set to 1 (1 = convenient lower value) >22000 set to 2200 (22000 = top of linear range)
35. IgG C.1086C_V1_V2 Tags	1	22000	<1 set to 1 (1 = convenient lower value) >22000 set to 2200 (22000 = top of linear range)
36. IgG gp70-001428.2.42 V1V2	1	22000	<1 set to 1 (1 = convenient lower value) >22000 set to 2200 (22000 = top of linear range)
37. IgG gp70-1012.11.TC21.3257 V1V2	1	22000	<1 set to 1 (1 = convenient lower value) >22000 set to 2200 (22000 = top of linear range)
38. IgG gp70-1394C9G1 V1V2	1	22000	<1 set to 1 (1 = convenient lower value) >22000 set to 2200 (22000 = top of linear range)
39. IgG gp70-9004SS.A3.4 V1V2	1	22000	<1 set to 1 (1 = convenient lower value) >22000 set to 2200 (22000 = top of linear range)
40. IgG gp70-BF1266.431a V1V2	1	22000	<1 set to 1 (1 = convenient lower value) >22000 set to 2200 (22000 = top of linear range)
41. IgG gp70-Ce1086.B2 V1V2	1	22000	<1 set to 1 (1 = convenient lower value) >22000 set to 2200 (22000 = top of linear range)

42. IgG gp70.B.CaseA V1_V2	1	22000	<1 set to 1 (1 = convenient lower value) >22000 set to 2200 (22000 = top of linear range)
43. CD4+ JMos1gp120	0.01	NA	Set % cells < 0.01 to 0.01
44. CD4+ JMos1gp41	0.01	NA	Set % cells < 0.01 to 0.01
45. CD4+ JMos2Sgp120	0.01	NA	Set % cells < 0.01 to 0.01
46. CD4+ JMos2Sgp41	0.01	NA	Set % cells < 0.01 to 0.01
47. CD4+ JMos2Gag	0.01	NA	Set % cells < 0.01 to 0.01
48. CD4+ JMos2RNAseInt	0.01	NA	Set % cells < 0.01 to 0.01
49. CD8+ JMos1gp120	0.01	NA	Set % cells < 0.01 to 0.01
50. CD8+ JMos1gp41	0.01	NA	Set % cells < 0.01 to 0.01
51. CD8+ JMos2Sgp120	0.01	NA	Set % cells < 0.01 to 0.01
52. CD8+ JMos2Sgp41	0.01	NA	Set % cells < 0.01 to 0.01
53. CD8+ JMos2Gag	0.01	NA	Set % cells < 0.01 to 0.01
54. CD8+ JMos2RNAseInt	0.01	NA	Set % cells < 0.01 to 0.01
55. IgG3 V2i breadth score	NA	NA	Same comment as MDW gp120
56. IgG3 V2p breadth score	NA	NA	Same comment as MDW gp120
57. IgG3 V1V2 breadth score trunc1	NA	NA	Before deriving MDW score, Net MFIs truncated between 1 and 22000, and nonresponder Net MFI set to 1. MDW score not truncated.
58. IgG3 V1V2 breadth score selected trunc1	NA	NA	Same comment as for marker 5
59. IgG3 V2i breadth score trunc1	NA	NA	Same comment as for marker 57.
60. IgG3 V2p breadth score trunc1	NA	NA	Same comment as for marker 57.
61. IgG3 1012_11.TC21D11gp120.avi	1	22000	<1 set to 1 (1 = convenient lower value) >22000 set to 2200 (22000 = top of linear range)
62. IgG3 1086C_D7gp120.avi/293F	1	22000	<1 set to 1 (1 = convenient lower value) >22000 set to 2200 (22000 = top of linear range)
63. IgG3 1394C9_G1.D11gp120.avi	1	22000	<1 set to 1 (1 = convenient lower value) >22000 set to 2200 (22000 = top of linear range)
64. IgG3 1428_D11gp120.avi/293F	1	22000	<1 set to 1 (1 = convenient lower value) >22000 set to 2200 (22000 = top of linear range)
65. IgG3 Con 6 gp120/B	1	22000	<1 set to 1 (1 = convenient lower value)

			>22000 set to 2200 (		
			22000 = top of linear range)		
66. IgA Con 6 gp120/B	1	22000	<1 set to 1 (1 =		
			convenient lower value)		
			>22000 set to 2200 (		
			22000 = top of linear range)		
67. IgA 1394C9_G1.D11gp120.avi	1	22000	<1 set to 1 (1 =		
			convenient lower value)		
			>22000 set to 2200 (		
			22000 = top of linear range)		
68. IgA 1428_D11gp120.avi/293F	1	22000	<1 set to 1 (1 =		
			convenient lower value)		
			>22000 set to 2200 (		
			22000 = top of linear range)		
69. IgA 1012_11.TC21D11gp120.avi	1	22000	<1 set to 1 (1 =		
			convenient lower value)		
			>22000 set to 2200 (		
			22000 = top of linear range)		
70. IgA 1086C_D7gp120.avi/293F	1	22000	<1 set to 1 (1 =	1	2
			convenient lower value)		
			>22000 set to 2200 (		
			22000 = top of linear range)		
			convenient lower value)	1	
			convenient lower value)		
			>22000 set to 2200 (		
			22000 = top of linear range)		
			>22000 set to 2200 (		
			convenient lower value)		
			>22000 set to 2200 (		
			22000 = top of linear range)		
			22000 = top of linear range)		
			convenient lower value)		
			>22000 set to 2200 (		
			22000 = top of linear range)		
71. IgA gp140 Mos1 fibritin	1	22000	<1 set to 1 (1 =	1	220
			convenient lower value)		
			>22000 set to 2200 (		
			22000 = top of linear range)		
			convenient lower value)		
			convenient lower value)		
			>22000 set to 2200 (		
			22000 = top of linear range)		
			>22000 set to 2200 (	1	220

			convenient lower value)	
			>22000 set to 2200 (	
			22000 = top of linear range)	
			22000 = top of linear range)	
			convenient lower value)	
			>22000 set to 2200 (	
			22000 = top of linear range)	
72. IgA gp140 C97ZA fibritin	1	22000	<1 set to 1 (1 =	1
			convenient lower value)	
			>22000 set to 2200 (	
			22000 = top of linear range)	
			convenient lower value)	
			convenient lower value)	
			>22000 set to 2200 (	
			22000 = top of linear range)	
			>22000 set to 2200 (	1 22000
			convenient lower value)	
			>22000 set to 2200 (	
			22000 = top of linear range)	
			22000 = top of linear range)	
			convenient lower value)	
			>22000 set to 2200 (	
			22000 = top of linear range)	
73. IgA Con S gp140 CFI	1	22000	<1 set to 1 (1 =	1 220
			convenient lower value)	
			>22000 set to 2200 (	
			22000 = top of linear range)	
			convenient lower value)	1
			convenient lower value)	
			>22000 set to 2200 (	
			22000 = top of linear range)	
			>22000 set to 2200 (	
			22000 = top of linear range)	
74. IgA 1012_gp140C.avi/293F	1	22000	<1 set to 1 (1 =	
			convenient lower value)	
			>22000 set to 2200 (	
			22000 = top of linear range)	
75. IgA 1394C9_gp140C.avi/293F	1	22000	<1 set to 1 (1 =	
			convenient lower value)	
			>22000 set to 2200 (	
			22000 = top of linear range)	
76. IgA 1086C gp140C_avi	1	22000	<1 set to 1 (1 =	
			convenient lower value)	

			>22000 set to 2200 (22000 = top of linear range)
77. IgA BF1266_gp140C.avi/293F	1	22000	<1 set to 1 (1 = convenient lower value) >22000 set to 2200 (22000 = top of linear range)
78. IgA 9004S.gp140C.avi	1	22000	<1 set to 1 (1 = convenient lower value) >22000 set to 2200 (22000 = top of linear range)
79. IgA 00MSA 4076 gp140	1	22000	<1 set to 1 (1 = convenient lower value) >22000 set to 2200 (22000 = top of linear range)
80. IgA A1.con.env03 140 CF	1	22000	<1 set to 1 (1 = convenient lower value) >22000 set to 2200 (22000 = top of linear range)
81. IgA gp70-1012.11.TC21.3257 V1V2	1	22000	<1 set to 1 (1 = convenient lower value) >22000 set to 2200 (22000 = top of linear range)
82. IgA gp70-1394C9G1 V1V2	1	22000	<1 set to 1 (1 = convenient lower value) >22000 set to 2200 (22000 = top of linear range)
83. IgA gp70-BF1266_431a_V1V2	1	22000	<1 set to 1 (1 = convenient lower value) >22000 set to 2200 (22000 = top of linear range)
84. IgA gp70-001428.2.42 V1V2	1	22000	<1 set to 1 (1 = convenient lower value) >22000 set to 2200 (22000 = top of linear range)
85. IgA gp70-Ce1086_B2 V1V2	1	22000	<1 set to 1 (1 = convenient lower value) >22000 set to 2200 (22000 = top of linear range)
86. IgA-Env-breadth score	NA	NA	Same comment as MDW gp120
87. ADCC peak breadth score	NA	NA	Before taking the average of the 3 ADCC peak variables each peak ADCC variable has < 0.01 set to 0.01

88. ADCC pAUC breadth score	NA	NA	Before taking the average of the 3 ADCC pAUC variables each pAUC ADCC variable has < 0.01 set to 0.01
89. ADCP gp70-1428 V1V2			No truncation
90. IgG3 CaseA2 Alter	1	22000	<1 set to 1 (1 = convenient lower value) >22000 set to 2200 (22000 = top of linear range)

16.3 Baseline Markers

Most of the markers listed above that are measured at Month 7 are also measured at baseline, and in some cases are used in the definition of the Month 7 marker.

17 Summary of the Approach to the Data Analyses

The following list summarizes aspects of the correlates data analyses that cut across the study objectives.

1. All CoR analyses control for baseline variables that are hypothesized or observed to be prognostic for HIV-1 acquisition.

2. A baseline behavioral risk score (built by superlearner of the placebo arm data) is one of the baseline variables to systematically adjust for.

3. For all objectives, only participants in the Per Protocol Immunogenicity Cohort (IC-PP) are included.

4. For objectives 3 and 7, all of the analyses are done only in participants assigned to the vaccine arm.

5. Objectives 3, 5–7 are assessed based on a two-phase sampling case-control sampling design.

6. For each objective 3 and 5 separately, multiplicity adjustment is performed on the set of p-values for the hypothesis tests of individual primary markers being correlated with HIV-1 acquisition, vaccine efficacy, and controlled vaccine efficacy, respectively. Westfall-Young (1993) multiplicity adjustment is used as described in Section 18.1.5. Multiplicity adjustment is restricted to the primary markers in order to improve power to detect correlates passing family-wise error rate correction.

7. In all analyses, the HIV-1 diagnosis failure time is right-censored at the minimum of the time of loss-to-follow-up and the right edge of the longest Month 24 visit window, defined as 60 days after the target day for the Month 24 visit (25.91 months).

17.1 Analysis of Objective 3: Month 7 Individual Marker CoRs

The same analyses that are applied for assessing Day 29 markers as immune correlates in the Janssen ENSEMBLE COVID-19 vaccine efficacy trial are employed to meet the objective. In summary, this univariable CoR objective is addressed by Cox proportional hazards regression and nonparametric threshold regression. In addition, supportive exploratory analyses are conducted using flexible parametric regression modeling: generalized additive model regression.

A main choice needed for implementation of these methods is the set of baseline covariates X to adjust for in the analyses. The following four covariates are selected: enrollment in South Africa, BMI, age at enrollment, and baseline behavioral risk score.

17.1.1 Analysis of Objective 3: Month 7 Multivariable Primary Marker CoRs

In addition to the individual-marker CoR analyses, a multivariable Cox model is fit (using the same fitting approach) that includes the 4 Month 7 primary immune markers as quantitative markers, and the same baseline prognostic factors X are adjusted for. Point estimates and 95% confidence intervals are reported for the 4 hazard ratio parameters. An unadjusted p-value from a generalized Wald test for whether the set of 4 markers has any correlation with HIV-1 acquisition is (rejecting the complete null hypothesis that the 4 hazard ratios are all unity) is reported.

17.2 Analysis of Objective 5: Month 7 Individual Marker Controlled VE CoPs

The same analyses that are applied for assessing Day 29 markers as immune correlates in the Janssen ENSEMBLE COVID-19 vaccine efficacy trial are employed to meet the objective, which are based on the Gilbert et al. (2022) method, applied to each of the individual Month 7 markers. The method requires confounding adjustment. The same baseline covariates to adjust for X used for the correlates of risk objective 3 are adjusted for, given the common goal with the use of these covariates.

As a sensitivity analysis, the nonparametric detailed in the USG COVID-19 Response Team / CoVPN Vaccine Efficacy Trial Immune Correlates Statistical Analysis Plan will be applied to the data. This nonparametric approach applies the general estimation framework of Westling and Carone (2020) to estimate the controlled VE curve under the condition the

curve is monotone. Due to availability of code, this sensitivity analysis will take place after the initial set of October correlates analyses.

17.3 Analysis of Objective 6: Month 7 Individual Marker Mediators of VE

The same analyses that are applied for assessing Day 29 markers as immune correlates in the Janssen ENSEMBLE COVID-19 vaccine efficacy trial are employed to meet the objective, which are based on the Benkeser et al. (2021) method. The methods will be applied to assess each Month 7 marker as a mediator, estimating the natural direct effect, the natural indirect effect, and the proportion of VE mediated by the marker, each with a 95% confidence interval. A key identifiability assumption of the method is that within each level of baseline covariates to adjust for X, there are some vaccine recipients with negative readout for the Month 7 marker being analyzed (i.e., the same level of the Month 7 marker that occurs for all placebo recipients). Only Month 7 markers with at least 10% of vaccine recipients with a negative value of the marker will be analyzed.

The method requires confounding adjustment. The same baseline covariates X used for the correlates of risk objective 3 are adjusted for, given the common goal with the use of these covariates.

18 Details of the Data Analyses

18.1 Analysis of Objective 3: Month 7 Individual Marker CoRs

18.1.1 Inverse probability sampling weights used in CoR analyses

This section describes the IPS weight vector that is used for CoR and CoP analyses, $\hat{w}.x$.

For baseline sampling stratum x [(vaccine, placebo) $\times$ (BMI, RSA/Non-RSA)], the IPS weight $\hat{w}.x$ assigned to a non-case participant in stratum x is defined by $\hat{w}.x = 1/\hat{\pi}(x) = N_x/n_x$, where N_x is the number of stratum x participants in the VxControls27 cohort and n_x is the number of these participants in the SelectedVxControls27 cohort. For non-case participant i in the SelectedVxControls27 cohort, $\hat{w}_i = 1/\hat{\pi}(X_i)$ denotes the weight $\hat{w}.x$ for this individual's sampling stratum. All cases are assigned sampling weight N_1/n_1 where N_1 is the total number of vaccine recipient cases in the PPcases7to36, and n_1 is the number of these participants that also had the Month 7 markers measured.

In terms of two-phase sampling data analysis nomenclature, "phase 1 ptids" are defined as the entire IC-PP cohort. The "phase 2 ptids" are then the subset of these phase 1 ptids with Month 7 immune marker data available. The weight $\hat{w}.x$ given to each phase 2 ptid

is the inverse sampling probability weight, calculated as the empirical fraction (No. phase 1 ptids / No. phase 2 ptids) within each of the baseline demographic strata defining the immunogenicity subcohort sampling.

18.1.2 Choice of regression methods

Time-to-event methods use the Month 7 visit date as the time origin.

The inverse-probability weighted complete-case (IPWCC) Cox regression model designed for case-control sampling designs will be used for estimation and inference on hazard ratios of outcomes by Month 7 marker levels, and for estimation and inference on marginalized marker-conditional cumulative incidence over time. The models will be fit using the *survey* R package available on CRAN, and will adjust for the baseline factors. We use a method from the survey package that assumes without replacement two-phase sampling and not Bernoulli sampling, which matches the sampling design and approach to weight estimation (Lumley, 2010).

The final time point t_F of follow-up for correlates analyses is taken to be the latest HIV-1 infection diagnosis outcome event time among vaccine recipient cases. Let T be the failure time, S a Month 7 marker of interest, and X the vector of baseline factors that are adjusted for. With $S_1(t|s,x) = P(T > t|S = s, X = x, A = 1)$, the Cox model fit yields an estimate of $S_1(t|s, X_i)$ for each individual i in the phase-two sample. The marginalized conditional risk $risk_1(t|s) = E_X[P(T \leq t|s, X, A = 1)]$ through time t (for all times t through t_F simultaneously) is estimated based on the equation

$$risk_1(t|s) \quad = \quad \int (1 - S_1(t|s,x))dH(x) \tag{1}$$

where $H(\cdot)$ is the distribution of X in $A = 1$ individuals.

The function $risk_1(t|s)$ can be estimated by

$$\widehat{risk}_1(t|s) = \frac{1}{n_1}\sum_{i=1}^{n_1}(1 - \hat{S}_1(t|s, X_i)), \tag{2}$$

where n_1 is the number of participants with phase-one data / X_i measured (in the primary vaccine arm cohort for correlates analyses).

The bootstrap is used to obtain 95% pointwise confidence intervals for $risk_1(t_F|s)$.

The bootstrap process will be performed by resampling cases, phase 2 controls, and non-phase 2 controls separately. Across bootstrap replicates, the number of cases does not stay constant, neither do the numbers of phase 2 controls by demographics stratum. Specifically, the procedure will 1) sample (with replacement) the original phase 1 data set to obtain dat.b. From dat.b, take only the cases, but also the counts of phase 2 and non-phase 2 controls

by stratum, and 2) sample (with replacement) theses numbers of phase 2 and non-phase 2 controls by stratum from the original data set.

The results of the above Cox modeling will be output in a variety of ways:

1. Plot $\widehat{risk}_1(t_F|s)$ vs. s with 95% CIs for continuous $S = s$ varying over its whole range. Include on the plot the estimate of $\widehat{risk}_0(t_F)$ with a 95% CI for the placebo arm (horizontal bands), computed by a Cox model marginalizing over the same baseline factors as for the analysis of the vaccine arm.

2. Based on a fit of the Cox model to a nominal categorical immune marker defined as the tertiles of S, plot $\widehat{risk}_1(t|s)$ for each category of S values with 95% CIs, for all time points t from Month 7 through t_F. If more than 20% of vaccine recipients have S in the Negative Response category of the assay, then the categories instead will be (1) Negative Response; (2) values below the median of values among those with Positive Response; (3) values above the median of values among those with Positive Response. Include on the plot the estimated curve $\widehat{risk}_0(t)$ with 95% CIs for the placebo arm, computed by a Cox model marginalizing over the same baseline factors as for the analysis of the vaccine arm.

3. Tabular reporting of the hazard ratio per 10-fold change in the quantitative Month 7 immune marker with 95% confidence interval and 2-sided p-value

4. Tabular reporting of the hazard ratio for the Middle and Upper categories of the categorical Month 7 immune marker vs. the Lower category, with 95% confidence interval and 2-sided p-value, as well as a global generalized Wald two-sided p-value for whether the hazard rate of the endpoint varies across the three categories. The table includes the attack rate (with no. of cases / no. at risk) through t_F for each of the three vaccine marker subgroups and for the placebo arm.

5. Report point and 95% CI estimates for the hazard ratio per 10-fold change in the Month 7 immune marker, for the entire IC-PP vaccine cohort and for each of the baseline demographic strata subgroups defined in Table 1 (reported via forest plotting).

6. Westfall-Young (1997) q-values and FWER-adjusted p-values for the generalized Wald tests are included in the table.

Table 1 shows subgroups that are analyzed for the Cox modeling quantitative marker CoR analyses.

The bootstrap is used to calculate 95% pointwise CIs for $risk_1(t_F|s)$ in s. The 2-sided Wald p-value for testing the regression coefficient of the marker in the Cox model provides a valid test of the null hypothesis $H_0 : risk_1(t_F|s) = risk_1(t_F)$ for all s, and is reported.

Table 1: Baseline Subgroups that are Analyzed for Cox Modeling Correlates of Risk.

**Age:** < 25, ≥ 25
**BMI:** < 25, $25 \leq$ BMI < 30, ≥ 30
**Country:** South Africa, not South Africa

In addition, the same Cox model analysis will be used to estimate the alternative marginalized conditional risk parameter defined by $risk_1(t|S \geq s)$ where $risk_1(t|S \geq s) = E_X[P(T \leq t|S \geq s, X, A = 1)]$, which can be estimated by

$$\widehat{risk}_1(t|S \geq s) = \frac{1}{n_1}\sum_{i=1}^{n_1}(1 - \hat{S}_1(t|S \geq s, X_i)). \tag{3}$$

This parameter is useful because typically subgroups of interest are defined by having marker response above a threshold. We will plot $\widehat{risk}_1(t_F|S \geq s)$ vs. s with 95% CIs for continuous S with s varying over the range of S in which the number of cases to estimate $\hat{S}_1(t|S \geq s, X_i)$ is 5 or more. This type of analysis is also included because it analyzes the same parameter as the nonparametric threshold estimation method described below, providing a way to address the threshold question both by Cox modeling and by nonparametric analysis.

18.1.3 Univariate CoR: Nonparametric threshold regression modeling

The van der Laan et al. (2022) extension of the nonparametric CoR threshold estimation method of Donovan et al. (2019) is applied to each of the Month 7 immune markers, using the version accounting for right-censoring of some follow-up times, assessing failure through the fixed time point t_F. The analyses adjust for the same baseline factors X as used in the Cox model CoR analyses.

The extension adjusts for baseline covariates by estimating the conditional mean function $E[I(T \leq t_F)|S \geq s, X, A = 1]$ using discrete-SuperLearner and then empirically averaging over the baseline covariates X to estimate the marginal risk $risk_1(t_F|S \geq s) = E_X[I(T \leq t_F)|S \geq s, X, A = 1]$ for each threshold s of the immune marker in a specified discrete set. We do not perform pooled regression across the thresholds s, which ensures we are totally nonparametric in estimating the threshold dependence of $E_X[I(T \leq t_F)|S \geq s, X, A = 1]$ on s. The SuperLearner library includes a range of increasingly flexible parametric learners including logistic regression (glm), bayesian logistic regression (bayesglm), and L1-penalized logistic regression (glmnet). (Two of each learner is included in the library, one with only main-term variables and another with main-term and interaction variables.)

An advantage of the nonparametric CoR threshold method compared to Cox modeling that specifies a log linear hazard ratio with the marker is that it can potentially detect a threshold of very low risk. The method is implemented with and without the monotonicity constraint that $risk_1^Y(S \geq s)$ is monotone non-increasing in s, where the results assuming monotonicity are reported unless there is evidence for violation of this assumption.

The results are reported in the same way that Donovan et al. (2019) reports results in its Figure 2, where point estimates and simultaneous 95% confidence bands for $risk_1(t_F|S \geq s)$ are plotted for a range of threshold values (the simultaneous confidence bands cover the entire curve in s with at least 95% probability). The method uses the same empirical two-phase sampling estimated weights (IPS weights) as used for the other univariable IPWCC CoR analyses. In addition, for each pre-specified risk threshold c set to take values over a grid between 0 and the estimated outcome rate in placebo recipients, the method is applied to estimate the inverse function $s_c = inf\{s : E_X[I(T \leq t_F)|S \geq s, A = 1, X] \leq c\}$, where s_c is estimated by substitution of the marginal risk function estimate. Note that the substitution estimator of s_c requires that the marginal risk function is estimated for all thresholds, which is computationally infeasible. Instead, we estimate the marginal risk function on a sufficiently large discrete set and linearly interpolate to obtain marginal risk estimates for all thresholds outside the discrete set. In order for this estimand to be well defined, we operate (for this estimand only) under the assumption that $s \mapsto E_X[I(T \leq t_F)|S \geq s, A = 1, X]$ is monotone. For the substitution-based estimator of the inverse function s_c to be well-defined, we require the estimate of $s \mapsto E_X[I(T \leq t_F)|S \geq s, A = 1, X]$ to be monotone as well. If there is evidence that the function estimate is not monotone then we replace the estimate with its monotone projection, which preserves its theoretical properties (Westling and Carone, 2020).

A plot of point and simultaneous 95% confidence interval estimates of s_c (over the grid of c values) is provided to help indicate marker thresholds defining subgroups with very low risk of outcome. The confidence interval estimates for s_c are derived directly from the simultaneous 95% confidence band estimates for the marginal risk function $s \mapsto E_X[I(T \leq t_F)|S \geq s, A = 1, X]$, and therefore its estimates and inference are compatible with those of the marginal risk function. In particular, no multiple testing adjustments are needed.

The analysis is done using targeted maximum likelihood estimation (TMLE) as described in van der Laan et al. (2022), and the simultaneous confidence bands are of the Wald-type, obtained from the asymptotic distribution of the TMLE.

18.1.4 Univariable CoR: Supportive Exploratory Flexible Parametric Risk Modeling

For each of the Month 7 immune markers, flexible nonlinear modeling of outcome risk studied as a dichotomous outcome Y will be conducted, as exploratory supportive analyses. Again, the analyses adjust for the same baseline factors X as used in the Cox model CoR analyses.

18.1.5 P-values and Multiple hypothesis testing adjustment for CoR analysis

For the CoR analyses, p-values are reported for the univariable Cox regression analyses of the set of primary tier Month 7 immune marker variables. Two-sided p-values for hypothesis testing of a Month 7 marker CoR are calculated both for the Cox regression of quantitative

markers (two-sided Wald tests), and for the Cox regression of markers binned into tertiles (two-sided Generalized Wald tests).

A permutation-based method (Westfall et al., 1993) will be used for both family-wise error rate (Holm-Bonferroni) and false-discovery rate (q-values; Benjamini-Hochberg) correction. 10^4 replicates of the data under the null hypotheses will be created by randomly resampling the immune markers with replacement. For each Cox regression CoR analysis the unadjusted p-value, the FWER-adjusted p-value, and the q-value is reported for whether there is a covariate-adjusted association, where all p-values and q-values are 2-sided. The FWER-adjusted p-values and q-values are computed pooling over both the quantitative marker and tertilized marker CoR analyses. As a guideline for interpreting CoR findings, markers with FWER-adjusted p-value ≤ 0.05 are flagged as having statistical evidence for being a CoR. Additionally, markers with unadjusted p-value ≤ 0.05 and q-value ≤ 0.10 are flagged as having a hypothesis generated for being a CoR.

18.1.6 Analysis of Objective 3: Month 7 Primary Multivariable Marker CoRs

The same code as used for individual-marker Cox modeling analysis is applied for the primary-marker multivariable CoR analysis, including the same bootstrapping procedure, use of inverse probability sampling weights, etc.

18.1.7 Exploratory Analysis of Objective 3: Month 7 Multivariable Primary Marker CoRs

The same multivariable Cox model analysis described above is conducted, adding to the 4 Month 7 primary immune markers the IgA-Env-breadth score variable.

18.2 Analysis of Objective 5: Month 7 Individual Marker Controlled VE CoPs

We first describe the controlled vaccine efficacy curve defined as

$$\text{CVE}(s) = 1 - \frac{P(Y(1,s)=1)}{P(Y(0)=1)} \; .$$

The value CVE(s) takes represents the relative decrease in endpoint frequency achieved by administering vaccine and setting Month 7 immunologic marker level to s compared to the placebo control intervention. Under our approach, the value of CVE(s) is assumed to be monotone non-decreasing in s; in other words, vaccine efficacy can only potentially be improved by setting greater marker levels. The extent to which the marker plays a role in determining vaccine efficacy can be determined by the degree of flatness of the graph of CVE(s) versus s.

In addition, because the study cohort for correlates analysis is naive to HIV-1 infection, each of the Month 7 markers S has no variability in the placebo arm [all values are 'negative,' below the assay lower limit of detection (LLOD)]. Therefore, advantageously in this setting $CVE(s)$ has a special connection to the mediation literature (Cowling et al., 2019), where $CVE(s = LLOD)$ is the natural direct effect, and vaccine efficacy is 100% mediated through S if and only if $CVE(s = LLOD) = 0$. Thus inference on $CVE(s = LLOD)$ evaluates full mediation.

Since $P(Y(0) = 1) = P(Y = 1 \mid A = 0)$ in view of vaccine versus placebo randomization, the controlled vaccine efficacy CVE(s) at level s can be identified using the fact that

$$P(Y(1, s) = 1) = E\left[P(Y = 1 \mid S = s, A = 1, X)\right]$$

whenever $Y(1, s)$ and S are independent given $A = 1$ and a vector X of covariates, and $P(S = s \mid A = 1, X) > 0$ almost surely. In other words, identification of the controlled vaccine efficacy requires that a rich enough set of covariates be available so that deconfounding of the relationship between endpoint Y and marker S is possible in the subpopulation of vaccine recipients, and that marker level $S = s$ may occur within each subpopulation defined by values of the covariates X (positivity).

We apply the same Cox modeling approach described in Section 18.1.2 to estimate

$$P(Y(1, s) = 1) = E\left[P(Y = 1 \mid S = s, A = 1, X)\right],$$

augmented with a sensitivity analysis, with advantages of harmonization with the CoR analysis, sensitivity analysis that is generally warranted when a no unmeasured confounders assumption is made, and efficiency gain achieved via the added modeling assumptions. The sensitivity analysis quantifies the rigor of evidence for a controlled VE CoP after accounting for potential bias from unmeasured confounding.

Gilbert et al. (2022) details the sensitivity analysis approach, which was applied to the CYD14 and CYD15 dengue phase 3 data sets citepMoodieetal2018; we plan to apply it in the same way to the HVTN 705 data set (as the structure of the problem is the same). We summarize here the essential details needed for application to the HVTN 705 data set.

We define S to be a controlled risk CoP if $P(Y(1, s) = 1)$ is monotone non-increasing in s with $P(Y(1, s) = 1) > P(Y(1, s') = 1)$ for at least some $s < s'$, where point and 95% confidence interval estimates of $P(Y(1, s) = 1)$ versus s, with built in robustness to unmeasured confounding, describe the strength of the CoP in terms of the amount and nature of decrease. Suppose the CoR analysis based on the Cox model is conducted as described in Section 18.1.2.

Let marginalized conditional risk

$$r_M(s) = risk_1(t_F|s)$$

and controlled risk

$$r_C(s) = P(Y(1, s) = 1).$$

Given that CoR analysis is based on observational data — the biomarker value is not randomly assigned — a central concern is that unmeasured or uncontrolled confounding of the association between S and Y could render $r_M(s) \neq r_C(s)$, biasing estimates of the controlled risk curve $r_C(s)$ and of controlled risk ratios of interest

$$RR_C(s_1, s_2) = r_C(s_2)/r_C(s_1) \ .$$

Because we can never be certain that confounding is adequately adjusted for, sensitivity analysis is warranted, as considered in extensive literature — see, e.g., VanderWeele and Ding (2017) and references therein. Sensitivity analysis is useful to evaluate how strong unmeasured confounding would have to be to explain away an observed causal association, that is, to determine the strength of association of an unmeasured confounder between S and Y needed for the observed exposure-outcome association to not be causal, $r_M(s) \neq r_C(s)$. We follow the recommendation of **?** to report the E-value as a summary measure of the evidence of causality, or, in our application, evidence of whether S is a controlled risk CoP based on variation in the controlled risk curve. We also include other closely related measures of sensitivity.

The E-value is the minimum strength of association, on the risk ratio scale, that an unmeasured confounder would need to have with both the exposure (S) and the outcome (Y) in order to fully explain away a specific observed exposure–outcome association, conditional on the measured covariates [(VanderWeele and Ding, 2017; VanderWeele and Mathur, 2020)]. If, as in CoP analyses, the estimated marginalized risk ratio $\widehat{RR}_M(s_1, s_2) = \widehat{r}_M(s_2)/\widehat{r}_M(s_1)$ for $s_1 < s_2$ is less than one, then the E-value for $\widehat{RR}_M(s_1, s_2)$ is calculated as

$$e_{RR}(s_1, s_2) = \frac{1 + \sqrt{1 - \widehat{RR}_M(s_1, s_2)}}{\widehat{RR}_M(s_1, s_2)} \ . \tag{4}$$

We include the argument (s_1, s_2) in the notation, with $s_1 < s_2$ by convention, to be clear that the E-value depends on specification of two specific marker-level subgroups.

To illustrate the interpretation of an E-value, suppose S is binary and regression analysis yields an estimate $\widehat{RR}_M(0, 1) = \widehat{r}_M(1)/\widehat{r}_M(0) = 0.40$ with 95% confidence interval (CI) $(0.14, 0.78)$. An E-value $e(0, 1)$ of 4.4 means that a marginalized risk ratio $RR_M(0, 1)$ at the observed value 0.40 could be explained away (i.e., $RR_C(0, 1) = 1.0$) by an unmeasured confounder associated with both the exposure and the outcome by a marginalized risk ratio of 4.4-fold each, after accounting for the vector X of measured confounders, but that weaker confounding could not do so.

In addition, we follow the recommendation of VanderWeele and Ding (2017) to also report the E-value $e_{UL}(s_1, s_2)$ for the upper limit $\widehat{UL}(s_1, s_2)$ of the 95% CI for the observed marginalized risk ratio $\widehat{RR}_M(s_1, s_2)$, computed as 1 if $\widehat{UL}(s_1, s_2) \geq 1$ and, otherwise, as

$$\frac{1 + \sqrt{1 - \widehat{UL}(s_1, s_2)}}{\widehat{UL}(s_1, s_2)} \ ,$$

which in the example equals $e_{UL}(0,1) = 1.88$. This E-value for the upper limit indicates, for given $s_1 < s_2$, the strength of unmeasured confounding at which statistical significance of the inference that $RR_C(s_1, s_2) < 1$ would be lost. The two E-values above are useful for judging how confident we can be that an immunologic biomarker is a controlled risk CoP, with E-values near one suggesting weak support and evidence increasing with greater E-values.

$RR_C(s_1, s_2) = (1 - CVE(s_2))/(1 - CVE(s_1))$, evidence for $RR_C(s_1, s_2) < 1$ is equivalently evidence for $CVE(s_1) < CVE(s_2)$. Thus in a placebo-controlled trial $RR_C(s_1, s_2)$ can be interpreted as the multiplicative degree of superior vaccine efficacy caused by marker level s_2 vs. marker level s_1, and E-values equivalently quantify evidence for whether $CVE(s_1)$ differs from $CVE(s_2)$.

It is also useful to provide conservative estimates of controlled risk ratios and of the controlled risk curve, accounting for unmeasured confounding. We approach these tasks based on the sensitivity analysis, or bias analysis, approach of Ding and VanderWeele (2016). We give their main result and refer readers to the paper for details. We begin by defining two (possibly context-specific) fixed sensitivity parameters. First, we set $RR_{UD}(s_1, s_2)$ to be the maximum risk ratio for the outcome Y comparing any two categories of the unmeasured confounders U, within either exposure group $S = s_1$ or $S = s_2$, conditional on the vector X of observed covariates. Second, we set $RR_{EU}(s_1, s_2)$ to be the maximum risk ratio for any specific level of the unmeasured confounder U comparing individuals with $S = s_1$ to those with $S = s_2$, with adjustment already made for the measured covariate vector X. Thus, $RR_{UD}(s_1, s_2)$ quantifies the importance of the unmeasured confounder U for the outcome, and $RR_{EU}(s_1, s_2)$ quantifies how imbalanced the exposure/marker subgroups $S = s_1$ and $S = s_2$ are in the unmeasured confounder U. The values $RR_{UD}(s_1, s_2)$ and $RR_{EU}(s_1, s_2)$ are always specified as greater than or equal to one. We suppose that $RR_M(s_1, s_2) < 1$ for the fixed values $s_1 < s_2$ — this is the case of interest for immune correlates.

Define the bias factor

$$B(s_1, s_2) = \frac{RR_{UD}(s_1, s_2) RR_{EU}(s_1, s_2)}{RR_{UD}(s_1, s_2) + RR_{EU}(s_1, s_2) - 1}$$

for $s_1 \leq s_2$, and define $RR_M^U(s_1, s_2)$ the same way as $RR_M(s_1, s_2)$, except marginalizing over the joint distribution of X and U. Then, $RR_M^U(s_1, s_2) \leq RR_M(s_1, s_2) \times B(s_1, s_2)$, where $RR_M^U(s_1, s_2) = E\{r(s_2, X^*)\}/E\{r(s_1, X^*)\}$ with $X^* = (X, U)$ and $r(s, x^*) = P(Y = 1 \mid S = s, A = 1, X = x, U = u)$ (Ding and VanderWeele, 2016).

Translating this result to our problem context, under the positivity assumption, we have that $RR_M^U(s_1, s_2) = RR_C(s_1, s_2)$ and so, it follows that

$$RR_C(s_1, s_2) \leq RR_M(s_1, s_2) \times B(s_1, s_2) \; . \tag{5}$$

This inequality states that the causal risk ratio is bounded above by the marginalized risk ratio multiplied by the bias factor. It follows that a conservative (upper bound) estimate

of $RR_C(s_1, s_2)$ is obtained as $\widehat{RR}_M(s_1, s_2) \times B(s_1, s_2)$, and a conservative 95% CI is obtained by multiplying each confidence limit for $RR_M(s_1, s_2)$ by $B(s_1, s_2)$. These estimates for $RR_C(s_1, s_2)$ account for the presumed-maximum plausible amount of deviation from the no unmeasured confounders assumption specified by $RR_{UD}(s_1, s_2)$ and $RR_{EU}(s_1, s_2)$. An appealing feature of this approach is that the bound (5) holds without making any assumption about the confounder vector X or the unmeasured confounder U.

The above approach does not directly provide a conservative estimate of the controlled risk curve $r_C(s)$, because additional information is needed for absolute versus relative risk estimation. To provide conservative inference for $r_C(s)$, we next select a central value s^{cent} of S such that $\widehat{r}_M(s^{cent})$ matches the observed overall risk, $\widehat{P}(Y = 1|A = 1)$. This value is a 'central' marker value at which the observed marginalized risk equals the observed overall risk. Next, we 'anchor' the analysis by assuming $r_C(s^{cent}) = r_M(s^{cent})$, where picking the central value s^{cent} makes this plausible to be at least approximately true. Under this assumption, the bound (5) implies the bounds

$$r_C(s) \leq r_M(s)B(s^{cent}, s) \quad \text{if } s \geq s^{cent} \qquad (6)$$

$$r_C(s) \geq r_M(s)\frac{1}{B(s, s^{cent})} \quad \text{if } s < s^{cent}. \qquad (7)$$

Therefore, after specifying $B(s^{cent}, s)$ and $B(s^{cent}, s)$ for all s, we conservatively estimate $r_c(s)$ by plugging $\widehat{r}_M(s)$ into the formulas (6) and (7). Because $B(s_1, s_2)$ is always greater than one for $s_1 < s_2$, formula (6) pulls the observed risk $\widehat{r}_M(s)$ upwards for subgroups with high biomarker values, and formula (7) pulls the observed risk $\widehat{r}_M(s)$ downwards for subgroups with low biomarker values. This makes the estimate of the controlled risk curve flatter, closer to the null curve, as desired for a sensitivity/robustness analysis.

To specify $B(s_1, s_2)$, we note that it should have greater magnitude for a greater distance of s_1 from s_2, as determined by specifying $RR_{UD}(s_1, s_2)$ and $RR_{EU}(s_1, s_2)$ increasing with $s_2 - s_1$ (for $s_1 \leq s_2$). We consider one specific approach, which sets $RR_{UD}(s_1, s_2) = RR_{EU}(s_1, s_2)$ to the common value $RR_U(s_1, s_2)$ that is specified log-linearly: $\log RR_U(s_1, s_2) = \gamma(s_2 - s_1)$ for $s_1 \leq s_2$. Then, for a user-selected pair of values $s_1 = s_1^{fix}$ and $s_2 = s_2^{fix}$ with $s_1^{fix} < s_2^{fix}$, we set a sensitivity parameter $RR_U(s_1^{fix}, s_2^{fix})$ to some value above one. It follows that

$$\log RR_U(s_1, s_2) = \left(\frac{s_2 - s_1}{s_2^{fix} - s_1^{fix}}\right) \log RR_U(s_1^{fix}, s_2^{fix}), \qquad s_1 \leq s_2.$$

We anchor the sieve analysis by setting $s_1 = s_1^{fix}$ at the 15th percentile of the Month 7 immune marker and $s_2 = s_2^{fix}$ at the 85th percentile of the Month 7 immune marker.

The sensitivity analysis is done for each of the two Cox model CoR analyses described in Section 18.1.2, first for tertiles of the Month 7 marker and second for the quantitative marker. For the former, E-values are reported for both the point estimate and the upper 95% confidence limit for $RR_C(0, 1)$, where category 1 is the upper tertile, category 0 is the

lower tertile, and the intermediate middle tertile subgroup of vaccine recipients is excluded from the analysis. In addition, setting $RR_{UD}(0,1) = RR_{EU}(0,1) = 2$, such that $B(0,1) = 4/3$, we report conservative estimation and inference on the causal risk ratio $RR_C(0,1)$ and equivalently on the ratio of controlled vaccine efficacy curves $(1 - CVE(1))/(1 - CVE(0))$.

Next we repeat the analysis treating S as a quantitative variable, where $P(T \leq t|S = s, X, A = 1)$ is again estimated by two-phase Cox partial likelihood regression and now $RR_M(s_1, s_2)$ is the marginalized risk ratio between s_1 and s_2. We will plot point and 95% confidence interval estimates of the observed marginalized risk and controlled risk curves, for the latter using the sensitivity analysis described in Section 18.2.

For validity the method requires the positivity assumption, and thus the method will only be applied if the data are reasonably supportive of the positivity assumption. To check positivity, we study the immune marker distribution in vaccine recipients within each subgroup of the covariates X that are adjusted for. For the tertiles analysis we require evidence that within each subgroup some vaccine recipients have lower tertile responses and some vaccine recipients have upper tertile responses. For the quantitative S analysis, we look for evidence that S varies over its full range within each level of the potential confounders that are adjusted for.

18.3 Analysis of Objective 6: Month 7 Individual Marker Mediators of VE

A classic application of mediation is to decompose the overall VE into so-called *natural* direct and indirect effects. We will estimate this decomposition for each Month 7 immune marker individually.

For simplicity, as before, we describe this approach using a binary outcome, noting that extensions to time-to-event (with competing risks) are possible. The *total* effect of the vaccine can be represented by the risk ratio RR = (1 - VE),

$$\text{RR} = \frac{P(Y(1, S(1)) = 1)}{P(Y(0, S(0)) = 1)} .$$

The natural direct and indirect effects are, respectively,

$$\text{RR}_{DE} = \frac{P(Y(1, S(0)) = 1)}{P(Y(0, S(0)) = 1)} \quad \text{and} \quad \text{RR}_{IDE} = \frac{P(Y(1, S(1)) = 1)}{P(Y(1, S(0)) = 1)} .$$

Note that $\text{RR} = \text{RR}_{DE}\text{RR}_{IDE}$, showing that the total effect decomposes into the direct times indirect effect. Another quantity of interest is the proportion mediated, which we express as

$$\text{PM} = 1 - \frac{\log(\text{RR}_{DE})}{\log(\text{RR})} .$$

We note that PM=1 if and only if $\text{RR}_{DE} = 1$, i.e., no direct effect means that the marker fully mediates VE. We will estimate PM defined in this way.

As above, we must assume all confounders X of S and Y have been measured. We also assume there is sufficient overlap of the immunologic marker distributions, and no confounders of the mediator-outcome relationship that are affected by treatment. Moreover, we require the assumption

$$P(S = s|A = 0, X = x) > 0 \quad \text{implies} \quad P(S = s|A = 1, X = x) > 0 \tag{8}$$

for all subgroups $X = x$ (i.e., a.e.). Under these assumptions, $P(Y(a, S(a') = 1)$ is identified by

$$E[P(Y = 1 \mid A = a, S, X)|A = a', X] \ .$$

In our immune CoP application it is expected that, because the analysis cohort restricts to HIV-1 negative individuals, the conditional density of the immune marker in the placebo arm will be a point mass at 0, that is with S taking the value Negative Response. In other words, we do not expect any placebo recipients to have a positive value of the immune response marker. This implies the identification result that for $a = 0, 1$, $P(Y(a, S(0)) = 1) = E[P(Y = 1 \mid A = a, S = 0, X)]$. While $P(Y(0, S(1) = 1)$ is not identified, it is not necessary to estimate this term in order for estimation of the parameters of interest (natural direct effect, natural indirect effect, PM).

For a highly immunogenic vaccine, it may be the case that the needed overlap assumption (8) will be violated. This could happen, for example if each placebo recipient has immune marker value Negative Response (which is expected), and every vaccine recipient has immune marker value Positive Response. We will only include immune markers for mediation analysis if at least 10% of vaccine recipients have marker value equal to the value in placebo recipients.

Benkeser et al. (2021) provide a multiply robust targeted minimum loss-based plug-in estimator of natural direct and indirect effects that is appropriate for case-control sampling. The estimator requires estimation of several regressions, which are used in an augmented inverse probability of treatment weighted estimator. The propensity score will be estimated by a main terms logistic regression model to account for chance imbalances across randomization arms. The sequential outcome regressions used by the approach will be based on a super learner with the 14 algorithms listed in Table 2.

The estimator is implemented in the `natmed2` package available on GitHub (https://github.com/benkeser/natmed2). The baseline covariates X adjusted for are the same as for the other analyses (i.e. of CoR and of controlled vaccine efficacy).

If there are fewer than 100 observed HIV-1 infection endpoint cases (pooled over the randomization arms), then we will leverage logistic and linear regression models, as appropriate, to estimate each of the above regressions and only include a low-dimensional set of pre-specified

Table 2: Learning Algorithms in the super learner Library for mediation methods[1].

Algorithms	Screens[2]/ Tuning Parameters
SL.mean	All
SL.glm	Low-collinearity and (All, Lasso, LR)
SL.glm.interaction	(All, Lasso, LR)
SL.gam	Low-collinearity and (Lasso, LR)
SL.glmnet	All
SL.xgboost	All
SL.ranger	All

[1] some nuisance parameters have binary outcomes, others quantitative. For the former, we used `family = binomial()` input to the `SuperLearner` function; for the latter, we used `family = gaussian()`.

[2]**All** = include all variables; **Lasso** = include variables with non-zero coefficients in the standard implementation of SL.glmnet that optimizes the lasso tuning parameter via 10-fold cross-validation; **Low-collinearity** = do not allow any pairs of quantitative variables with Spearman rank correlation > 0.90; **LR** = Univariate logistic regression Wald test 2-sided p-value < 0.10.

characteristics in X. In these cases, 95% confidence intervals for RR_{DE}, RR_{IDE} and PM will be constructed using the percentile-based nonparametric bootstrap.

However, it is known that there are more than 100 HIV-1 infection endpoint cases (pooled over the randomization arms), a scenario for which we will instead employ super learning to estimate the above regression quantities and include a higher dimensional set of potential confounders in X; the same set of potential baseline potential confounders input into the superlearner modeling of the placebo arm for building a behavioral risk score. In this case, the super learner library includes a diversity of pre-specified algorithms. The nonparametric bootstrap cannot be used to construct confidence intervals, and we will instead rely on Wald-style confidence intervals with standard errors estimated based on the empirical variance of the estimators' estimated influence functions.

See Benkeser et al. (2021) for additional details about the mediation method that is applied to the data.

19 Handling missing data

We expect a small amount of missing data from the Month 7 immune markers. However, there may be a small amount of missing data, with possibly different participants missing data for different markers. We take the following approach to handle any missing data that occurs.

First, we define the two-phase sampling indicator ϵ as taking value of one if a participant has data available at Month 7 for the three primary immune markers ELISA VT-C data, ADCP gp140 C97ZA antigen, and IgG3 V1V2 breadth score. Second, for all other immune markers, for participants with $\epsilon = 1$ but the Month 7 marker value is missing, we use single imputation to fill in any missing values, ignoring the uncertainty in the imputations in the analysis, because it should have negligible impact on results given the (very) small amount of missing data. Multiple linear regression will be used to impute missing values, separately for each immune marker, based on the set of individuals with that immune marker measured at Month 7. This process means that the two-phase data set has a simple 'all-or-nothing' missing data pattern where participants with $\epsilon = 1$ have all markers with Month 7 data, and are included in IPWCC analyses, and participants with $\epsilon = 0$ have some or all markers missing and are excluded from IPWCC analyses. This means that all IPWCC data analyses can use the same empirical frequency (IPS) sampling weights.

20 Plan for determining the dilution of the ADCP for defining the ADCP biomarker

The Month 7 ADCP biomarker will be defined as the phagocytic score, calculated at either a 1:25 or 1:250 sample dilution. To select the sample dilution, for the first of the three batches of case-control samples, the ADCP assay will be performed at both serum dilutions, generating a paired data set of phagocytic scores. These data will be analyzed to compare the two scores by their estimated signal-to-noise ratio, defined as the ratio of biological variability over technical variability. The technical variability will be estimated as the median of the variances across $M = 2$ technical replicates for each test sample, and the biological variability will be estimated as the variance of the average of the M technical replicates across all test samples minus the technical variability. The Month 7 phagocytic score with the highest estimated signal-to-noise ratio will be selected as the biomarker to use in the correlates analysis, which means that for the second and third batches of case-control samples, the ACDP assay will be run at the selected serum dilution. This selection process is based on a data set of vaccine recipients that is blinded to case/non-case status, which implies the process does not impact the integrity of the correlates analysis. Moreover, because the selection process is blinded to case/non-case status, the decision is not absolutely determined by the ranking by estimated signal-to-noise ratio; other factors may be taken into account.

A data analysis report was produced, and the 1:250 dilution selected.

20.1 Notes on the ADCP biomarkers

The two ADCP biomarkers assessed as correlates are average phagocytosis scores, without truncation at assay limits such as the LOD, LLOQ, and the ULOQ. (Only one ADCP

biomarker is available for the first correlates analysis.) Month 7 positive response is defined as average phagocytosis score $\geq$ 95th percentile of baseline average phagocytosis scores, defined using all case-control participants pooling over cases, non-cases, vaccine, and placebo at dilution 1:250.

20.2 Notes on the ELISpot PTE Env marker

The ELISpot PTE Env marker is analyzed descriptively and is not analyzed for correlates of risk and correlates of protection analyses, as noted above.

The readout for ELISpot PTE Env is the $\log_{10}$ transformed sum of the number of spot forming cells (SFC)/ 10^6 PBMCs for PTE Env 1, PTE Env 2, and PTE Env 3. The limit of detection (LOD) for each PTE pool 1, 2, 3 is 60 SFC/10^6 PBMC. A positive response is defined by Value $\geq$ LOD, where

$$\text{Value} = \sum_{k=1}^{3} \{\text{I(Result PTE Env k } \geq \text{LOD)*(Result PTE Env k)}\}\,.$$

If Value $\geq$ LOD then the response is deemed positive; otherwise it is deemed negative and Value is set to LOD/2.

References

Benkeser, D., Diaz, I., and Ran, J. (2021), "Inference for natural mediation effects under case-cohort sampling with applications in identifying COVID-19 vaccine correlates of protection," *arXiv*, arXiv:2103.02643 [q-bio.QM] [Preprint] March 5 2021. Cited 13 March 2021. Available from https://arxiv.org/pdf/2103.02643.pdf.

Cowling, B., Lim, W., Perera, R., Fang, V., Leung, G., Peiris, J., and Tchetgen Tchetgen, E. (2019), "Influenza hemagglutination-inhibition antibody titer as a mediator of vaccine-induced protection for influenza B," *Clinical Infectious Diseases*, 68(10), 1713–7.

Ding, P. and VanderWeele, T. (2016), "Sensitivity analysis without assumptions," *Epidemiology*, 27(3), 368.

Donovan, K., Hudgens, M., and Gilbert, P. B. (2019), "Nonparametric inference for immune response thresholds of risk in vaccine studies," *Annals of Applied Statistics*, 13, 1147–1165, pMCID: PMC6613658 [Delayed release (embargo): Available on 2020-06-01].

Fong, Y., Shen, X., Ashley, V., Deal, A., Seaton, K., Yu, C., Grant, S. S., Ferrari, G., deCamp, A., Bailer, R., Koup, R., Montefiori, D., Haynes, B., Sarzotti-Kelsoe, M., Graham, B., Carpp, L., Hammer, S., Sobieszczyk, M., Karuna, S., Swann, E., DeJesus, E., Mulligan, M., Frank, I., Buchbinder, S., Novak, R., McElrath, M., Kalams, S., Keefer, M., Frahm, N., Janes, H., Gilbert, P. B., and Tomaras, G. (2018), "Modification of the Association Between T-Cell Immune Responses and Human Immunodeficiency Virus Type 1 Infection Risk by Vaccine-Induced Antibody Responses in the HVTN 505 Trial," *Journal of Infectious Diseases*, 217, 1280–1288, pMCID: PMC6018910.

Gilbert, P. B., Fong, Y., Kenny, A., and Carone, M. (2022), "A controlled effects approach to assessing immune correlates of protection," *Biostatistics*, kxac024.

Haynes, B., Gilbert, P. B., McElrath, M., and et al. (2012), "Immune correlates analysis of the ALVAC-AIDSVAX HIV-1 vaccine efficacy trial," *New England Journal of Medicine*, 366, 1275–1286.

He, Z. and Fong, Y. (2019), "Maximum diversity weighting for biomarkers with application in HIV-1 vaccine studies," *Statistics in medicine*, 38, 3936–3946.

Janes, H. E., Cohen, K. W., Frahm, N., De Rosa, S. C., Sanchez, B., Hural, J., Magaret, C. A., Karuna, S., Bentley, C., Gottardo, R., Finak, G., Grove, D., Shen, M., Graham, B. S., Koup, R. A., Mulligan, M. J., Koblin, B., Buchbinder, S. P., Keefer, M. C., Adams, E., Anude, C., Corey, L., Sobieszczyk, M., Hammer, S. M., Gilbert, P. B., and McElrath, M. J. (2017), "Higher T-cell responses induced by DNA/rAd5 HIV-1 preventive vaccine are associated with lower HIV-1 infection risk in an efficacy trial," *The Journal of Infectious Diseases*, 215, 1376–1385, pMCID: PMC5853653.

Li, S. S., Gilbert, P. B., Tomaras, G. D., Kijak, G., Ferrari, G., Thomas, R., Zolla-Pazner, S., Evans, D. T., Yao, L., Gottardo, R., Dai, J. Y., Janes, H. E., Morris, D., Fong, Y., Edlefsen, P., Li, F., Magaret, C. A., Frahm, N., Alpert, M. D., Rerks-Ngarm, S., Pitisuttithum, P., Kaewkungwal, J., Nitayaphan, S., Robb, M. L., O'Connell, R. J., Michael, N. L., Kim, J. H., McElrath, M. J., and Geraghty, D. E. (2013), "Association of Fc-γRIIC Polymorphism with Vaccine Efficacy and Correlate of HIV-1 Infection Risk in RV144. AIDS Vaccine 2013 International Conference." Barcelona, Spain, 2013, abstract PL04.05.

Lumley, T. (2010), *Complex surveys: a guide to analysis using R*, vol. 565, John Wiley & Sons.

Neidich, S. D., Fong, Y., Li, S. S., Geraghty, D. E., Williamson, B. D., Young, W. C., Goodman, D., Seaton, K. E., Shen, X., Sawant, S., et al. (2019), "Antibody Fc effector functions and IgG3 associate with decreased HIV-1 risk," *Journal of Clinical Investigation*, 129, 4838–4849.

van der Laan, L., Zhang, W., and Gilbert, P. B. (2022), "Efficient nonparametric estimation of the covariate-adjusted threshold-response function, a support-restricted stochastic intervention." *Biometrics*, in press.

VanderWeele, T. and Ding, P. (2017), "Sensitivity analysis in observational research: introducing the E-value," *Annals of Internal Medicine*, 167(4), 268–74.

VanderWeele, T. and Mathur, M. (2020), "Commentary: developing best-practice guidelines for the reporting of E-values," *International Journal of Epidemiology*, 49(5), 1495–1497.

Westfall, P. H., Young, S. S., et al. (1993), *Resampling-based multiple testing: Examples and methods for p-value adjustment*, vol. 279, John Wiley & Sons.

Westling, T. and Carone, M. (2020), "A unified study of nonparametric inference for monotone functions," *Annals of Statistics*, 48, 1001–1024.

Yates, N. L., Liao, H.-X., Fong, Y., DeCamp, A., Vandergrift, N. A., Williams, W. T., Alam, S. M., Ferrari, G., Yang, Z.-y., Seaton, K. E., et al. (2014), "Vaccine-induced Env V1-V2 IgG3 correlates with lower HIV-1 infection risk and declines soon after vaccination," *Science translational medicine*, 6, 228ra39–228ra39.

21 Appendix: Notes for Statisticians Implementing the Immune Correlates Code Suite

README documentation of the variables is at:

`T:\vaccine\p705\analysis\lab\cc\copcor`

The correlates analysis reports [coxph (Youyi), np-threshold (Lars), nonparametric controlled VE (Avi), mediation (David), multivariable CoR/Superlearner (Bhavesh)] are written to:

`T:\vaccine\p705\analysis\lab\cc\copcor\correlates\_reports`
